# Mechanism of traditional Chinese medicine in elderly diabetes mellitus and a systematic review of its clinical application

**DOI:** 10.3389/fphar.2024.1339148

**Published:** 2024-03-06

**Authors:** Qiqi Zhang, Shiwan Hu, Zishan Jin, Sicheng Wang, Boxun Zhang, Linhua Zhao

**Affiliations:** ^1^ Institute of Metabolic Diseases, Guang’anmen Hospital, Chinese Academy of Chinese Medical Sciences, Beijing, China; ^2^ Graduate School, Beijing University of Chinese Medicine, Beijing, China; ^3^ Department of Endocrinology, Hospital of Chengdu University of Traditional Chinese Medicine, Chengdu, China

**Keywords:** elderly diabetes mellitus, traditional Chinese medicine, hypoglycemia, vascular aging, cognitive impairment, osteoporosis, sarcopenia, systematic review

## Abstract

**Objective:** Affected by aging, the elderly diabetes patients have many pathological characteristics different from the young people, including more complications, vascular aging, cognitive impairment, osteoporosis, and sarcopenia. This article will explore their pathogenesis and the mechanism of Traditional Chinese medicine (TCM) intervention, and use the method of systematic review to evaluate the clinical application of TCM in elderly diabetes.

**Method:** Searching for randomized controlled trials (RCTs) published from January 2000 to November 2023 in the following databases: Web of Science, Pubmed, Embase, Cochrane Library, Sinomed, China National Knowledge Internet, Wanfang and VIP. They were evaluated by three subgroups of Traditional Chinese Prescription, Traditional Chinese patent medicines and Traditional Chinese medicine extracts for their common prescriptions, drugs, adverse reactions and the quality of them.

**Results and Conclusion:** TCM has the advantages of multi-target and synergistic treatment in the treatment of elderly diabetes. However, current clinical researches have shortcomings including the inclusion of age criteria and diagnosis of subjects are unclear, imprecise research design, non-standard intervention measures, and its safety needs further exploration. In the future, the diagnosis of elderly people with diabetes needs to be further clarified. Traditional Chinese patent medicines included in the pharmacopoeia can be used to conduct more rigorous RCTs, and then gradually standardize the traditional Chinese medicine prescriptions and traditional Chinese medicine extracts, providing higher level evidence for the treatment of elderly diabetes with traditional Chinese medicine.

## 1 Introduction

Diabetes mellitus is a highly prevalent health condition in the aging population. With the aging degree of the population increasing in the past 50 years, the number of older adults (≥65 years old) living with diabetes is expected to grow rapidly in the coming decades and has become the mainstream population of diabetes. Over 25% of people over the age of 65 years have diabetes, and 50% of older adults have prediabetes (Laiteerapong and Huang, 2018; Prevention, 2020). The prevalence of diabetes in adults aged 75–79 years in 2021 is estimated at 24.0% and is expected to rise to 24.7% in 2045 (IDF, 2021). There are 122.8 million people aged 65–99 years with diabetes worldwide and that number is projected to grow dramatically to 253.4 million in 2045 (IDF, 2017).

It is worth noting that although many available treatment methods can still be considered in healthy elderly individuals when combining hypoglycemic agents to achieve recommended goals, the combination with the lowest risk of hypoglycemia should be considered. Hence, the selection of appropriate hypoglycemic drugs is limited for elderly patients with frailty. In recent years, plant-derived traditional herbal medicine and its phytochemicals have attracted people’s attention as a kind of nutrient to prevent the onset and progress of diabetes and its serious complications. Compared with Western medicine, traditional herbal medicine has many advantages in the prevention and treatment of elderly type 2 Diabetes mellitus (T2DM).

It is important to consider the risk of hypoglycemia when combining hypoglycemic agents to achieve recommended goals in healthy elderly individuals. However, the options for selecting appropriate hypoglycemic drugs are limited for frail elderly patients. In recent years, Traditional Chinese medicine (TCM) has gained attention as a nutritional approach to prevent and manage diabetes and its complications. TCM offers several advantages over Western medicine in the context of elderly T2DM management.

Firstly, TCM allows for individualized clinical therapy based on different conditions and constitutions (Zhou et al., 2014a). It views diseases as imbalances within the whole individual rather than isolated organ lesions, emphasizing the regulation of internal and external balance within the body (Qiu, 2007). Secondly, given that diabetes involves complex metabolic disorders and often requires multiple drug treatments, which may increase the risk of hypoglycemia, especially among elderly patients taking sulfonylurea drugs or insulin injections. Traditional herbal medicine can comprehensively regulate bodily functions and support normal glucose metabolism. Importantly, it can replace some pharmaceuticals with severe contraindications for elderly patients, offering better tolerance. Therefore, at present, many elderly diabetic patients who have no obvious response or intolerance to hypoglycemic effects from Western medicine prefer to choose alternative treatment methods, such as herbal medicine or TCM, thus making alternative treatment for diabetes a popular treatment method.

As a result, many elderly diabetic patients who do not respond well to Western medicine or face intolerance to its side effects prefer alternative treatments like herbal medicine or TCM. TCM therapy, with its characteristics of comprehensive regulation, multi-target effects, and personalized medication, has shown remarkable therapeutic efficacy, minimal adverse effects, and a commendable safety profile (Meng et al., 2023). Numerous clinical and basic research studies have provided evidence of TCM’s clinical effectiveness in managing diabetes, regardless of age (Shao et al., 2021). For example, in a double-blind, randomized, placebo-controlled study involving 420 patients with impaired glucose tolerance (IGT), it was found that the combination of Tianqi capsule and lifestyle intervention for 12 months reduced the risk of diabetes by 32.1% (Lian et al., 2014). Notably, TCM has a positive impact on elderly diabetes patients by effectively lowering blood glucose levels, reducing the progression of diabetes complications and comorbidities, and significantly extending the lifespan of elderly individuals (Tian et al., 2019). This article reviews the clinical features of elderly diabetes mellitus and the latest clinical applications of TCM in managing elderly diabetes and its complications. It aims to provide insights into supplementary and alternative medicine in the clinical management of chronic diseases in the elderly.

## 2 The pathological mechanisms in elderly diabetes mellitus

**FIGURE 1:**
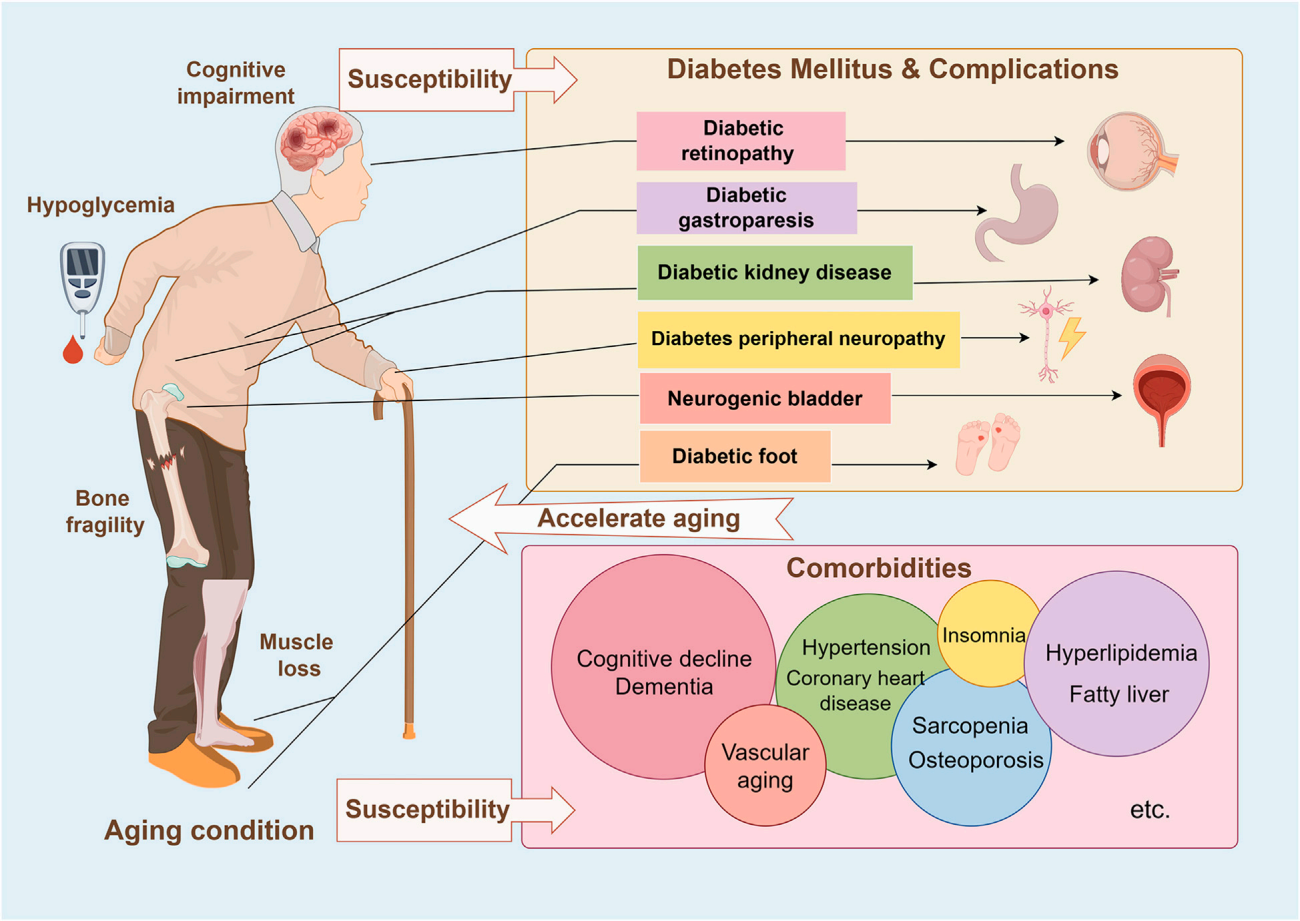
During the aging process of the elderly, the β-cells gradually secrete insufficient insulin, accompanied by insulin resistance, leading to an increased risk of diabetes. After diabetes, it can further develop into diabetes complications, such as diabetes retinopathy, diabetes gastroparesis, diabetic kidney disease, diabetes peripheral neuropathy, neurogenic bladder, diabetes foot, etc. At the same time, elderly diabetes patients themselves are prone to hypoglycemia, which is more likely to lead to cardiovascular and cerebrovascular events, and long-term repeated hypoglycemia is easy to cause neurodegenerative diseases. The elderly are also often accompanied by sarcopenia, osteoporosis, etc., and metabolic syndrome can also occur with the decline of metabolic capacity. Elderly diabetes, a series of diabetic complications, and multiple comorbidities will further accelerate the aging of multiple organs, leading to further increased bone fragility, vascular aging, muscle loss, cognitive decline, etc.

Unlike young patients with diabetes, diabetes in older adults is a highly heterogeneous condition, and diabetic individuals who become old have different characteristics compared to older individuals who become diabetic on functional status, comorbidities, and degree of frailty (Laiteerapong and Huang, 2018) and face particularly difficult challenges (Figure 1). Clinical research has found that older adults with T2DM are frequently associated with cardio-renal challenges and are more likely to have the risk of hypoglycemia in their frail body as well as merge multiple complications and comorbidities which seriously affect the quality of life and lifespan of the elderly (Bellary et al., 2021). The following briefly discusses some clinical features of senile diabetes and outlines its main pathological mechanism.

### 2.1 The aging process and declining pancreatic function in elderly individuals

As the metabolic capacity of the elderly declines, multiple metabolic disturbances combined with vascular aging contribute to the increasing prevalence of vascular complications in diabetes year by year. Aging increases the susceptibility to T2DM. In both humans and rodent models, glucose-stimulated insulin secretion appears to decrease with advancing age. In humans, this reduction, to some extent, may be associated with decreased expression and function of the GLUT-2 transporter, as well as diminished glucose oxidation (Sun et al., 2023). Furthermore, inadequate inhibition of K^+^ efflux and reduced Ca^2+^ uptake (required for insulin granule exocytosis) have been implicated in aging rodent models; however, current human data remain limited. Additionally, a novel contributor to islet cell injury is islet amyloid polypeptide, which is oversecreted along with insulin in insulin-resistant states. This excessive secretion leads to aggregation and amyloid plaque formation, consequently inducing cell apoptosis. This process is particularly prominent in elderly diabetic patients (Gunasekaran and Gannon, 2011).

### 2.2 The mechanisms of vascular complications in elderly diabetes

Older individuals are more susceptible to the early onset of diabetes vascular complications (Bellary et al., 2021). On the one hand, metabolic disturbances affect vascular endothelial cells, including disruptions in glucose metabolism, lipid metabolism, intestinal microbiota metabolism, inflammation-related metabolites, and the impact of arachidonic acid derivatives on the endothelium (Xue et al., 2023). On the other hand, under the regulation of various cells and their secreted cytokines, key transcriptional regulation pathways such as TLR2/4-NF-κB, p38/MAPK, IL-6/STAT3, and others participate in the immune-inflammatory interactions underlying diabetic vascular complications, ultimately leading to vascular damage and barrier disruption, triggering diabetes-related macrovascular and microvascular complications (Odegaard et al., 2016; Wu et al., 2018).

Specifically, in the context of diabetic nephropathy, high glucose-induced metabolic disturbances and hemorheology lead to impaired renal function. Activation of the Renin-Angiotensin-Aldosterone System (RAAS) results in consequences like glomerular hyperperfusion, hypertension, and high filtration. Simultaneously, there are disruptions in the expression of signaling pathways such as Transforming growth factor-β (TGF-β) signaling pathways, VEGF/VEGFR signaling pathways, Angiopoietin (Ang)/Tie signaling pathways, among others, ultimately driving glomerular fibrosis and progressing towards end-stage pathological conditions like glomerulosclerosis.

Diabetic retinopathy, similarly, is one of the microvascular complications of diabetes. However, due to the high metabolic demand of retinal cells and limited vascular supply, they are particularly sensitive to metabolic disturbances. Structural vascular disruptions are more pronounced in Diabetic Retinopathy (DR) (Yang and Liu, 2022).

Regarding diabetes-related coronary heart disease, it is mainly due to the fact that glucose metabolism disturbances may alter and increase the impact of other risk factors for atherosclerosis. For example, low-density lipoprotein (LDL) is more susceptible to modifications by Advanced Glycation End Products (AGEs) in late-stage glycation. Increased lipoprotein oxidation, increased LDL receptor uptake of LDL protein, and increased platelet aggregation are also disrupted (Madonna et al., 2018). A characteristic feature of macrovascular complications in diabetes is the formation of new blood vessels within plaques. Due to excessive or aberrant neovascularization, there is an increase in capillary density, tissue edema, leading to more frequent arterial atherosclerotic plaque hemorrhage and plaque rupture, along with microvascular dysfunction in the heart (Madonna et al., 2018).

Iron death is a newly discovered iron-dependent regulation of cell death. Research indicates that iron death plays an important role in the pathophysiology and pathogenesis of diabetes and its related complications (Liu et al., 2024). Furthermore, emerging evidence suggests that extracellular vesicle (EVs)-mediated crosstalk between pancreatic islet cells and between organs is involved in the progression of diabetes. EVs derived from β-cells can also affect recipient β-cells and further exert negative effects through autocrine signaling in type 2 diabetes (Wei et al., 2023).

### 2.3 The pathological mechanisms of comorbidities in elderly diabetes

With the aging of multiple organs, elderly diabetes is often accompanied by other chronic comorbidities. Here, we mainly discuss the pathological mechanisms of several common diabetic comorbidities which include vascular aging, cognitive impairment, loss of muscle, osteoporosis, and hypoglycemia.

#### 2.3.1 Vascular aging

Vascular aging refers to arterial functional, structural, and mechanical changes that occur with aging or age-related metabolic diseases within the cardiovascular system (Ryder et al., 2020; Singam et al., 2020). The prominent structural changes in aging vessels include increased arterial stiffness, reduced compliance, diminished vascular repair and regeneration capacity, and impaired endothelial cell function, ultimately leading to the development of atherosclerosis and calcification (Gopcevic et al., 2021). The inflammation, oxidative stress, autophagy, and the accumulation of AGEs are associated with the entire process of vascular aging.

Oxidative stress is currently recognized as the "ultimate common pathway" for many chronic age-related diseases (Pitocco et al., 2013), as it can disrupt cellular metabolism and homeostasis, leading to endothelial cell damage. Reactive oxygen species (ROS) serve as the major drivers of oxidative stress. T2DM leads to excessive ROS production through various pathways. Early research suggested that glucose could directly stimulate excessive ROS production (Du et al., 1999), but later studies found that high glucose (HG) activates various enzymatic cascades in mitochondria (Rizwan et al., 2020), including the activation of NADPH oxidase (Jansen et al., 2013), NO synthase uncoupling (Sasaki et al., 2008), and the stimulation of xanthine oxidase (Hernandez-Hernandez et al., 2022). Similarly, elderly individuals often have comorbidities such as dyslipidemia and hypertension, which can also induce ROS production.

AGEs are non-enzymatically formed through the condensation of the carbonyl group of reducing sugars with free amino groups in nucleic acids, proteins, or lipids. These compounds then undergo further rearrangement, producing stable and irreversible end products that can alter tissue function and mechanical properties (Twarda-Clapa et al., 2022). AGEs are associated with many age-related diseases and accumulate in various tissues, exerting cytotoxic effects. Research has shown that the AGEs/Receptor for Advanced Glycation End Products (RAGE) signaling pathway plays a central role in diabetic-related atherosclerosis and narrowing processes (Soro-Paavonen et al., 2008; Kopytek et al., 2020). Elevated glucose levels in T2DM patients can promote the late glycation end-product and collagen cross-linking, resulting in stiff and less hydrolysable collagen proteins, thereby increasing vascular wall stiffness (Aronson, 2003; Reddy, 2004). AGEs stimulate endothelial cells to produce ROS through RAGE activation, and these signaling molecules can activate the NF-κB and downstream signaling pathways (Dorenkamp et al., 2023; Shu et al., 2023). AGEs also inhibit the phagocytic action of macrophages by binding to AGEs receptors, thus promoting inflammation (Du et al., 2023). Additionally, HG-induced AGEs dysregulate the chromatin remodeling through DNA methyltransferases (DNMTs), DNMT1-ten-eleven translocations (TETs), histone modifications, miRNAs, and lncRNAs. This leads to changes in chromatin structure and persistent vascular damage through metabolic memory, ultimately resulting in a chronic inflammatory state and vascular complications (Dhawan et al., 2022).

Autophagy is a critical regulator of cellular metabolism and intracellular homeostasis, playing a crucial role in maintaining the normal function of vascular cells. Autophagy dysfunction has been observed in some age-related diseases such as T2DM (Sehrawat et al., 2023). Reduced autophagic activity not only leads to the delayed and abnormal accumulation of denatured proteins and dysfunctional organelles but also, through various pathways, results in endothelial dysfunction and intimal thickening, exacerbating vascular aging. Autophagy has been shown to play roles in the homeostasis of β-cells, IR, clearance of protein aggregates such as islet amyloid polypeptide, and various insulin-sensitive tissues (Sehrawat et al., 2023).

Moreover, the activation of the inflammasome via the NOD (nucleotide-binding oligomerization domain)-like receptor family, pyrin domain containing 3 (NLRP3) pathway during T2DM can also promote chronic inflammation and exacerbate vascular endothelial cell aging and endothelial dysfunction (Gora et al., 2021).

#### 2.3.2 Cognitive impairment in elderly diabetes

Cognitive impairment is increasingly recognized as a significant comorbidity of diabetes. Diabetes-related cognitive impairment progresses through different stages. The more severe stages, particularly mild cognitive impairment and dementia, often accompanied by progressive deficits, predominantly manifest in elderly individuals (Biessels and Despa, 2018).

Insulin and Insulin-like Growth Factor receptors (IGF) receptors, akin to insulin-like peptides, are expressed in neurons and glial cells, with insulin receptor (IR), insulin-like growth factor 1 (IGF1R), and insulin-like growth factor 2 (IGF2R) signaling through their respective receptors well-expressed in regions such as the hippocampus, striatum, hypothalamus, cerebral cortex, and olfactory bulb (Duarte et al., 2012). In elderly individuals with diabetes, alterations in insulin levels and/or signaling pathways in the brain occur due to cerebral IR. This results in neuronal loss, disruption of peripheral metabolism, and synaptic dysfunction (Wijesekara et al., 2018). Studies have found that impaired brain insulin-PI3K-AKT signaling may promote neurodegeneration in Alzheimer’s disease (AD) by downregulating O-GlcNAcylation, subsequently promoting abnormal tau hyperphosphorylation and neurofibrillary degeneration (Liu et al., 2011b). Intravenous or intranasal insulin administration has improved memory function in both humans and animals (Chapman et al., 2018; Wu et al., 2023b), indicating that compromised insulin signaling pathways may be a primary defect linking AD and T2D.

Currently, a substantial body of research suggests that AD primarily results from an imbalance between the generation and clearance of Aβ, promoting Aβ accumulation in the central nervous system and triggering AD (Hardy and Selkoe, 2002; Selkoe and Hardy, 2016). Hyperglycemic states contribute to cerebral endothelial damage, promote atherosclerosis, affecting cerebral perfusion and function while impeding the clearance of brain metabolites. This may impair the Aβ clearance system, further promoting Aβ deposition in the brain (Hamzé et al., 2022).

#### 2.3.3 Sarcopenia and osteoporosis in elderly diabetes

Sarcopenia is a progressive, systemic skeletal muscle disorder that is associated with an increased risk of adverse outcomes such as falls, fractures, physical disability, and mortality (Schaap et al., 2018).

In elderly patients with T2DM, the pro-inflammatory pathway is activated during the aging process of skeletal muscle. However, due to a decrease in the activity of antioxidant enzymes, the number of mitochondria decreases, and their anti-oxidative capacity decreases, thereby leading to an increase in intracellular accumulation of reactive oxygen species and oxidative stress levels in skeletal muscle (Crescioli, 2020). Besides, low muscle mass is linked to poor blood glucose control (Alabadi et al., 2023). This appears to be a bidirectional relationship, where prolonged exposure of cells and tissues to high blood glucose levels promotes the accumulation of AGEs in skeletal muscle, leading to increased oxidative stress, mitochondrial dysfunction (Ritov et al., 2010; Tabara et al., 2019), and impaired insulin synthesis and metabolism (Gougeon, 2013). All of these factors contribute to muscle damage and a lack of physical activity, ultimately resulting in the loss of muscle mass and function, referred to as muscle wasting syndrome. Therefore, T2DM is also considered a significant predictive factor for sarcopenia (Cruz-Jentoft et al., 2019). In addition, age-related insulin-mediated impaired glucose uptake is related to the gradual deterioration of skeletal muscle structure and function. In the human body, skeletal muscle accounts for over 80% of glucose uptake following oral glucose load, and insensitivity of this organ can lead to IR and elevated blood glucose levels (Merz and Thurmond, 2020). Muscle mass plays a pivotal role in facilitating glucose disposal mediated by insulin, and its reduction can further exacerbate IR (Maliszewska et al., 2019). Potential mechanisms include mitochondrial dysfunction, increased low-grade inflammation, lipid accumulation, and oxidative stress in intramuscular cells, as well as accumulation and decreased autophagy and enzyme activity in aging cells (Jiao and Demontis, 2017; Kalinkovich and Livshits, 2017; Crescioli, 2020; Shou et al., 2020).

The musculoskeletal system is a comprehensive and interconnected system, and diabetic patients with muscle wasting syndrome are more prone to developing osteoporosis, and lower muscle mass and strength, along with higher fat content, can impair bone quality (Herrmann et al., 2020). Due to the long-term blood glucose fluctuations, elderly T2DM patients may experience metabolic disturbances which are unfavorable for bone matrix (HICKMAN et al., 2018). Furthermore, HG levels can lead to osmotic diuresis, and disturbances in calcium-phosphorus metabolism, causing significant loss of trace elements such as calcium and phosphorus, resulting in decreased bone density, decreased levels of bone growth factors and bone remodeling function (Seyfizadeh et al., 2018; Sinnott-Armstrong et al., 2021). Poor long-term blood glucose control leads to an increase in AGEs which can also lead to abnormalities in bone organic matter metabolism (Zhang et al., 2023).

Overall, the underlying pathophysiological mechanism of bone fragility in diabetes is very complex, including hyperglycemia, oxidative stress, and the accumulation of advanced glycosylation end products, which will damage the characteristics of collagen, increase bone marrow obesity, release inflammatory factors and adipokines from visceral fat, and may change the function of bone cells. Other factors include treatment-induced hypoglycemia, some antidiabetic drugs (such as thiazolidinediones) that have a direct impact on bone and mineral metabolism, and an increased tendency to fall, all of which will lead to an increased risk of fracture in diabetes patients (Napoli et al., 2017).

#### 2.3.4 Hypoglycemia

Clinical research shows that for older patients with diabetes, the result of an intensive hypoglycemic treatment strategy is that the risk of hypoglycemia in patients with T2DM is significantly increased, and the mortality rate with cardiovascular events is increased (Gerstein et al., 2011). As is well known, hypoglycemia is the main cause of myocardial infarction and cardiovascular events, and the regulatory mechanism of hypoglycemia, especially in elderly people, is weakened (Ishikawa et al., 2018). In elderly patients with diabetes, the secretion of incretin is reduced, the storage and release function of glycogen is weakened, the ability of self-regulating hypoglycemia is reduced, the liver and kidney functions are reduced, and multi-drug treatment caused by various chronic comorbidities (including heart disease, stroke, and chronic kidney disease) can increase the risk of severe hypoglycemia (Corsonello et al., 1999; Lipska et al., 2016). The consequences of recurrent hypoglycemic episodes include acute and long-term cognitive changes, arrhythmia and myocardial infarction, severe falls, weakness, and death; For elderly diabetes patients with sympathetic nerve dysfunction, the induction of hypoglycemia is reduced, and asymptomatic hypoglycemia, nonspecific neurological symptoms (improper speech, confusion of thinking, strange behavior) or direct hypoglycemic coma may occur.

## 3 The mechanisms of TCM in preventing and treating elderly diabetes

**FIGURE 2:**
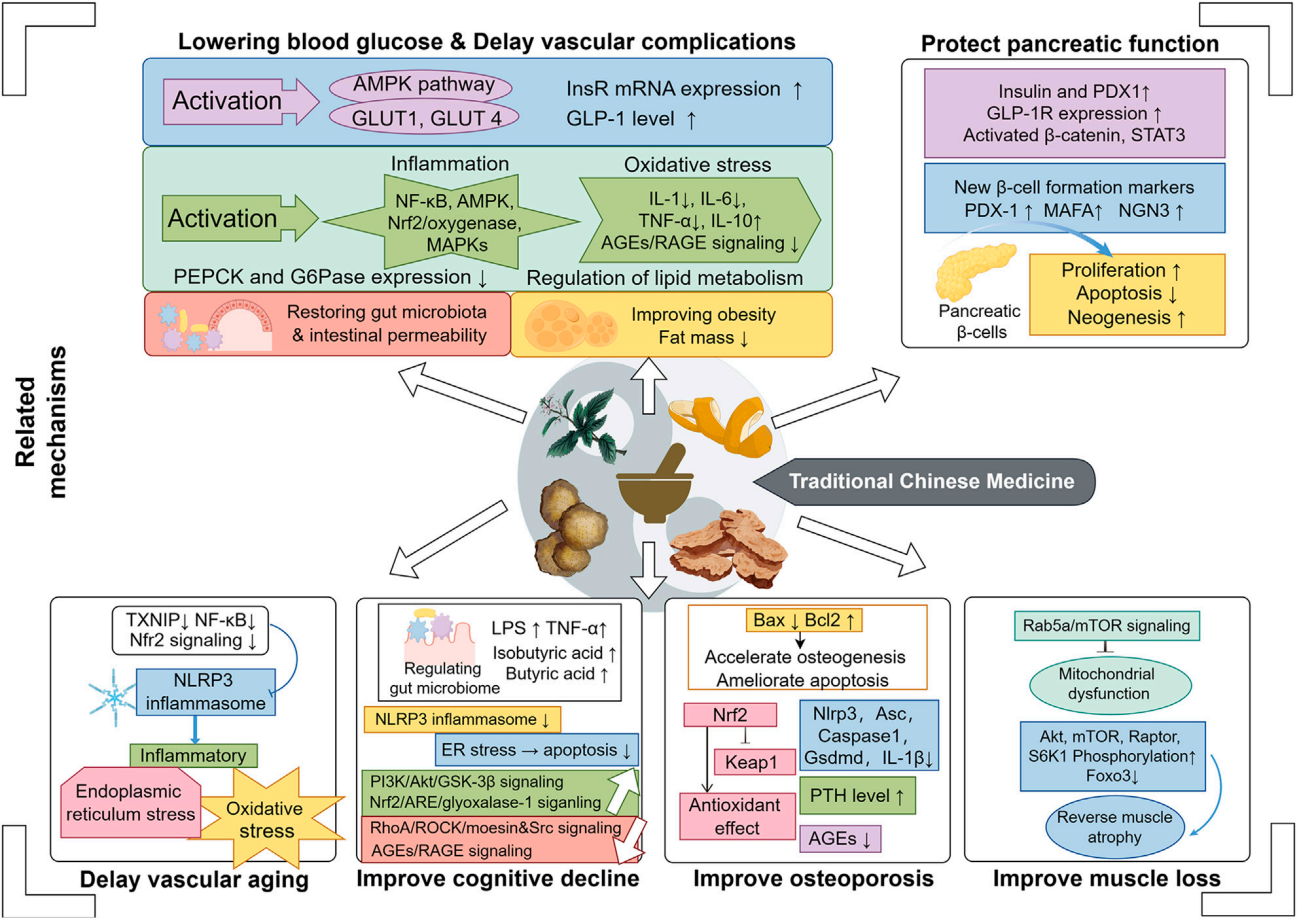
TCM is widely utilized in the treatment of diabetes, its vascular complications, and common comorbidities associated with elderly diabetes. The mechanisms underlying its efficacy have been preliminarily summarized as depicted in the figure below. As illustrated in the figure, traditional Chinese medicine operates through multiple targets and pathways in a synergistic manner to ameliorate diabetes and its related vascular complications and comorbidities. These effects encompass but are not limited to the improvement of oxidative stress, ERS, inflammatory responses, inflammasomes, advanced glycation end products pathways, insulin-related signaling pathways, lipid metabolism pathways, gut microbiota, and bone metabolism, among others.

The prevalence of diabetes among the elderly is high, and overall blood glucose control is suboptimal. Consequently, the rates of disability and mortality due to complications and comorbidities are elevated. TCM plays a significant role in the treatment of elderly diabetes by improving disorders in glucose and lipid metabolism, controlling risk factors, complications, and comorbidities (Figure 2).

### 3.1 The mechanisms of TCM in lowering blood glucose and delaying vascular complications

TCM has shown significant efficacy in lowering blood glucose and managing vascular complications in diabetes. Numerous clinical trials have confirmed the clinical effectiveness of TCM in improving blood glucose levels. The research on the therapeutic mechanisms of TCM has explored a wide range of aspects, including herbal compounds and monomers. For example, berberine, derived from the Chinese botanical drug Huanglian, has become a prominent bioactive compound with potent glucose-lowering effects (Xie et al., 2022). Its therapeutic effect on T2DM was first reported in mice as early as 1986 (Chen and Xie, 1986). Subsequently, extensive research has evaluated the anti-diabetic activity of berberine both *in vitro* and *in vivo* (Han et al., 2021). The anti-diabetic activity of berberine is attributed to its multifaceted mechanisms, including the activation of the AMP-activated protein kinase (AMPK) pathway, activation of GLUT1 and AKT/GLUT4 signaling pathway, enhancement of glucagon-like peptide-1 (GLP-1) levels, upregulation of insulin receptor (InsR) mRNA expression, inhibition of PEPCK and G6Pase expression, suppression of inflammation (IL-1, IL-6, TNF-α, COX-2, and iNOS), and regulation of lipid metabolism, among others (Shrivastava et al., 2023). TCM decoction like Gegen Qinlian Tang (GQD) have demonstrated their ability to improve high blood sugar and protect pancreatic function by modulating the structure of gut microbiota, thereby restoring intestinal permeability and suppressing inflammation in T2DM rats (Tian et al., 2021). Furthermore, TCM has been proven to synergistically ameliorate multiple metabolic disorders. For instance, the TCM Jinlida (JLD) granules enhance mitochondrial biogenesis and fatty acid oxidation, significantly improving obesity, increased fat content, maintaining glucose and lipid homeostasis, and ameliorating hepatic steatosis and inflammation induced by HFD (Zhang et al., 2019). Recent studies also summarize that Chinese botanical drugs have a significant therapeutic potential in improving T2DM by regulating mitochondrial respiratory chain complexes in various cell types (Zhang et al., 2024).

TCM also demonstrates substantial advantages in preventing and treating vascular complications in diabetes, slowing down vascular aging, and delaying renal lesions. The TCM herbal monomer Danshinone IIA (Tan IIA) can alleviate kidney damage in db/db mice, possibly by inhibiting cell pyroptosis through the regulation of NLRP3 and thioredoxin-interacting protein (Txnip) expression, thus delaying the progression of diabetic kidney disease (DKD) (Wu et al., 2023a). Dysregulated autophagy is one of the critical mechanisms underlying microvascular complications in diabetes. Emerging research suggests that TCM and their active compounds can improve diabetic kidney damage by regulating autophagy (Liu et al., 2023b). Based on network pharmacology, molecular docking, and experimental validation, a mixture of Schisandra chinensis fruit improved kidney function and pathological changes in DKD rats, possibly by downregulating the AGEs/RAGE signaling pathway, further downregulating the expression of TNF-α, IL-1β, IL-6, upregulating IL-10, among other mechanisms (Li et al., 2023a).

### 3.2 The mechanisms of TCM in improving the islet function of elderly diabetes patients and diabetes related complications

The experimental study on TCM improving the function of islets in elderly diabetes and diabetes related comorbidities shows that Chinese medicine plays a role in treating elderly diabetes and its complications and comorbidities by regulating the proliferation, apoptosis and differentiation of islet cells, delaying cognitive dysfunction related diseases, regulating bone metabolism and differentiation, and improving muscle loss.

#### 3.2.1 Regulating of pancreatic islet cell proliferation and apoptosis

TCM may treat elderly diabetes by regulating the proliferation and apoptosis of pancreatic islet cells. A TCM formulation known as Shenqi Compound (SQC), composed of Panax Ginseng, Astragali Radix, Rhizoma Dioscoreae, Corni Fructus, Rehmanniae Radix, Salviae Miltiorrhizae Radix et Rhizoma, Radix Trichosanthis, and Rhei Radix et Rhizoma, has been found to significantly control blood glucose levels, inhibit IR, reduce hyperinsulinemia, and protect pancreatic islet hypertrophy. It accomplishes this through alleviating oxidative stress and suppressing inflammation, as well as inhibiting the apoptosis and senescence of β-cells (Yang et al., 2023). Fufang-zhenzhu-tiaozhi formula (FTZ), a patented TCM preparation, has been demonstrated to promote β-cell regeneration by protecting the islets from inflammatory cell invasion, maintaining the number of pancreatic β-cells, and increasing the expression of key markers of new β-cell formation, such as PDX-1, MAFA, and NGN3 (Chen et al., 2023). Research has revealed that Puerarin, an isoflavone derived from the root of Pueraria lobata (Willd.) Ohwi, significantly improves blood glucose stability in high-fat diet-induced diabetic mice by promoting β-cell neogenesis.

Puerarin has demonstrated a significant improvement in blood glucose homeostasis in HFD-induced diabetic mice. Additionally, during the treatment of HFD-fed mice with puerarin, the pancreatic ducts exhibited the presence of markers of new β-cell formation, including insulin, PDX1 (Pancreatic and Duodenal Homeobox 1), and Ngn3 (Neurogenin 3). Moreover, this treatment induced the expression of insulin and PDX1 in the pancreatic ducts, along with the upregulation of GLP-1R expression, followed by the activation of β-catenin proteins and STAT3 (Wang et al., 2020a).

#### 3.2.2 Slowing vascular aging

Cellular aging is a critical factor in the development of elderly diabetes, while vascular aging is a degenerative condition that occurs in the cardiovascular system as one ages. Research has shown that extracts derived from ginseng, sanqi, and chuanxiong may potentially slow down endothelial cell aging induced by high glucose and high fat (Wang et al., 2020b). This is achieved by enhancing cellular autophagy activity, elevating mitochondrial membrane potential, and reducing the accumulation of DNA damage caused by ROS generation (Wang et al., 2020b). Furthermore, another study discovered that these extracts can lower random blood glucose levels in aging diabetic mice, inhibit the expression of proteins related to the AMPK/mTOR pathway, improve cardiac aging in mice, reduce vascular calcification, and delay vascular aging (Hu et al., 2020).

A considerable number of active components derived from TCM have been demonstrated to inhibit the NLRP3 inflammasome. Published data suggest that many candidate drugs from traditional herbal sources exert anti-inflammatory effects by inhibiting upstream signals of NLRP3, including TXNIP and NF-κB, or by combating oxidative stress, such as promoting Nfr2 signal transduction. Ultimately, these interventions may lead to targeted inhibition of the NLRP3 inflammasome, resulting in the amelioration of oxidative stress, endoplasmic reticulum stress (ERS), inflammatory pathways, and the suppression of pro-inflammatory cytokines. This approach holds promise for improving diabetes and its complications (Bai et al., 2021).

#### 3.2.3 Improving diabetes-related cognitive impairment

Extensive research has confirmed the significant therapeutic effects of TCM on diabetes-related cognitive dysfunction (DCD). Most TCM and their active ingredients can ameliorate DCD by reducing IR, microvascular dysfunction, abnormal gut microbiota composition, inflammation, and damage to the blood-brain barrier, cerebral blood vessels, and neurons under hyperglycemic conditions (Meng et al., 2021). Specifically, the underlying mechanisms involve the regulation of various signaling pathways, such as PI3K/Akt/GSK-3β signaling pathways (Guo et al., 2023), RhoA/ROCK/moesin and Src signaling pathways (Li et al., 2018b), AGEs/RAGE (Wang et al., 2012), NLRP3 inflammasome (Tian et al., 2023), ERS (Chen et al., 2018), and Nrf2/ARE (Liu et al., 2019), among others. These pathways collectively improve IR, synaptic plasticity, and exert anti-inflammatory, antioxidant, anti-ERS, and anti-neuronal apoptosis effects.

Recent studies have shown that Danshinone IIA (TAN) lowers fasting blood glucose (FBG) levels and enhances cognitive and memory function in HFD and streptozotocin (STZ)-induced diabetic animals. The potential mechanism may be related to the modulation of the gut microbiota by TAN. TAN regulates neuronal biomarkers, reduces serum levels of LPS and TNF-α, corrects the reduced abundance of specific microbial taxa in diabetic rats, regulates the abundance of specific microbial taxa to control pathways related to fatty acid lipid metabolism and biosynthesis, and significantly restores decreased levels of isobutyric acid and butyric acid (Zheng et al., 2022a). Similar studies have also demonstrated the beneficial effects of dendrobium mixture (consisting of Dendrobii Caulis, Astragali Radix, and Rehmanniae Radix) in alleviating DCD by regulating gut microbiota composition (Zheng et al., 2022b).

#### 3.2.4 Improving diabetic osteoporosis

Diabetic osteoporosis (DOP) is a chronic bone metabolic disorder induced by diabetes, and research has shown that TCM can treat DOP by improving bone metabolism and differentiation. In a recent study, Epimedium brevicornum, mainly composed of Epimedium brevicornum polysaccharides, was found to promote bone formation and ameliorate apoptosis by regulating the Bax/Bcl-2 signaling pathway, thus accelerating osteogenesis in osteoblasts in a HG-induced DOP model (Lei et al., 2023). Arabinoxylans (PPCP-1) isolated from the bark of Phedendron chinense Schneid were shown to downregulate the expression of AGEs receptors induced by streptozotocin in the tibia of diabetic rats, thereby improving diabetes-associated osteoporosis (Wang et al., 2021). Anemarrhenae Rhizoma/Phellodendri Chinensis Cortex (AR/PCC) herbal compound has been shown to effectively lower fasting blood glucose levels in diabetic rats, reverse the osteoporotic phenotype, significantly improve trabecular area percentage, trabecular thickness, and trabecular number in vertebral bodies, and reduce trabecular separation (Xu et al., 2022). Another research has found that the AR/PCC herbal compound improved osteogenesis, promoted neurite outgrowth, and enhanced angiogenesis (Fu et al., 2023) by reducing the overexpression of Nlrp3, Asc, Caspase1, Gsdmd, and IL-1β, thus alleviating abnormal activation of apoptosis in vertebral osteoblasts of diabetic rats (Fu et al., 2023). Additionally, it upregulated the antioxidant response protein Nrf2, activating the antioxidant pathway, while simultaneously reducing its negative feedback regulator Keap1 (Fu et al., 2023). Ligustroflavone, an active compound in Ligustrum lucidum (Scrophulariaceae), has been found elevate parathyroid hormone (PTH) levels in diabetic mice, regulate calcium metabolism, and prevent osteoporosis (Feng et al., 2019). Rehmannia glutinosa (Scrophulariaceae) regulated alkaline phosphatase activity and bone alkaline phosphatase levels in diabetic rats, enhancing bone density and improving bone microstructure. Catalpol (CAT), acteoside (ACT), and echinacoside (ECH) extracted from Rehmannia glutinosa promoted bone formation by regulating the IGF-1/PI3K/mTOR signaling pathway (Gong et al., 2019).

#### 3.2.5 Improving diabetic muscle loss

T2DM in the elderly can lead to a decline in muscle mass and grip strength. Skeletal muscle, as one of the largest organs in the human body, is responsible for up to 80% of postprandial glucose uptake (Merz and Thurmond, 2020). Impairments in skeletal muscle glucose uptake and utilization play a critical role in the development of T2DM. Previous research has demonstrated that the combination of Astragalus membranaceus and Dioscorea opposita improves diabetic muscle atrophy by addressing mitochondrial dysfunction mediated by the Rab5a/mTOR pathway (She et al., 2023). Another herbal combination, AR/PCC reverses muscle atrophy in diabetic mice through the Akt/mTOR/FoxO3 signaling pathway (Zhang et al., 2014b).

## 4 The systematic review of TCM’s clinical application for elderly diabetes

### 4.1 Method

#### 4.1.1 Search strategy and study selection

Relevant studies were identified by searching for papers published from January 2000 to November 2023 in the following databases: Web of Science, Pubmed, Embase, Cochrane Library, Sinomed, China National Knowledge Internet, Wanfang and VIP. Search terms included the following: (“diabetes” or “diabetes mellitus” or “diabetes nephropathy” or“diabetes retinopathy” or “diabetes peripheral neuropathy” or “diabetic cardiomyopathy” or “diabetic gastroparesis” or “diabetic foot” or “diabetes and osteoporosis” or “diabetes and sarcopenia” or “diabetes and coronary heart disease” or “diabetes and arteriosclerosis” or “diabetes and cognitive impairment”) and (older or elderly or senile) and (“randomized controlled trial” or “controlled clinical trial” or “random” or “randomly” or “randomized” or “control” or “RCT”) and (“TCM” or “traditional Chinese medicine” or “Chinese medicinal herb” or “Chinese herbal medicine” or “decoction” or “formula” or “prescription” or “powder” or “Chinese patent medicine” or “Chinese patent drug” or “Chinese herbal compound prescription” or “granule” or “pill” or “tablet” or “capsule” or “admixture” or “Chinese medicine extract” or “extractive” or “glycosides” or “polysaccharide” or “oil”). The authors of the identified papers were contacted for additional information if necessary.

#### 4.1.2 Inclusion and exclusion criteria

We included clinical studies that satisfied the following criteria: (a) Study participants were diagnosed with elderly diabetes, with or without diabetes nephropathy, diabetes peripheral neuropathy, diabetes retinopathy, diabetes cardiomyopathy, diabetes gastroparesis, diabetes foot, cognitive impairment, osteoporosis, sarcopenia, coronary heart disease, and arterial sclerosis. (b) Sample size ≥60; (c) The study follow-up ≥12 weeks.

We excluded clinical studies with the following features: (a) Studies that were non-randomized; (b) Patients that were enrolled with no definite. (c) Sample size <60; (d) The study follow-up <12 weeks; (e) Non-oral Chinese medicine treatment; (f) TCM treatment based on syndrome differentiation, the therapeutic drugs are uncertain; (g) The control group was not a western drug or the placebo; (h) studies that reported only symptomatic changes in patients without objective laboratory measurements or physical examination; (i) Conference papers or dissertations; (j) Full text not found.

#### 4.1.3 Study selection and data extraction

According to the above design, two reviewers (Qiqi Zhang and Shiwan Hu) searched the online databases listed above and assessed the eligibility of these articles and made decisions on every research (inclusion or exclusion) independently. If they did not reach the same decision, the concerned articles were discussed with a third reviewer (Zishan Jin). Three reviewers (Qiqi Zhang, Shiwan Hu and Zishan Jin) extracted data independently from each study. Differences of extracted data were solved after discussion with a fourth reviewer (Boxun Zhang).

#### 4.1.4 Data statistics

All the studies were divided into three subgroups of Traditional Chinese Prescription, Traditional Chinese patent medicines and Traditional Chinese Medicine Extracts for analysis. If there were ≥5 studies included, the frequency of using TCM will be statistically analyzed. For adverse reactions, the frequency and number of symptoms in the control group and intervention group were separately counted. The above analyses were conducted in Microsoft Excel.

#### 4.1.5 Quality assessment and ConPhyMP statement

Quality assessment of all the trials included in this review was independently evaluated by three reviewers (Qiqi Zhang, Shiwan Hu and Zishan Jin) using Jadad Scale (Jadad et al., 1996). Any disagreement was resolved by discussions with a fourth reviewer (Boxun Zhang).

Two researchers (Qiqi Zhang and Shiwan Hu) evaluated all studies on traditional Chinese medicine extracts using the guidelines outlined in the ConPhyMp statement (Heinrich et al., 2022). If there is any dispute, it shall be determined by the third researcher (Zishan Jin).

### 4.2 Result

#### 4.2.1 Study inclusion

**FIGURE 3:**
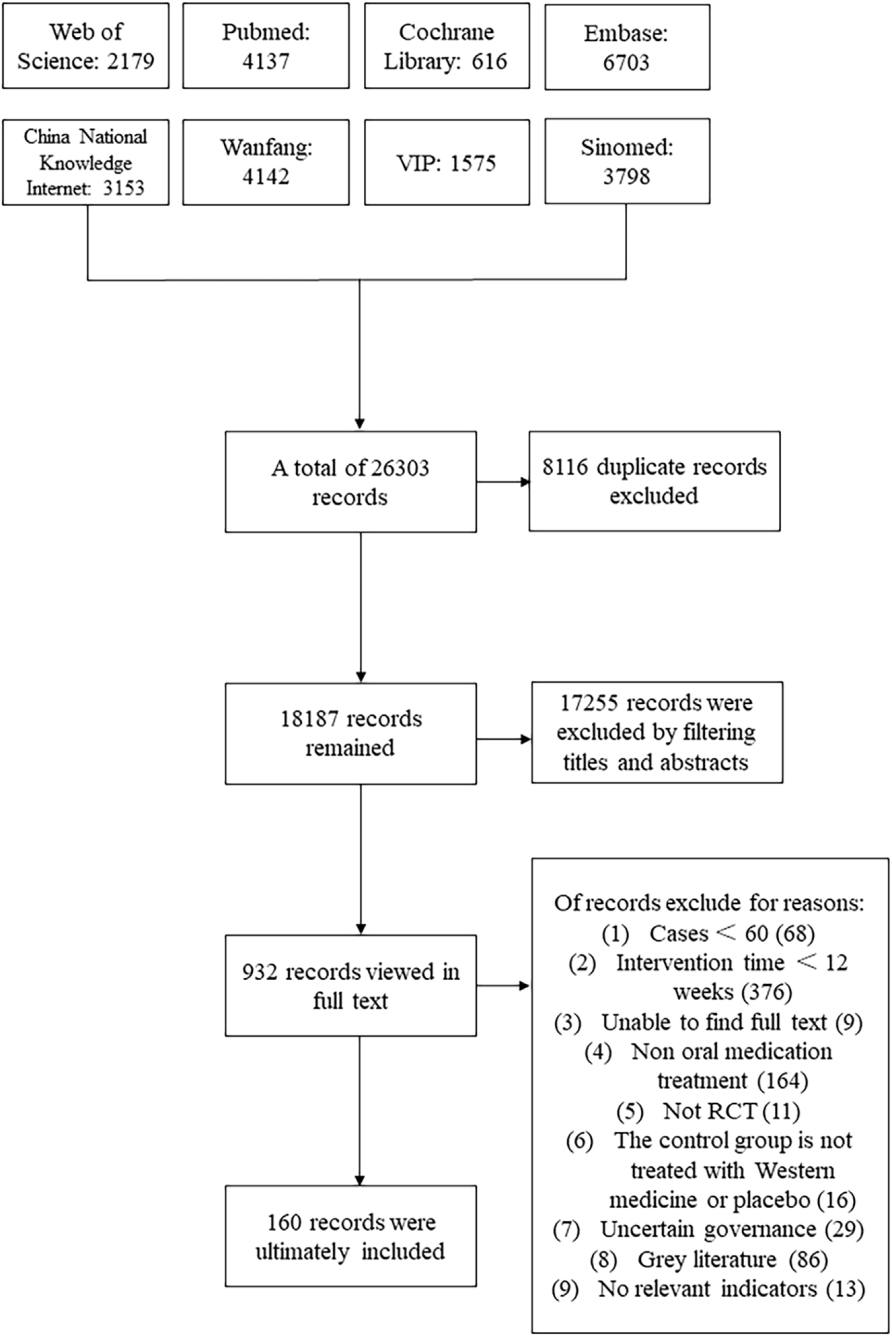
Flowchart of database searching and study identification.

We searched 26,303 articles from eight databases, and after deleting duplicates, the number of articles was reduced to 18,187. According to the title and abstract of the articles, we excluded 17,254 articles for reasons including animal experiments, case reports or reviews, and not related to TCM treatment of elderly diabetes. Subsequently, we downloaded the full text of the remaining 933 articles for further screening, and according to the inclusion and exclusion rules, we finally included 160 articles. Articles on elderly diabetic gastroparesis were excluded because they were all followed up for less than 12 weeks. An article on elderly diabetes with sarcopenia was also excluded due to its study size <60 participants. The flow chart of the study selection process is shown in Figure 3.

#### 4.2.2 Study characteristics

All the included 160 studies were conducted in China, of which 159 were published in Chinese and 1 in English. The control group in 159 trials were oral western medicine or insulin injection therapy, and the control group in 1 trial was placebo.

#### 4.2.3 TCM for elderly diabetes mellitus and islet function

##### 4.2.3.1 Traditional Chinese Prescription

A total of 28 RCTS, involving 2737 subjects, were conducted on Traditional Chinese Prescription. The age range of the included subjects was between 50 and 93 years, and the duration of the intervention ranged from 12 weeks to 4 months (Supplementary Table S1; Supplementary Table S2). (Xue et al., 2010; Li and Chen, 2011; Fu et al., 2013; Wang et al., 2013; Zhou and He, 2013; Zhu and Li, 2013; Zhou et al., 2014b; Li et al., 2014; Wu and Zheng, 2015; Liu, 2016; Zhao, 2016; Ma, 2017b; Zou, 2017; Ailiyasi and LAI, 2019; Su, 2020b; Dai, 2020; Ding, 2020; Xu et al., 2020; Ni et al., 2021; Wei et al., 2021; Zhu, 2021; Wang, 2022c; Ma and Li, 2022; Sun et al., 2022; Zha, 2022; Zhao and Zhi, 2022; Han, 2023; Jiang, 2023). There were 22 kinds of Traditional Chinese Prescription in the intervention group, of which the most commonly used were Gegen Qinlian Decoction (3 RCTs), Liuwei Dihuang Decoction (2 RCTs), Sanhuang Decoction (2 RCTs), Jiangtangjing Granules (2 RCTs).

The most commonly used drugs included *Astragalus mongholicus Bunge* [Fabaceae, Astragali radix] (20 times), *Dioscorea oppositifolia L.* [Dioscoreaceae, Dioscoreae rhizoma] (18 times), *Pueraria montana* var. *lobata (Willd.) Maesen & S.M.Almeida ex Sanjappa & Predeep* [Fabaceae, Puerariae lobatae radix] (13 times), *Coptis chinensis Franch.* [Ranunculaceae, Coptidis rhizoma] (12 times), *Rehmannia glutinosa (Gaertn.) DC.* [Orobanchaceae, Rehmanniae Radix] (12 times) (Supplementary Table S3a).

Ni et al. (2021); Sun et al. (2022); Jiang (2023) all conducted experiments using Gegen Qinlian Decoction, and the three studies all targeted elderly diabetic patients with gastrointestinal dampness and heat syndrome. However, the composition and dosage of Gegen Qinlian decoction in Jiang’s study were different from those in the other two trials. Jiang et al. found that Gegen Qinlian Decoction could improve the blood glucose level and islet function of the subjects. Ni et al. and Sun also found that Gegen Qinlian Decoction was beneficial to reduce the lipid metabolism indicators of the subjects.

Zhu (2021); Ma and Li (2022); Zhao and Zhi (2022) all used Liuwei Dihuang Decoction combined with western medicine for the treatment of elderly diabetes, but the composition and dosage of the prescription used in the three studies were different. The results showed that in addition to reducing the blood sugar level of the subjects, the Liuwei Dihuang Decoction used by Zhu could also improve the level of serum inflammatory factors in the patients, and the prescription used by Zhao et al. could also improve the blood lipid index of the patients. Two studies using Sanhuang Decoction in elderly patients with diabetes showed that it can improved blood glucose level, serum inflammatory factors and oxidative stress, as well as HOMA-IR, TC and TG levels (Ailiyasi and LAI, 2019; Wei et al., 2021). Xue et al. (2010); Zhu and Li (2013) used Jiangtangjing Granules and found that compared with conventional western medicine treatment, Jiangtangjing Granules could reduce the indicators of glucose and lipid, regulate the coagulation function of patients, and improve their prethrombotic state.

Of the 28 RCTs, nine studies evaluated measures of islet function, including Gegen Qinlian Decoction, Erban Decoction, self-designed Yangyin Xiaoke Recipe, Sanhuang Decoction, Jingui Shenqi Prescription, Modified Taohe Chengqi Decoction and Yuye Decoction. The results all proved that Traditional Chinese Prescriptions have positive effect on improving islet function in elderly diabetic patients.

##### 4.2.3.2 Traditional Chinese patent medicines

A total of 25 RCTs involving 2338 subjects explored the application of Traditional Chinese patent medicines in elderly diabetes. The age range of the included population ranged from 55 to 89 years, and the intervention period was 12–30 weeks (Supplementary Tables S1; Supplementary Table S2). (Zhang, 2003b; a; Dai and Ou, 2005; Yu et al., 2007; Liu, 2008; Niu and Fang, 2008; Wang and Zhao, 2012; Wu et al., 2012; Hu et al., 2014; Deng et al., 2015; Xia, 2016; Zhao and Yao, 2016; Zhong et al., 2017; Liu and Kang, 2018; Xiao and Zheng, 2018; Bao and Fang, 2019; Hou et al., 2019; Cheng et al., 2020; Jiang et al., 2020; Wu and Shi, 2020; Xiao et al., 2021; Zhao et al., 2021; Li et al., 2022; Han et al., 2023; Li et al., 2023c) The included studies involved a total of 14 Traditional Chinese patent medicines, including Jinqi Jiangtang Tablets (4 RCTs), Jinlida Granules (3 RCTs), Liuwei Dihuang Pills (3 RCTs), Xiaoke Pills (3 RCTs), Shenqi Jiangtang Granules (3 RCTs), Danzhi Jiangtang Capsules (2 RCTs), Yuquan Pills (1 RCT), Shiwei Yuquan Tablets (1 RCT), Jinkui Shenqi Pills (1 RCT), Shenqi Jiangtang Tablets (1 RCT), Xuefu Zhuyu Pills (1 RCT), Maiwei Dihuang Pills (1 RCT), Xuezhikang Capsules (1 RCT), Qiju Dihuang Pills (1 RCT). Among them, Yuquan Pills, Shiwei Yuquan Tablets, Danzhi Jiangtang capsule and Jinkui Shenqi Pills were not included in Pharmacopoeia of the People’s Republic of China 2020 (Committee, 2020). In addition, Shenqi Jiangtang Granules is not included in the Pharmacopoeia, but its Tablets -- "Shenqi Jiangtang Tablets" has been included.

In these Traditional Chinese patent medicines, commonly used drugs included *Rehmannia glutinosa (Gaertn.) DC.* [Orobanchaceae, Rehmanniae Radix] (16 times), *Alisma plantago-aquatica subsp. orientale (Sam.) Sam.* [Alismataceae, Alismatis rhizoma] (12 times), *Dioscorea oppositifolia L.* [Dioscoreaceae, Dioscoreae rhizoma] (12 times), *Astragalus mongholicus Bunge* [Fabaceae, Astragali radix] (11 times), *Poria cocos (Schw.) Wolf Poria* [Polyporaceae, Poria] (11 times) (Supplementary Table S3b). Xiaoke Pills contain glibenuride 0.25 g. Wang and Zhao (2012); Zhong et al. (2017); Xiao and Zheng (2018) explored the efficacy of Xiaoke pills in the treatment of elderly diabetes mellitus. The results showed that Xiaoke pills can reduce the levels of FBG, 2hPBG and HbA1c, and the incidence of hypoglycemia is lower than control group.

Of the 25 RCTs, 10 studies evaluated indicators of islet function, including Danzhi Jiangtang Capsules, Jinlida Granules, Liuwei Dihuang Pills, Maiwei Dihuang Pills, Shenqi Jiangtang Tablets, Shiwei Yuquan Tablets and Yuquan Pills. All studies showed that they could improve the islet function.

##### 4.2.3.3 Traditional Chinese Medicine Extracts

Only one study used Traditional Chinese Medicine Extracts in elderly patients with diabetes. Cheng et al. (2023) applied *Zea mays L.* [Poaceae, corn silk] to elderly patients with newly diagnosed T2DM. Compared with placebo, it was found to reduce FBG and insulin resistance, regulate serum cholesterol levels, and enhance endogenous antioxidant capacity, with no adverse effects on liver and kidney function. Some studies have found that corn silk aqueous extract can inhibit advanced glycation end products (AGEs), and has positive effects on anti-diabetes and anti-aging. However, the pharmacological effects of corn silk against diabetes have been mostly verified *in vivo* or *in vitro* experimental models, and more studies on elderly diabetic patients have not been carried out (Supplementary Table S1; Supplementary Table S2). (Farsi et al., 2008)

#### 4.2.4 TCM for elderly DKD

##### 4.2.4.1 Traditional Chinese prescription

There are 21 RCTs to explore the effect of Traditional Chinese Prescription in elderly DKD, involving a total of 1987 subjects. The age range of the included population ranged from 40 to 80 years, and the intervention duration was 12 weeks– to 24 months (Supplementary Table S4; Supplementary Table S5). (Wen et al., 2006; Gao et al., 2010; Ou et al., 2011; Chen, 2015; Feng et al., 2015; Mi et al., 2015; Zhao et al., 2016b; Chen et al., 2016; Li et al., 2018a; HU and ZHANG, 2018; Jiang et al., 2019; Jin, 2019; Li, 2019; Su et al., 2019; Yang, 2021a; Zhang et al., 2021; Wang, 2022a; Lin, 2022; Zhang et al., 2022; Li et al., 2023b; Feng et al., 2023) Among the 21 studies, three RCTs explored the preventive effect of Traditional Chinese Prescription on DKD in the elderly. After 24 months of follow-up, it was found that compared with the control group, Yiqi Guben Decoction reduced the incidence of DKD in the elderly (Su et al., 2019; Wang, 2022a; Lin, 2022).

Of the other 18 RCTs, 10 studies defined the stage of DKD, and they were mostly applied to stage Ⅲ or Ⅳ. In these studies, a total of 18 prescriptions were involved, and the most commonly used drugs included *Astragalus mongholicus Bunge* [Fabaceae, Astragali radix] (17 times), *Dioscorea oppositifolia L.* [Dioscoreaceae, Dioscoreae rhizoma] (10 times), *Poria cocos (Schw.) Wolf Poria* [Polyporaceae, Poria] (10 times), *Atractylodes macrocephala Koidz.* [Asteraceae, Atractylodis macrocephalae rhizoma] (9 times), *Cornus officinalis Siebold & Zucc.* [Cornaceae, Corni fructus] (9 times), *Salvia miltiorrhiza Bunge* [Lamiaceae, Salviae miltiorrhizae radix et rhizoma] (9 times) (Supplementary Table S6a).

Both (Zhao et al., 2016b; Li et al., 2018) used Huangqi Guizhiwuwu Decoction to treat DKD in the elderly, but the dosage of them were different. Zhao et al. found that Huangqi Guizhiwuwu decoction can improve blood glucose and kidney function index. Li et al. found that Huangqi Guizhi Wuwu Decoction could reduce the expression of TGF-β gene while improving UAER. Jiang et al. (2019); Li (2019) used Yiqi Yangyin Decoction to observe its efficacy in elderly diabetes, but the composition and dosage were different. Both studies found that Yiqi Yangyin Decoction could improve renal function and reduce the level of inflammation in the body. The prescription Jiang et al. used was also able to lower ET levels, which plays an important role in modulation of glomerular filtration rate and renal blood flow, control of renin release, and regulation of transport of sodium, water, protons, and bicarbonate (Kohan et al., 2011).

##### 4.2.4.2 Traditional Chinese patent medicines

There were 37 RCTs to explore the application of Traditional Chinese patent medicines, involving a total of 3447 subjects. The age range of the included population ranged from 50 to 94 years old and the intervention period was 12 weeks to 6 months (Supplementary Table S4; Supplementary Table S5). (Wang and Su, 2007; Bai et al., 2008; Yi et al., 2009; Hong, 2010; Huang et al., 2010; Shu et al., 2010; Sun et al., 2012a; Sun et al., 2012b; Peng and Guo, 2013; Shen et al., 2013; Zhang et al., 2014a; Huang, 2014; Zhu, 2014; Hu et al., 2016; Li and Wang, 2016; Pan and Shang, 2016; Wang et al., 2016; Xie et al., 2016; Yang and Liu, 2016; Ma, 2017a; Chen, 2018; Hu, 2018; Wang, 2018; Wang and Cao, 2018; Fang et al., 2019; Lin et al., 2019; Shi et al., 2019; Su, 2020a; Li and Zhou, 2020; Zhong et al., 2020; Guo, 2021; Shen et al., 2021; Yu et al., 2021; Wang, 2022b; Xu and Wang, 2022; Liu et al., 2023a; Wang et al., 2023) The studies involved a total of 13 Traditional Chinese patent medicines, including Bailing Capsules (10 RCTs), Compound Danshen Dripping Pills (7 RCTs), Shenyan Kangfu Tablets (4 RCTs), Yishen Huashi Granules (4 RCTs), Jinshuibao Capsules (4 RCTs), Huangkui Capsules (2 RCTs), Bailing Tablets (1 RCTs), Congrong Yishen Granules (1 RCT), Jinlida Granules (1 RCT), Jinshuibao Tablets (1 RCT), Niaoduqing Granules (1 RCT), Qi-Kui Granules (1 RCT), Shen’an Capsules (1 RCT). Bailing Tablets, Qi-Kui Granules, Shen’an Capsules, Niaoduqing Granules, Huangkui Capsules were not included by Pharmacopoeia of the People’s Republic of China 2020 (Committee, 2020). Jinshuibao Capsules and Jinshuibao Tablets are both included in Chinese Pharmacopoeia, but they contain different doses of fermented cordyceps sinensis powder. Commonly used medicines include *Cordyceps sinensis (BerK.) Sacc.* [Clavicipitaceae, Cordyceps] (16 times), *Salvia miltiorrhiza Bunge* [Lamiaceae, Salviae miltiorrhizae radix et rhizoma] (12 times), *Alisma plantago-aquatica subsp. orientale (Sam.) Sam.* [Alismataceae, Alismatis rhizoma] (9 times), *Panax ginseng C.A.Mey.* [Araliaceae, Ginseng radix et rhizoma] (9 times), *Poria cocos (Schw.) Wolf Poria* [Polyporaceae, Poria] (8 times) (Supplementary Table S6b).

Of the 37 studies, 22 RCTs defined the stage of DKD in the enrolled population. 16 of these studies were applied to patients with stage 3 and below DKD. The positive effects of Jinshuibao Tablets, Niaoduqing Granules, Bailing Capsules, Jinshuibao Capsules, Jinshuibao Capsules and Shenyan Kangfu Tablets on stage 3–5 elderly DKD have been verified.

##### 4.2.4.3 Traditional Chinese Medicine Extracts

There was only one study on the effect of Traditional Chinese Medicine Extracts on elderly DKD (Supplementary Table S4; Supplementary Table S5). Chen et al. (2022) applied Haikun Shenxi Capsule to early elderly patients with DKD and found it could not only improve the kidney function, but also reduce the inflammatory response and the expression of TGF-β1 and MMP-2. Haikun Shenxi Capsule is composed of Fucoidan, which can protect kidney by improving kidney inflammation, anti-oxidation and anti-fibrosis. In addition, Fucoidan can also counteract renal aging by inhibiting the activity of AMPK-ULK1 signaling pathway. (Zahan et al., 2022).

#### 4.2.5 TCM for elderly DR

##### 4.2.5.1 Traditional Chinese Prescription

Three studies explored the role of Traditional Chinese Prescription in elderly DR, involving 276 participants. The age range of the included population ranged from 58 to 78 years and the intervention period was 3 months–5 months (Table 1; Supplementary Table S7). (Liu et al., 2011a; Wei and Gao, 2012; Li, 2022) The three studies involved two Traditional Chinese Prescription, including Danhuang Mingmu Decoction and Zhenwu Decoction.

**TABLE 1:** TCM for elderly DR.

Study	Subjects (Age range)	Ages^a^	No. of intervention group/control group	Treatment of intervention group^b^	Dose of intervention group	Treatment of control group	Dose of control group	Duration	Outcomes^c^	Adverse reactions
Traditional Chinese Prescription										
Li et al., 2022 (2)	Elderly DR	control group: 62.97 ± 5.28 years old; intervention group: 63.26 ± 6.01 years old	39/39	Danhuang Mingmu Decoction + Calcium Dobesilate	1 dose, 2/d	Calcium Dobesilate	0.5 g, 3/d	5 months	vision, MD, Macular thickness, Bleeding spot area, Hemangioma volume, FBG, 2hPBG, IGF-1, VEGF	control group: loss of appetite (1), vomit (2); intervention group: loss of appetite (1), vomit (1)
Wei and Gao (2012)	Elderly DR (stage Ⅰ-Ⅲ, 58–72 years old)	69.3 ± 5.8 years old	40/40	Zhenwu Decoction + Conventional Western Medicine Treatment	100 mL, 2/d	Conventional Western Medicine Treatment	NM	3 months	vision, ET	NM
Liu et al. (2011a)	Elderly DR (stage Ⅰ-Ⅲ, 64–78 years old)	72.6 years old	60/60	Zhenwu Decoction + Conventional Western Medicine Treatment	100 mL, 2/d	Conventional Western Medicine Treatment	NM	3 months	vision, ET	NM
Traditional Chinese patent medicines										
Wang and Du (2020)	Elderly DR (stage Ⅰ-Ⅲ, 60–83 years old)	control group: 68.35 ± 6.82 years old; intervention group: 69.52 ± 7.11 years old	44/42	Compound Xueshuantong Capsules^†^+Calcium Dobesilate	1.5 g, 3/d	Calcium Dobesilate	0.5 g, 3/d	5 months	vision, MD, Macular thickness, Bleeding spot area, Hemangioma volume, WBV, PV, FIB	control group: loss of appetite (2), gastrointestinal discomfort (2); intervention group: nausea (1), gastrointestinal discomfort (1), loss of appetite (1)
Yan and Yuan. (2014)	Elderly DR (stage Ⅰ-Ⅲ)	control group: 68.8 years old; intervention group: 65.5 years old	40/60	Compound Danshen Dropping Pills^†^+Calcium Dobesilate	10 pills, 3/d	Conventional Western Medicine Treatment	NM	6 months	vision, MD, number of hemangioma, bleeding spot area	NM
Zhang and Zhang (2012)	Elderly DR (stage Ⅰ-Ⅲ, 64–78 years old)	72.6 years old	30/30	Qiju Dihuang Pills^†^+Iodizedlecithin + Adenosine triphosphate + Vitamin tablets	8 pills, 3/d	Iodizedlecithin + Adenosine triphosphate + Vitamin tablets	Iodizedlecithin: 3mg, 2/d; Adenosine triphosphate: 40mg, 2/d; Vitamin tablets: 1–2 tablets, 1/d	3 months	vision, ET	NM

^a^ Ages were displayed as mean ± standard deviation or mean.

^b^ “^†^” indicated that it was included in Pharmacopoeia of the People’s Republic of China 2020.

^c^ “*” showed no significant difference between the intervention group and the control group.

Abbreviation: NM, not mentioned; FBG, fasting blood glucose; 2hPBG, 2-h Postprandial blood glucose; TGF-1, transforming growth factor-1; VEGF, vascular endothelial growth factor; ET, endothelin; WBV, whole blood viscosity; PV, plasma viscosity; FIB, fibrinogen; MD, mean defect.

##### 4.2.5.2 Traditional Chinese patent medicines

Three studies explored the use of Traditional Chinese patent medicines in elderly DR, including 246 participants. The age range of the included subjects ranged from 60 to 83 years old, and the intervention time was 3 months–6 months (Table 1; Supplementary Table S7). (Zhang and Zhang, 2012; Yan and Yuan, 2014; Wang and Du, 2020) The Traditional Chinese patent medicines involved in the three studies included Compound Xueshuantong Capsules, Compound Danshen Dripping Pills and Qiju Dihuang Pills, all of which were included in Pharmacopoeia of the People’s Republic of China 2020 (Committee, 2020).

#### 4.2.6 TCM for elderly DPN

##### 4.2.6.1 Traditional Chinese Prescription

Five studies explored the use of Traditional Chinese Prescription in elderly DPN, involving a total of 446 participants. The age range of the included population ranged from 60 to 80 years, and the intervention period was 12 weeks to 3 months (Table 2; Supplementary Table S8). (Liu et al., 2012; Li et al., 2016b; Guo, 2016; Xu and Zhou, 2017; Yang and Xing, 2019) The prescriptions used in these studies vary, and commonly used Chinese medicines include *Astragalus mongholicus Bunge* [Fabaceae, Astragali radix] (4 times), *Spatholobus suberectus Dunn* [Fabaceae, Spatholobi caulis] (4 times), *Achyranthes bidentata Blume* [Amaranthaceae, Achyranthis bidentatae radix] (3 times), *Angelica sinensis (Oliv.) Diels* [Apiaceae, Angelicae sinensis radix] (3 times), *Carthamus tinctorius L.* [Asteraceae, Carthami flos] (3 times), *Paeonia lactiflora Pall.* [Paeoniaceae, Paeoniae radix rubra] (3 times), *Prunus persica (L.) Batsch* [Rosaceae, Persicae semen] (3 times) (Supplementary Table S9).

**TABLE 2:** TCM for elderly DPN.

Study	Subjects (Age range)	Ages^a^	No. of intervention group/control group	Treatment of intervention group	Dose of intervention group	Treatment of control group	Dose of control group	Duration	Outcomes^b^	Adverse reactions
Traditional Chinese Prescription										
Yang and Xing (2019)	Elderly DPN of qi deficiency and blood stasis syndrome (65–76 years old)	control group: 68.59 ± 2.12 years old; intervention group: 67.91 ± 2.18 years old	38/38	Modified Buyang Huanwu Decoction + Mecobalamin Injection	250 mL, 2/d	Mecobalamin Injection	0.5–1.0 mg, 1/d	3 months	FBG, 2hPBG, HbA1c, SF, SOD, GSH-Px	control group: headache (1, gastrointestinal discomfort (2), rash (3); intervention group: headache (1, gastrointestinal discomfort (4), rash (3)
Xu and Zhou (2017)	Elderly DPN (60–80 years old)	control group: 66. 17 ± 8. 10 years old; intervention group: 65. 08 ± 9. 79 years old	40/40	Yangyinhuoxue Decoction + Mecobalamin	1 dose, 2/d	Mecobalamin Tablets	0.5 mg, 3/d	3 months	SNCV, MNCV	no adverse reaction
Li et al. (2016b) (2)	Elderly DPN (60–72 years old)	control group: 68.68 ± 3.96 years old; intervention group: 62.19 ± 2.93 years old	45/45	Modified Huangqi Guizhi Wuwu Decoction + Mecobalamin	1 dose, 2/d	Mecobalamin Tablets	0.5 mg, 3/d	3 months	FBG, 2hPBG, HbA1c, TC, TG, LDL, HDL, MNCV, SNCV	NM
Guo (2016)	Elderly DPN (61–78 years old)	control group: 69.78 ± 5.96 years old; intervention group: 69.45 ± 5.06 years old	51/51	Compound Qiteng Tongluo Decoction + Epalrestat	1 dose, 2/d	Epalrestat	50 mg, 3/d	12 weeks	EAI, PV, WBV, SNCV, MNCV, FBG, 2hPBG, HbA1c	NM
Li et al., 2012	Elderly DPN	control group: 61.1 ± 65.3 years old; intervention group: 60.3 ± 64.7 years old	49/49	Yiqi Huoxue Tongluo Recipe + Mecobalamin Injection + Vitamin B1	250 mL, 2/d	Mecobalamin Injection + VITAMIN B1 TABLETS	Mecobalamin Injection: 0.5mg, 1/d; VITAMIN B1 TABLETS: 10 mg, 3/d	12 weeks	FBG, MNCV^*^, SNCV^*^	NM
Traditional Chinese Medicine Extracts										
Wang and Zhang (2009)	Elderly DPN (60–80 years old)	control group: 67.5 ± 3.5 years old; intervention group: 66.3 ± 4.9 years old	34/34	Berberine + Mecobalamin Tablets	700 mg, 3/d	Mecobalamin Tablets	0.5 mg, 2/d	12 weeks	FBG, HbA1c, 24h-UTP, MNCV^*^, SNCV^*^	NM

^a^ Ages were displayed as mean ± standard deviation or mean.

^b^ “*” showed no significant difference between the intervention group and the control group.

Abbreviation: NM, not mentioned; FBG, fasting blood glucose; 2hPBG, 2-h postprandial blood glucose; HbA1c, glycosylated hemoglobin; SF, serum ferritin; SOD, superoxide dismutase; GSH-Px, Glutathione peroxidase; SNCV, sensory nerve conduction velocity; MNCV, motor nerve conduction velocity; TC, cholesterol; TG, triglyceride; LDL, low density lipoprotein; HDL, high density lipoprotein; EAI, red cell aggregation index; PV, plasma viscosity; WBV, whole blood viscosity; 24hUTP, 24-h urinary protein quantity.

##### 4.2.6.2 Traditional Chinese Medicine Extracts

One RCT evaluated the role of berberine in elderly DPN, involving 68 participants. The age range of the included population ranged from 60 to 80 years, and the intervention period was 12 weeks (Table 2; Supplementary Table S8) (Wang and Zhang, 2009). After berberine intervention, not only can reduce FBG, HbA1c and 24h-UTP, but also increase the levels of MNCV and SNCV in elderly diabetic patients. Berberine is an effective component of *Coptis chinensis Franch.* [Ranunculaceae, Coptidis rhizoma]. Studies have found that it may play a role in protecting neurons through various signaling pathways such as Pl3K/Akt/Bcl-2 pathway, Nrf2/HO-1 pathway and MAPK signaling pathway. (Lin and Zhang, 2018).

#### 4.2.7 TCM for elderly diabetic Cardiomyopathies (DCM)

Two studies explored the effect of TCM on DCM in the elderly and included studies of Traditional Chinese Prescription only. A total of 146 participants aged between 60 and 80 years were included in the study. The intervention period was 12 weeks (Table 3; Supplementary Table S10). Xing et al. (2023) applied Yangyin Yiqi Huoxue Recipe to treat elderly DCM complicated with heart failure and found that Yangyin Yiqi Huoxue Recipe could improve heart function while lowering blood sugar, and its mechanism might be related to regulating VEGF expression. Chen (2021) treated the elderly diabetic patients complicated with heart arrhythmia with Zhigancao Decoction and found that it could improve the cardiac autonomic nerve function and reduce the level of inflammation.

**TABLE 3:** TCM for elderly DCM.

Study	Subjects (Age range)	Ages^a^	No. of intervention group/control group	Treatment of intervention group	Dose of intervention group	Treatment of control group	Dose of control group	Duration	Outcomes	Adverse reactions
Traditional Chinese Prescription										
Xing et al. (2023)	Elderly diabetes cardiomyopathy with heart failure (60–78 years old)	control group: 67.0 ± 4.3 years old; intervention group: 67.23 ± 3.58 years old	30/30	Yangyin Yiqi Huoxue Recipe + Conventional Western Medicine Treatment	150mL, 2/d	Conventional Western Medicine Treatment	NM	12 weeks	FBG, 2hPBG, HbA1c, VEGF, Cardiac function	no adverse reaction
Chen (2021)	Elderly T2DM with arrhythmia (50–78 years old)	control group: 64.26 ± 8.76 years old; intervention group: 64.32 ± 8.80 years old	43/43	Zhigancao Decoction + Carvedilol Tablets	150 mL, 2/d	Carvedilol Tablets	10–20 mg, 1/d	12 weeks	SBP, DBP, HR, HRV, QTd, hs-CRP, IL-6, TNF-α	NM

^a^ Ages were displayed as mean ± standard deviation or mean.

Abbreviation: NM, not mentioned; FBG, fasting blood glucose; 2hPBG, 2-h Postprandial blood glucose; HbA1c, Glycosylated hemoglobin; VEGF, vascular endothelial growth factor; SBP, systolic pressure; DBP, diastolic pressure; HRV, heart rate variability; QTd, QT dispersion; HR, heart rate; CRP, C-reaction protein; IL, interleukin; TNF, tumor necrosis factor.

#### 4.2.8 TCM for elderly DOP

##### 4.2.8.1 Traditional Chinese Prescription

Nine studies explored the efficacy of Traditional Chinese Prescription in elderly patients with DOP, involving a total of 934 subjects. The age range of the included population ranged from 52 to 84 years, and the intervention duration was 12 weeks to 6months (Table 4; Supplementary Table S11). (Li, 2015; Hu et al., 2017; ZONG and ZHANG, 2017; Li et al., 2018c; LIU et al., 2018; ZHANG, 2019; Xiao, 2020; Yang, 2021b; Lin, 2021) Chinese medicines commonly used in nine prescriptions include *Epimedium sagittatum (Siebold & Zucc.) Maxim.* [Berberidaceae, Epimedii folium] (7 times), *Angelica sinensis (Oliv.) Diels* [Apiaceae, Angelicae sinensis radix] (6 times), *Rehmannia glutinosa (Gaertn.) DC.* [Orobanchaceae, Rehmanniae radix praeparata] (6 times), *Astragalus mongholicus Bunge* [Fabaceae, Astragali radix] (5 times) (Supplementary Table S12).

**TABLE 4:** TCM for elderly DOP.

Study	Subjects (Age range)	Ages^a^	No. of intervention group/control group	Treatment of intervention group^b^	Dose of intervention group	Treatment of control group	Dose of control group	Duration	Outcomes^c^	Adverse reactions
Traditional Chinese Prescription										
Liu et al. (2018)	Elderly DOP	NM	100/100	Bushen Huoxue Prescription + Calcium Carbonate and Vitamin D3 Tablets + Alendronate sodium + Alfacalcidol Soft Capsules	200 mL, 2/d	Calcium Carbonate and Vitamin D3 Tablets + Alendronate sodium + Alfacalcidol Soft Capsules	Calcium Carbonate and Vitamin D3 Tablets: 600 mg, 1/d; Alendronate sodium: 70 mg, 1/week; Alfacalcidol Soft Capsules: 1 ug, 1/d	24 weeks	BMD, P1NP, BGP, Ca^*^, P^*^, ALP^*^	NM
Li et al. (2018b) (2)	Elderly DOP (60–75 years old)	control group: 62.2 ± 3.7 years old; intervention group: 63.4 ± 2.8 years old	40/40	Bushen Yigu Recipe + Calcium Carbonate and Vitamin D3 Tablets	100 mL, 2/d	Calcium Carbonate and Vitamin D3 Tablets	Calcium Carbonate and Vitamin D3 Tablets: 600 mg, 1/d	3 months	BMD	NM
Lin (2021)	Elderly DOP (62–81 years old)	70.2 ± 4.1 years old	60/60	Bushen Zhuanggu Prescription + Calcium Carbonate and Vitamin D3 Tablets + Alendronate sodium	1 dose, 2/d	Calcium Carbonate and Vitamin D3 Tablets + Alendronate sodium	Calcium Carbonate and Vitamin D3 Tablets: 600 mg, 1/d; Alendronate sodium: 10 mg, 1/week	12 weeks	FBG^*^, HbA1c^*^, VAS	control group: gastrointestinal discomfort (2); intervention group: rash (1), gastrointestinal discomfort (2)
Zong and Zhang (2017)	Elderly DOP with Spleen and Stomach Qi Deficiency syndrome	control group: 66.60 ± 4.3 years old; intervention group: 65.80 ± 4.2 years old	36/30	Qishu Tanggu Decoction + Bisphosphonate + Calcium Carbonate and Vitamin D3 Tablets+	1 dose, 2/d	Bisphosphonate + Calcium Carbonate and Vitamin D3 Tablets	NM	3 months	BMD	NM
Zhang et al. (2019)	Elderly DOP (56–80 years old)	control group: 61.94 ± 2.73 years old; intervention group: 62.13 ± 2.61 years old	92/92	ShenTong ZhuYu Decoction + Calcium Carbonate and Vitamin D3 Tablets + Yougui Pills	1 dose, 2/d	Calcium Carbonate and Vitamin D3 Tablets + Yougui Pills	Calcium Carbonate and Vitamin D3 Tablets: 1 tablet, 2/d; Yougui Pills: 9 g, 2/d	24 weeks	BMD, BGP, NTX, WBV, FIB, PV, HCT	NM
Xiao (2020)	Elderly DOP (>60 years old)	control group: 68.6 ± 4.6 years old; intervention group: 68.4 ± 4.9 years old	42/42	Tonifying Kidney and Strengthening Bone Prescription + Calcium Carbonate and Vitamin D3 Tablets + Alendronate sodium + Alfacalcidol Soft Capsules	200 mL, 2/d	Calcium Carbonate and Vitamin D3 Tablets + Alendronate sodium + Alfacalcidol Soft Capsules	Calcium Carbonate and Vitamin D3 Tablets: 600 mg, 1/d; Alendronate sodium: 10 mg, 1/week; Alfacalcidol Soft Capsules: 0.5 ug, 1/d	3 months	FBG^*^, 2hPBG^*^, HbA1c^*^, BMD	NM
Li (2015)	Elderly DOP (52–76 years old)	control group: 65.9 ± 4.3 years old; intervention group: 65.4 ± 4.1 years old	33/32	Traditional Chinese Medicine Formulas + Gliclazide	125 mL, 2/d	Gliclazide	30 mg, 1/d	6 months	VAS, osteodynia relief time	NM
Yang (2021b) (2)	Elderly DOP (65–84 years old)	control group: 73.43 ± 4.63 years old; intervention group: 73.41 ± 4.62 years old	37/37	Zishen Jiangtang Pills + Conventional Western Medicine Treatment + Alendronate sodium	6 g, 3/d	Alendronate sodium	70 mg/week	3 months	FBG, 2hPBG, BMD, Bone-alkaline phosphatase, osteocalcin, β-CTX, Osteoprotegerin	no adverse reaction
Hu et al. (2017)	Elderly T2DM	control group: 65.13 ± 4.44 years old; intervention group: 64.77 ± 4.66 years old	31/30	Zuogui Pills + Alfacalcidol	9 g, 2/d	Alfacalcidol	0.25 ug, 2/d	6 months	FROP-COM, PⅠNP, β1-CTX, 25-OHD, ALP	NM
Traditional Chinese patent medicines										
Yu and Xu (2016)	Elderly DOP (51–75 years old)	NM	30/30	Jintiange Capsules + Atorvastatin	3 capsules, 3/d	Atorvastatin	10 mg, 1/d	24 weeks	BMD	no adverse reaction
Song (2016)	Elderly DOP (49–74 years old)	NM	50/50	Jintiange Capsules + Atorvastatin+	3 capsules, 3/d	Atorvastatin	10 mg, 1/d	24 weeks	BMD	no adverse reaction
Gao (2012)	Elderly DOP (60–75 years old)	control group: 66.41 ± 6.08 years old; intervention group: 65.62 ± 5.41 years old	48/48	Tangmaikang Granules^†^+Calcium Carbonate and Vitamin D3 Tablets + Alendronate sodium Tablets	5g, 3/d	Calcium Carbonate and Vitamin D3 Tablets + Alendronate sodium Tablets	Calcium Carbonate and Vitamin D3 Tablets: 600 mg, 1/d; Alendronate sodium Tablets: 10 mg, 1/d	26 weeks	BGP, NTX, BMD, Ca^*^, P^*^, ALP^*^	control group: abdominal distension (2); intervention group: nausea (1)
Traditional Chinese Medicine Extracts										
Luo et al. (2014)	Elderly DOP	69.4 ± 3.7 years old	50/50	Qianggu Capsules + Atorvastatin + Calcium Carbonate and Vitamin D3 Tablets	1 capsule, 1/d	Calcium Carbonate and Vitamin D3 Tablets + Atorvastatin	Calcium Carbonate and Vitamin D3 Tablets: 1 tablet, 1/d; Atorvastatin: 1 tablets, 1/d	6 months	FBG, 2hPBG, HbA1c, BMI^*^, VAS, BMD	control group: gastrointestinal discomfort (5); intervention group: gastrointestinal discomfort (7)

^a^ Ages were displayed as mean ± standard deviation or mean.

^b^ “^†^” indicated that it was included in Pharmacopoeia of the People’s Republic of China 2020.

^c^ “*” showed no significant difference between the intervention group and the control group.

Abbreviation: NM, not mentioned; BMD, bone mineral density; FBG, fasting blood glucose; VAS, visual analogue scale; BGP, osteocalcin; 2hPBG, 2-h postprandial blood glucose; HbA1c, Glycosylated hemoglobin; NTX, Cross-linked N-telopeptides of type I collagen; P1NP, aminoterminal prepeptide type Ⅰ procollagen; β1-CTX, β-1 collagen carboxy terminal peptide; BMI, body mass index; ALP, alkaline phosphatase; ALP, alkaline phosphatase; FIB, fibrinogen; PV, plasma viscosity; HCT, hematocrit; WBV, whole blood viscosity; P, phosphorus; Ca, Calcium; 25-OHD, 25-hydroxyvitamin D; FROP-COM, falls risk for older people in the community; PⅠNP, Type I procollagen amino-terminal peptide; β-CTX, Type I collagen carboxy-terminal peptide.

##### 4.2.8.2 Traditional Chinese patent medicines

Three studies explored the efficacy of Traditional Chinese patent medicines in elderly DOP, involving a total of 256 participants. The age range of the included population ranged from 49 to 75 years, and the intervention period was 24 weeks–26 weeks (Table 4; Supplementary Table S11). (Gao, 2012; Song, 2016; Yu and Xu, 2016) The study involved two kinds of Traditional Chinese patent medicines, Jintiange Capsules and Tangmaikang Granules. Tangmaikang Granules were included in Pharmacopoeia of the People’s Republic of China 2020 (Committee, 2020).

##### 4.2.8.3 Traditional Chinese Medicine Extracts

One study explored the efficacy of Chinese herbal extracts in elderly patients with diabetes mellitus combined with osteoporosis. A total of 100 subjects were included. The age of the included population was 69.4 ± 3.7 years old, and the intervention time was 6 months (Table 4; Supplementary Table S11).

After using Qianggu Capsule for intervention, (LUO et al., 2014) found that Qianggu Capsule can reduce pain and improve bone density in elderly DOP patients while improving glucose metabolism. The main component of Qianggu Capsule is *Drynaria roosii Nakaike* [Polypodiaceae, Drynariae rhizoma] total flavone, which has been found to improve DOP by activating BMP2/Smad signaling pathway, promoting bone formation and inhibiting bone resorption (Fang et al., 2023).

#### 4.2.9 TCM for elderly diabetes with cognitive impairment

##### 4.2.9.1 Traditional Chinese Prescription

Six studies explored the efficacy of TCM compounds in elderly patients with diabetes mellitus combined with cognitive dysfunction. A total of 462 participants were enrolled, ranging in age from 50 to 84 years, and the intervention period was 12 weeks to 6months (Table 5). (Zhao et al., 2016a; Liu et al., 2016; Gao et al., 2017; Yan and Guan, 2019; Zhao et al., 2019; Yu et al., 2022) The studies involved five prescriptions, commonly used Chinese medicine including *Rehmannia glutinosa (Gaertn.) DC.* [Orobanchaceae, Rehmanniae Radix] (5 times), *Acorus calamus* var. *angustatus Besser* [Acoraceae, Acori tatarinowii rhizoma] (4 times), *Panax ginseng C.A.Mey.* [Araliaceae, Ginseng radix et rhizoma] (4 times), *Astragalus mongholicus Bunge* [Fabaceae, Astragali radix] (4 times) (Supplementary Table S13; Supplementary Table S14). Three of the studies limited inclusion to participants with mild cognitive impairment.

**TABLE 5:** TCM for elderly diabetes with cognitive impairment.

Study	Subjects (Age range)	Ages^a^	No. of intervention group/control group	Treatment of intervention group^b^	Dose of intervention group	Treatment of control group	Dose of control group	Duration	Outcomes^c^	Adverse reactions
Traditional Chinese Prescription										
Yu et al. (2022)	Elderly T2DM with mild cognitive impairment of deficiency of spleen and kidney combined with blood stasis (61–84 years old)	control group: 71.10 ± 6.12 years old; intervention group: 71.67 ± 6.09 years old	40/40	Bushen Jianpi Huoxue Formula + Aspirin	125 mL, 2/d	Aspirin	100 mg, 1/d	3 months	MoCA, FBG, 2hPBG, HbA1c, HOMA-IR^*^, CRP	control group: anemia (1), proteinuria (2), abnormal liver function (1), abnormal renal function (2), hypotension (2); intervention group: anemia (0), proteinuria (1), abnormal liver function (0), abnormal renal function (0), hypotension (0)
Gao et al. (2017)	Elderly T2DM with cognitive impairment (50–75 years old)	control group: 62.08 ± 5.92 years old; intervention group: 62.40 ± 5.95 years old	60/60	Chinese Medicine for nourishing kidney, eliminating phlegm and damp + Donepezil	1 dose, 2/d	Donepezil	5 mg, 1/d	6 months	MoCA, MMSE, ADL, MDA, SOD, AchE	control group: dizzy (2), headache (1), hypotension (1), flushed face (0), vomit (1); intervention group: dizzy (3), headache (2), hypotension (0), flushed face (1), vomit (2)
Zhao et al. (2016a) (4)	Elderly T2DM with mild cognitive impairment (60–80 years old)	NM	30/30	Tonifying Deficiency for Dispelling Turbidity and Removing Obstruction in Collaterals Method + Conventional Western Medicine Treatment	100 mL, 2/d	Conventional Western Medicine Treatment	NM	12 weeks	MMSE, ADAS-COG-DVR, adiponectin, Leptin, HOMA-IS	NM
Zhao et al. (2016b) (5)	Elderly T2DM with mild cognitive impairment (60–80 years old)	71.9 ± 8.2 years old	36/36	Tonifying deficiency, removing turbidity and dredging collaterals recipe + Conventional Western Medicine Treatment	150 mL, 2/d	Conventional Western Medicine Treatment	NM	12 weeks	MMSE, ADAS-COG-DVR, A-β, SOD	NM
Liu et al. (2016)	Elderly T2DM with cognitive impairment (60–80 years old)	control group: 68.2 ± 6.9 years old; intervention group: 65.6 ± 7.6 years old	35/35	Yiqi bushen huoxue Decoction + Conventional Western Medicine Treatment	150mL, 2/d	Conventional Western Medicine Treatment	NM	12 weeks	MMSE, CDR, ADL	NM
Yan and Guan (2019)	Elderly T2DM with cognitive impairment (65–80 years old)	control group: 70.56 ± 5.26 years old; intervention group: 71.37 ± 4.66 years old	30/30	Yizhi Heji + Citicoline Sodium Capsules	50 mL, 2/d	Citicoline Sodium Capsules	0.2 g, 3/d	6 months	MMSE, FBG, 2hPBG, HbA1c, WBC, RBC, Hb, ALT, AST, BUN, Cr	no adverse reaction
Traditional Chinese patent medicines										
Guo et al. (2020)	Elderly T2DM with mild cognitive impairment (60–79 years old)	control group: 68.2 ± 5.8 years old; intervention group: 67.8 ± 5.4 years old	48/48	Jinlida Granules^†^+Sitagliptin	9g, 3/d	Sitagliptin	100 mg, 1/d	3 months	MMSE, MoCA, FBG, HbA1c, IL-1β, IL-6, TNF-α	control group: hypoglycemia (3); intervention group: hypoglycemia (4)
Zhao and He (2017)	Elderly T2DM with cerebral microvascular lesion blood viscosity (61–78 years old)	control group: 67.5 ± 3.6 years old; intervention group: 67.24 ± 4.0 years old	50/50	Xiaoke pill^†^+Metformin	10 pills, 3/d	Metformin	0.75 g, 3/d	12 weeks	cognitive function scores, WBV, PV, HCT, Cell deformation index, Erythrocyte rigidity index, Erythrocyte deformation index	NM
Traditional Chinese Medicine Extracts										
Wang 2012 (2)	Elderly T2DM (>60 years old)	NM	94/96	Ginkgo biloba extract Capsules + Conventional Western Medicine Treatment	0.3 g, 2/d	Conventional Western Medicine Treatment	NM	6 months	MMSE, CDR, ADL, CDT, BDNF, Hcy	NM

^a^ Ages were displayed as mean ± standard deviation or mean.

^b^ “^†^” indicated that it was included in Pharmacopoeia of the People’s Republic of China 2020.

^c^ “*” showed no significant difference between the intervention group and the control group.

Abbreviation: NM, not mentioned; 2hPBG, 2-h postprandial blood glucose; AchE, acetylcholinesterase; ADAS-COG-DVR, Alzheimer disease assessment scale-cog; ADL, Activity of DailyLiving Scale; ALT, alanine aminotransferase; AST, aspartate aminotransferase; A-β, amyloid β-protein; BDNF, serum brain derived growth factor; BUN, blood urea nitrogen; CDR, clinical dementia rating scale;CDT, clock drawing test; Cr, Creatinine; CRP, C-reaction protein; FBG, fasting blood glucose; Hb, Haemoglobin; HbA1c, Glycosylated hemoglobin; WBV, whole blood viscosity; HCT, hematocrit; Hcy, Homocysteine; HOMA, homeostasis model assessment; IL, interleukin; MDA, malondialdehyde; MMSE, minimum mental state examination; MoCA, montreal cognitive assessment; PV, plasma viscosity; RBC, erythrocyte; SOD, superoxide dismutase; TNF, tumor necrosis factor; WBC, leukocyte.

##### 4.2.9.2 Traditional Chinese patent medicines

Two studies explored the efficacy of Traditional Chinese patent medicines in elderly patients with diabetes mellitus combined with cognitive dysfunction. A total of 196 subjects were included, ranging in age from 60 to 79 years old, and the intervention period was 12 weeks to 3 months (Table 5; Supplementary Table S13). The Traditional Chinese patent medicines involved in the studies included Jinlida Granules and Xiaoke Pills, which are included in Pharmacopoeia of the People’s Republic of China 2020 (Committee, 2020). Guo et al. (2020) found that Jinlida Granules can improve MMSE and MoCA scores. Zhao and He (2017) found that Xiaoke Pills had positive effects on cognitive function and blood viscosity in elderly patients with diabetes mellitus accompanied by cerebrovascular disease.

##### 4.2.9.3 Traditional Chinese Medicine Extracts

One study explored the effect of TCM extracts on cognitive dysfunction in elderly diabetic patients, including 190 subjects. (Wang, 2012). The age standard of the included population was >60 years old, and the intervention time was 6 months (Table 5; Supplementary Table S13). The study included elderly diabetic patients without cognitive impairment, and found that ginkgo biloba extract can also improve the MMSE, CDR and ADL scores of patients, indicating that it has a positive effect on the cognitive function of elderly diabetic patients.

#### 4.2.10 TCM for elderly diabetes with vascular injury

##### 4.2.10.1 Traditional Chinese Prescription

Three RCTs involving 286 elderly patients with diabetes and vascular sclerosis were conducted. The age range of the included population was 60–79 years, and the intervention duration was 12 weeks to 4 months (Table 6; Supplementary Table S15). Cheng et al. (2019); Guan (2021) applied Modified Huangqi Guizhi Wuwu Tang and Tangmai Tongluo Decoction to treat the elderly diabetic patients with lower extremity vascular lesions, and found that the decoctions could improve the blood flow of dorsal foot artery after 3 months of intervention. After intervention with Yiqi Tongluo Qingre cream, (Liu et al., 2022) found that it could improve arteriosclerosis of common carotid artery, popliteal artery and dorsal foot, and reduce lipid metabolism indicators.

**TABLE 6:** TCM for elderly diabetes with vascular injury.

Study	Subjects (Age range)	Ages^a^	No. of intervention group/control group	Treatment of intervention group^b^	Dose of intervention group	Treatment of control group	Dose of control group	Duration	Outcomes^c^	Adverse reactions
Traditional Chinese Prescription										
Cheng et al. (2019)	Elderly T2DM with lower-extremity arterial disease (≤Fontaine stage Ⅲ, 60–75 years old)	control group: 63.08 ± 9.17 years old; intervention group: 61.53 ± 8.84 years old	64/64	Modified Huangqi Guizhi Wuwu Decoction+Probucolum+Aspirin+Alprostadil Injection	150 ml, 2/d	Probucolum+Aspirin+Alprostadil Injection	Probucolum: 0.5 g, 2/d; Aspirin: 100 mg, 1/d; Alprostadil Injection: 10 ug, 1/d	3 months	ABI, TBI, Dorsal foot artery blood vessel, IL-1, Hcy, TNF-α, hs-CRP, CysC, MDA, SOD, LDL	NM
Guan (2021)	Elderly T2DM with lower limb arterial disease (60–76 years old)	control group: 67.65 ± 5.28 years old; intervention group: 67.63 ± 5.35 years old	39/39	Tangmai Tongluo Decoction+Aspirin+Probucol+Alprostadil Injection	150 ml, 2/d	Aspirin+Probucol+Alprostadil Injection	aspirin: 50 mg, 2/d; Probucol: 0.5 g, 2/d; Alprostadil Injection: 10 ug, 1/d	12 weeks	Blood flow velocity of dorsalis pedis artery, Peak flow velocity of dorsal foot artery blood flow, TNF-α, IL-1, MWD, PWD	NM
Liu et al. (2022)	Elderly patients with T2DM and atherosclerosis (61–79 years old)	control group: 70.2 ± 5.9 years old; intervention group: 69.7 ± 6.1 years old	40/40	Yiqi Tongluo Qingre cream+Conventional Western Medicine Treatment	10 g, 2/d	Conventional Western Medicine Treatment	NM	4 months	TC, TG, LDL, HDL, FIB, ABI, Dorsal foot artery blood vessel, QoL	control group: vomit (3), diarrhea (4), loss of appetite (7), abnormal liver function (3), bleeding (6); intervention group: vomit (0), diarrhea (1), loss of appetite (0), abnormal liver function (0), bleeding (0)
Traditional Chinese patent medicines										
Wang et al. (2013) (2)	Elderly T2DM with arteriosclerosis obliterans of lower limbs (60–81 years old)	control group: 68.5 years old; intervention group: 71.2 years old	58/57	Maixuekang capsule^†^+Yixinshu Capsules+Clopidogrel+Rosuvastatin	3 capsules, 1/d	Clopidogrel+Rosuvastatin	Clopidogrel: 75 mg, 1/d; Rosuvastatin: 10 mg, 1/d	3 months	ERS, FIB, Platelet, HbA1c, LDL	no adverse reaction
Wang et al. (2017)	Elderly T2DM with subclinical atherosclerosis (62–75 years old)	65.6 ± 4.1 years old	55/55	Naoxintong Capsules^†^+Conventional Western Medicine Treatment	3 capsules, 3/d	Conventional Western Medicine Treatment	NM	6 months	C-IMT, PAI-1, β-thromboglobulin, P-selectin	NM
Shou et.al. (2012)	Elderly T2DM with coronary heart disease (≥65 years old)	76.06 ± 4.81	72/76	Shexiang Baoxin Pills^†^+Conventional Western Medicine Treatment	2 pills, 3/d	Conventional Western Medicine Treatment	NM	12 weeks	FBG, HbA1c, TC, LDL, hs-CRP, FIB, ABI, CAVI	no adverse reaction
Yu and Zhang (2018)	Elderly T2DM with coronary heart disease (NM)	NM	55/55	Yangxinshi Tablets^†^+Conventional Western Medicine Treatment	3 tablets, 3/d	Conventional Western Medicine Treatment	NM	12 weeks	Number of angina attacks, 6-minute walking distance, METs	NM
Traditional Chinese Medicine Extracts										
Li and Yang (2017)	Elderly T2DM with carotid atherosclerosis (60–79 years old)	control group: 66.2 ± 4.5 years old; intervention group: 66.5 ± 4.2 years old	75/75	Tanshinone Capsules+Beraprost Sodium	2 capsules, 3/d	Beraprost Sodium	40 ug, 2/d	3 months	FBG, HbA1c, TG, TC, C-IMT, Crouse scores, MCP-1, hs-CRP, TNF-α, ICAM-1, Vascular endothelial cell function	NM
Jiang et al. (2021)	Elderly T2DM with peripheral arterial disease (70–90 years old)	control group: 80.2 ± 6.5 years old; intervention group: 80.8 ± 5.8 years old	50/50	panax notoginseng saponins+Conventional Western Medicine Treatment	100 mg, 3/d	Conventional Western Medicine Treatment+placebo	2 capsules, 2/d	3 months	hs-CRP, IL-6, TNF-α, TC, TG, HDL, LDL, FBG, HbA1c, FIB, C-IMT^*^	NM
Li et al. (2016a) (3)	Elderly T2DM with carotid plaque (60–84 years old)	69.93 ± 6.79 years old	48/48	Xinnao Shutong Tablets+Conventional Western Medicine Treatment	0.52 g, 3/d	Conventional Western Medicine Treatment	Metformin: 0.5–1 g, 2/d; gliclazide sustained-release tablets: 30–60 mg, 1/d; Acarbose:50–100 mg, 3/d; protamine biosynthetic human insulin injection (pre mixed 30R): NM, 2/d; aspirin: 100 mg, 1/d; atorvastatin calcium: 20 mg, 1/d	12 weeks	C-IMT, Crouse scores, Hcy, hs-CRP, FBG, HbA1c, TC, TG, LDL, HDL	no adverse reaction
Chen (2017)	Elderly T2DM with carotid atherosclerosis (60–75 years old)	control group: 67.4 ± 6.7 years old; intervention group: 67.1 ± 6.9 years old	43/43	Ginkgo biloba leaves+Rosuvastatin calcium	400 mg, 3/d	Rosuvastatin calcium	10 mg, 1/d	3 months	TC, TG, LDL, HDL, NO, ET-1, MDA	control group: diarrhea (1), dizzy (1); intervention group: diarrhea (1), dizzy (2)

^a^ Ages were displayed as mean ± standard deviation or mean.

^b^ “^†^” indicated that it was included in Pharmacopoeia of the People’s Republic of China 2020.

^c^ “*” showed no significant difference between the intervention group and the control group.

Abbreviation: NM, not mentioned; BMD, bone mineral density; FBG, fasting blood glucose; VAS, visual analogue scale; BGP, osteocalcin; 2hPBG, 2-h postprandial blood glucose; HbA1c, Glycosylated hemoglobin; NTX, Cross-linked N-telopeptides of type I collagen; P1NP, aminoterminal prepeptide type Ⅰ procollagen; β1-CTX, β-1 collagen carboxy terminal peptide; BMI, body mass index; ALP, alkaline phosphatase; ALP, alkaline phosphatase; FIB, fibrinogen; PV, plasma viscosity; HCT, hematocrit; WBV, whole blood viscosity; P, phosphorus; Ca, Calcium; 25-OHD, 25-hydroxyvitamin D; FROP-COM, falls risk for older people in the community; PⅠNP, Type I procollagen amino-terminal peptide; β-CTX, Type I collagen carboxy-terminal peptide.

##### 4.2.10.2 Traditional Chinese patent medicines

Four RCTs investigated the efficacy of Traditional Chinese patent medicines in elderly patients with diabetes and vascular sclerosis. A total of 483 subjects were enrolled. The age of the enrolled population ranged from 60 to 81 years, and the intervention time was 12 weeks to 6 months (Table 6; Supplementary Table S15) (SHOU et al., 2012; Wang, 2013; Wang et al., 2017; Yu and Zhang, 2018).The studies involved five Traditional Chinese patent medicines. Naoxintong Capsules, Yixinshu Capsules, Yangxinshi Tablets and Shexiang Baoxin Pills were included in Pharmacopoeia of the People’s Republic of China 2020 (Committee, 2020), Maixuekang Capsules was not included.

##### 4.2.10.3 Traditional Chinese Medicine Extracts

Four RCTs involving 432 participants explored the efficacy of Traditional Chinese Medicine Extracts in elderly diabetic patients with vascular sclerosis. The age range of the included population was 60–90 years old, and the intervention time was 12 weeks to 3 months (Table 6; Supplementary Table S15) (Li et al., 2016a; Chen, 2017; Li and Yang, 2017; JIANG et al., 2021). The studies involved 4 extracts, including *Panax notoginseng (Burk.) F.H.Chen* [Araliaceae, Notoginseng total saponins], *Salvia miltiorrhiza Bunge* [Lamiaceae, Tanshinones], *Ginkgo biloba L.* [Ginkgoaceae, Ginkgo leaves extract], *Tribulus terrestris L.* [Zygophyllaceae, Tribuli fructus]. Both of them are used to treat carotid atherosclerosis.

#### 4.2.11 Safty

Adverse reactions were reported in 72 studies, of which 23 reported no adverse reactions. Among the adverse events studies, there were 48 adverse events in the control group, including 30 symptoms, involving 222 subjects, and common adverse events included hypoglycemia (13 RCTs, 42 subjects), gastrointestinal discomfort (7 RCTs, 19 subjects), dizzy (13 RCTs, 18 subjects), loss of appetite (8 RCTs, 18 subjects), diarrhea (9 RCTs, 17 subjects), vomit (9 RCTs, 16 subjects), nausea (9 RCTs, 13 subjects), headache (8 RCTs, 11 subjects); Adverse events occurred in 47 studies in the intervention group, with 26 symptoms and involving 167 participants. Common adverse events included gastrointestinal discomfort (9 RCTs, 24 subjects), dizzy (11 RCTs, 22 subjects), nausea (12 RCTs, 18 subjects), hypoglycemia (10 RCTs, 17 subjects), diarrhea (9 RCTs, 14 subjects), vomit (9 RCTs, 13 subjects), loss of appetite (5 RCTs, 10 subjects), rash (5 RCTs, 9 subjects) (Supplementary Table S16).

#### 4.2.12 Quality assessment and ConPhyMP statement

Jadad scale was used to evaluate the treatment of literatures, and there were 1 study with a score of 5, 13 studies with a score of 4, 61 studies with a score of 3, 82 studies with a score of 2, and 3 studies with a score of 1. Not use blind methods, failure to report dropout and loss of follow-up, and failure to explain the way of randomization performed were the main reasons for the low Jadad score (Supplementary Table S17).

Nine studies about Traditional Chinese Medicine Extracts were evaluated by the ConPhyMP statement. *Zea mays L.* [Poaceae, corn silk] was identified as type B extracts because corn silk was not included in Pharmacopoeia of the People’s Republic of China 2020 (Committee, 2020). Other extracts were identified as type A extracts. Detailed evaluation results were shown in the Supplementary Table S18–26.

## 5 Discussion

The elderly are often complicated with many diseases, and the phenomenon of multiple drug use is common. The study found that the prevalence of polypharmacy is approximately 50% in older people with diabetes and is associated with poor blood sugar control, risk of hypoglycemia, falls, fainting, hospitalization, and risk of death (Pazan and Wehling, 2021). The application of commonly used western anti-diabetic drugs in elderly diabetes seems to have both advantages and disadvantages. Cohort studies showed that metformin reduced the risk of dementia in T2DM patients by 35% over 8 years (Hsu et al., 2011), but a prospective study showed that metformin increased cognitive deterioration and risk of AD (Koo et al., 2019). A population-based nested case study also showed that metformin use was associated with an increased risk of AD (Ha et al., 2021). Clinical studies have shown that TZDs can improve the cognitive function of diabetic patients and reduce the risk of dementia (Watson et al., 2005; Abbatecola et al., 2010; Sato et al., 2011; Heneka et al., 2015; Burns et al., 2021), but there are also studies that the application of TZDs drugs has no significant benefits in improving Alzheimer’s disease and delaying Parkinson’s disease (Heneka et al., 2015; Burns et al., 2021). The effect of SGLT2i on bone is controversial. Many meta-analyses have shown that SGLT2i does not increase the risk of fracture in patients with T2DM (Tang et al., 2016; Ruanpeng et al., 2017), but there is more evidence that canagliflozin increases the risk of fracture (Watts et al., 2016; Blevins and Farooki, 2017), especially in patients with renal failure, cardiovascular disease, peripheral vascular disease, or neuropathy (Kalaitzoglou et al., 2019). In addition, the positive or negative effects of antidiabetic drugs on sarcopenia are not fully understood. Epidemiology shows that metformin or TZDs can reduce muscle loss in elderly IFG or T2DM men, but clinical observational studies have found that female skeletal muscle mass is significantly reduced after metformin treatment (Aghili et al., 2014). TCM has the advantages of a wide range of indications and many targets, and many studies have proved that the same prescription has a synergistic effect in the treatment of multiple diseases (Zhang et al., 2020; Yuan et al., 2023). For example, Jinlida Granules improve the islet function, kidney function and cognitive function of elderly diabetic patients. Compound Danshen Dripping Pills have a synergistic effect on improving DKD and DR in the elderly. Many epidemiological studies have verified the correlation between DKD and DR (Park et al., 2019).

In addition, Chinese medicine has certain safety in the treatment of elderly diabetes. The study found that the use of sulfonylureas would increase the risk of severe hypoglycemia by three times (Misra-Hebert et al., 2018). Elderly diabetes patients were older and complicated with a variety of chronic diseases, which was more likely to increase the risk of hypoglycemia caused by sulfonylureas (Sinclair et al., 2015). Compared with the control group, the number of adverse reactions in TCM intervention group was lower, especially the incidence of hypoglycemia was significantly lower than that in the control group.

However, there are still many problems in the clinical research of TCM in elderly diabetes. First, the age criteria for inclusion are different. In the international standard, patients with diabetes whose age is ≥65 years are defined as elderly diabetes (LeRoith et al., 2019; ElSayed et al., 2023), while in the Chinese Clinical Guidelines for the Prevention and Treatment of Elderly Type 2 diabetes (2022) (group, 2022), the age standard is ≥60 years. According to the included literature, it was found that many studies did not adhere to this age criterion although they focused on older patients with diabetes. Second, the subjects were not diagnosed clearly. Only some studies have defined the stage of DKD in the elderly, which may have different effects on the outcome depending on the severity of the patient’s disease. Third, the studies design is not rigorous. Most of the study controls were Western drugs, only one study used placebo control. Meanwhile, blind method was not used and the Jadad score was of low quality. Fourth, intervention measures are not standardized. Some studies did not explain the composition or dosage of the Traditional Chinese Prescription. Some of the traditional Chinese patent medicines used in the study were not included in Pharmacopoeia of the People’s Republic of China 2020 (Committee, 2020) and the description of the extraction process for traditional Chinese extracts is also not detailed. Both of them led to the unclear method of TCM in the treatment of elderly diabetes. Fifth, no adverse reactions were reported. Some studies have not reported adverse reactions, making it difficult to evaluate the safety of their treatment.

## 6 Conclusion

The application of TCM in elderly diabetes has the advantages of multi-target and coordinated treatment. TCM can jointly treat various complications and complications caused by aging, and can focus on the unique characteristics of elderly diabetes, such as vascular aging, osteoporosis, cognitive impairment, sarcopenia, etc. However, there are also many problems, including the inclusion of age criteria and diagnosis of subjects are unclear, imprecise research design, non-standard intervention measures, and its safety needs further exploration. These will lead to the low efficacy and safety of TCM in elderly diabetes. Although there have been many explorations of TCM in the experimental model of elderly diabetes, in the future, the diagnosis of elderly people with diabetes needs to be further clarified. Traditional Chinese patent medicines included in the pharmacopoeia can be used to conduct more rigorous RCTs, and then gradually standardize the traditional Chinese medicine prescriptions and traditional Chinese medicine extracts, providing higher level evidence for the treatment of elderly diabetes with traditional Chinese medicine.
