# Supplementary material for: Mechanism of traditional Chinese medicine in elderly diabetes mellitus and a systematic review of its clinical application
Source: Front Pharmacol. 2024 Mar 6;15:1339148. doi: 10.3389/fphar.2024.1339148 (PMC10953506; doi:10.3389/fphar.2024.1339148)
Supplement: Supplementary file 2 [file DataSheet1.zip › Supplementary Table S1-17/Supplementary Table S1.docx]

Supplementary Table S1 | TCM for elderly diabetes and islet function.

| Study | Subjects (Age range) | Ages^1^ | No. of intervention group/control group | Treatment of intervention group^2^ | Dose of intervention group | Treatment of control group | Dose of control group | Duration | Outcomes^3^ | Adverse reactions |
| --- | --- | --- | --- | --- | --- | --- | --- | --- | --- | --- |
| Traditional Chinese Prescription | | | | | | | | | | |
| Zhou 2014 | Elderly T2DM (60-86 years old) | 66.9 ±9.2 years old | 124/124 | Erban Decoction+Metformin | 1 dose, 2/d | Metformin | 500mg, 3/d | 3 months | FBG, 2hPBG, HbA1c, FINS, fasting c-peptide, HOMA-IR, HOMA-β | NM |
| Sun 2022 | Elderly T2DM of gastrointestinal damp-heat syndrome (60-77 years old) | control group: 69.33±0.58 years old; intervention group: 68.512±0.48 years old | 63/63 | Gegen Qinlian Decoction+Insulin Aspart Injection | 150ml, 2/d | Insulin Aspart Injection | 0.4 IU/kg*d | 3 months | FBG, 2hPBG, HbA1c, TC, TG, HDL, LDL, DSQL | NM |
| Jiang 2023 | Elderly T2DM of gastrointestinal damp-heat syndrome (65-82 years old) | control group: 73.54±3.71 years old; intervention group: 73.51±3.67 years old | 46/46 | Gegen Qinlian Decoction+Insulin Aspart Injection+Insulin Glargine Injection | 200ml, 2/d | Insulin Aspart Injection+Insulin Glargine Injection | Insulin Aspart Injection: adjust dosage based on blood sugar levels, 3/d; Insulin Glargine Injection: adjust dosage based on blood sugar levels, 1/d | 3 months | FBG, 2hPBG, HbA1c, FINS，HOMA-β, HOMA-IR | control group: diarrhea (2), hypoglycemia (2), vomit (1), dizzy (2); intervention group: diarrhea (1), hypoglycemia (0), vomit (1), dizzy (1) |
| Ni 2021 | Elderly T2DM of gastrointestinal damp-heat syndrome (60-81 years old) | control group: 73.00±1.79 years old; intervention group: 70.00±1.95 years old | 31/30 | Gegen Qinlian Decoction+Insulin Aspart Injection+Insulin Glargine Injection | 150ml, 2/d | Insulin Aspart Injection+Insulin Glargine Injection | Insulin Aspart Injection: 0.4IU/kg·d, 3/d; Insulin Glargine Injection: 0.4IU/kg·d, 1/d | 12 weeks | FBG, 2hPBG, HbA1c, FINS, BMI, HOMA-IR, TC, TG, HDL, LDL, DSQL | NM |
| Wang 2022 (1) | Elderly T2DM (60-85 years old) | control group: 72.28±6.54 years old; intervention group: 74.15±5.98 years old | 48/48 | Huanglian Wendan Decoction+Metformin | 200ml, 2/d | Metformin | 0.25g, 3/d. Subsequently, the dose increased by 0.75 g/d every 2 weeks, with a maximum dose of 2.25 g/d. | 12 weeks | FBG, 2hPBG, HbA1c, IL-6, IL-8 | NM |
| Zhou 2013 | Elderly T2DM (60-80 years old) | control group: 70.9 years old; intervention group: 71.6 years old | 30/30 | hypoglycemic basic formula+Metformin+Acarbose | 1 dose, 2/d | Metformin+Acarbose | Metformin: 0.5g, 2/d; Acarbose: 50mg, 3/d | 12 weeks | FBG, 2hPBG, HbA1c^*^ | control group: hypoglycemia (4); intervention group: hypoglycemia (1) |
| Liu 2016 | Elderly T2DM (60-78 years old) | control group: 67.6 years old; intervention group: 67.9 years old | 33/34 | Invigorating the Spleen, Invigorating Qi and Tonifying the Kidney Decoction+Gliquidone | 150ml, 2/d | Gliquidone | 15-30mg, 3/d | 3 months | FBG, HbA1c, β2-MG | no adverse reaction |
| Xue 2010 | Elderly T2DM (60-87 years old) | control group: 76.8±8.4 years old; intervention group: 74.2±7.6 years old | 30/30 | Jiangtangjing Granules+Conventional Western Medicine Treatment | 10g, 2/d | Conventional Western Medicine Treatment+Fluvastatin | Fluvastatin: 40mg, 1/d | 12 weeks | FBG, 2hPBG, HbA1c, TC, TG, LDL, HDL, ISI | control group: nausea (3), loss of appetite (2); intervention group: nausea (3) |
| Zhu 2013 | Elderly T2DM (60-87 years old) | control group: 76.8±8.4 years old; intervention group: 74.2±7.6 years old | 30/30 | Jiangtangjing Granules+Conventional Western Medicine Treatment | 10g, 2/d | Conventional Western Medicine Treatment | NM | 12 weeks | FBG, FINS, HbA1c, TC, TG, HDL, LDL, P-selectin, TpP, tHcy, APTT, PT, TT, FIB | control group: none; intervention group: nausea (3) |
| Fu 2013 | Elderly T2DM (50-74 years old) | NM | 33/32 | Jiawei Yuye Decoction+Conventional Western Medicine Treatment | 1 dose, 2/d | Conventional Western Medicine Treatment | NM | 3 months | FBG, 2hPBG, HbA1c | NM |
| Ding 2020 | Elderly T2DM (73-91 years old) | control group: 80.6±4.2 years old; intervention group: 79.2±5.0 years old | 67/50 | Jingui Shenqi Prescription+OADS (except for insulin sensitizers) | 1 dose, 2/d | OADS (except for insulin sensitizers) | NM | 3 months | FBG, HbA1c, BMI, FINS, ISI, norepinephrine, dopamine, Serum adrenocorticotropic hormone, cortisol | NM |
| Wu 2015 | Elderly T2DM (60-89 years old) | NM | 40/40 | Jinlian Mixture+Glimepiride | 150ml, 2/d | Glimepiride | 2mg, 1/d | 3 months | FBG, 2hPBG, HbA1c, TC, TG, CRP, TNF-α | NM |
| Ma 2022 | Elderly T2DM (61-78 years old) | control group: 68.0 years old; intervention group: 68.5 years old | 45/45 | Liuwei Dihuang Decoction+Acarbose | 1 dose, 2-3/d | Acarbose | 50mg, 3/d | 3 months | FBG, 2hPBG, HbA1c, FINS^*^, SF-36^*^ | control group: hypoglycemia (7); intervention group: hypoglycemia (1) |
| Zhu 2021 | Elderly T2DM (60-76 years old) | control group: 67.06±3.38 years old; intervention group: 67.22±3.15 years old | 54/54 | Liuwei Dihuang Decoction+Sitagliptin Phosphate | 200ml, 2/d | Sitagliptin Phosphate | 100mg, 1/d | 12 weeks | FBG, 2hPBG, HbA1c, CRP, IL-17A, IL-23 | NM |
| Zhao 2022 | Elderly T2DM of kidney Yin deficiency type (65-80 years old) | control group: 74.03±7.85 years old; intervention group: 73.89±6.57 years old | 40/40 | Liuwei Dihuang Pills+Metformin | 200ml, 2/d | Metformin | 0.5g, 3/d | 12 weeks | FBG, 2hPBG, HbA1c, TC, TG, HDL, LDL | control group: loss of appetite (1), feeble (2), nausea (2), abdominal distension (1); intervention group: loss of appetite (2), feeble (0), nausea (1), abdominal distension (1) |
| Zha 2022 | Elderly T2DM (60-93 years old) | control group: 81.05±8.42 years old; intervention group: 80.87±9.05 years old | 50/50 | Modified Shenqi Dihuang Decoction+Isophane Protamine Recombinant Human Insulin Injection | 150ml, 2/d | Isophane Protamine Recombinant Human Insulin Injection | 20-60U/kg, 1/d | 3 months | FBG, 2hPBG, HbA1c, TC, TG, HDL, LDL | control group: gastrointestinal discomfort (0), dry (1), abnormal liver function (1), feeble (2); intervention group: gastrointestinal discomfort (1), dry (0), abnormal liver function (0), feeble (1) |
| Zou 2017 | Elderly DM with Insulin Resistance (60-86 years old) | control group: 74.08±5.44 years old; intervention group: 74.52±5.39 years old | 73/73 | Modified Taohe Chengqi Decoction+Metformin | 1 dose, 3/d | Metformin | 0.25g, 3/d | 3 months | FBG, HbA1c, INS, SBP, DBP, HOMA-IR | NM |
| Han 2023 (1) | Elderly T2DM (60-81 years old) | control group: 63.7±4.9 years old; intervention group: 60.7±5.3 years old | 40/40 | Renshen Baihu Decoction+Metformin+Gliclazide | 1 dose/d | Metformin+Gliclazide | Metformin: 0.25g, 2-3/d; Gliclazide: 40mg, 2/d | 3 months | FBG, 2hPBG, HbA1c, Lp(a), Adiponectin, CRP | control group: nausea (1), vomit (2), diarrhea (1); intervention group: nausea (1), vomit (1), diarrhea (0) |
| Wei 2021 | Elderly T2DM (60-76 years old) | control group: 67.76±3.39 years old; intervention group: 67.12±3.07 years old | 64/63 | Sanhuang Decoction+Liraglutide | 100ml, 2/d | Liraglutide | 0.6-1.8mg, 1/d | 3 months | FBG, 2hPBG, HbA1c, HOMA-IR, TC, TG, ApoB, TNF-α, IL-6, hs-CRP | NM |
| Ailiyasi 2019 | Elderly T2DM (60-73 years old) | control group: 65.23±3.24 years old; intervention group: 65.17±3.16 years old | 66/66 | Sanhuang Decoction+Liraglutide Injection | 100ml, 2/d | Liraglutide Injection | 0.6-1.8mg, 1/d | 3 months | FBG, HbA1c, HOMA-IR, TC, TG, ApoB, MDA, CSH-Px, CAT, SOD, ADP, TNF-α, IL-6, hs-CRP | control group: vomit (1), hypoglycemia (2), diarrhea (2), acute pancreatitis (2); intervention group: vomit (1), hypoglycemia (1), diarrhea (1), acute pancreatitis (1) |
| XU 2020 | Elderly T2DM (63-76 years old) | control group: 70.32±3.48 years old; intervention group: 70.18±3.55 years old | 68/68 | self-designed Yangyin Xiaoke Recipe+Metformin | 250ml, 2/d | Metformin | 0.25g, 2/d | 3 months | FBG, 2hPBG, HbA1c, FINS, ISI, HOMA-IR, HOMA-β, TG, LDL | NM |
| Wang 2013 (1) | Elderly T2DM (60-75 years old) | control group: 67±7 years old; intervention group: 67±7 years old | 90/70 | Shendi Shengjin Capsules+Glimepiride | 1.5g, 3/d | Glimepiride | 2mg, 1/d | 4 months | HbA1c, fasting C-peptide | control group: hypoglycemia (5); intervention group: hypoglycemia (3) |
| Li 2014 | Elderly T2DM with deficiency of both qi and yin syndrome (60-81 years old) | control group: 67.84±7.93 years old; intervention group: 68.23±8.11 years old | 34/34 | Shengmai San and Liuwei Dihuang Pills+Conventional Western Medicine Treatment | 1 dose, 2/d | Conventional Western Medicine Treatment | NM | 3 months | FBG, 2hPBG, HbA1c, fasting C-peptide | NM |
| Ma 2017 (1) | Elderly T2DM (60-80 years old) | 70.87±8.35 years old | 32/32 | Shenqi Maiwei Dihuang Decoction+Metformin+Insulin | 150ml, 2/d | Metformin+Insulin | Metformin: 0.25g, 3/d; Insulin: add based on blood sugar levels | 12 weeks | FBG, 2hPBG, TC, TG | NM |
| Su 2020 (1) | Elderly T2DM (65-82 years old) | control group: 71.23±4.35 years old; intervention group: 72.15±5.13 years old | 46/46 | Yangyin Xiaoke Decoction+Insulin | 1 dose, 2/d | Insulin | adjust dosage based on blood sugar levels, 3/d | 3 months | FBG, 2hPBG | NM |
| Li 2011 | Elderly T2DM (60-82 years old) | control group: 68±10.93 years old; intervention group: 73.94±6.06 years old | 68/56 | Yiqi Bushen Huoxue Recipe+Conventional Western Medicine Treatment | 1 dose, 2/d | Conventional Western Medicine Treatment | NM | 3 months | FBG^*^, 2hPBG^*^, HbA1c^*^ | NM |
| DAI 2020 | Elderly T2DM | control group: 67.36±9.28 years old; intervention group: 69.65±9.08 years old | 49/49 | Yiqi Yangyin Bushen Recipe+Acarbose | 100ml, 2/d | Acarbose | 0.5g, 3/d | 3 months | FBG, 2hPBG, HbA1c | control group: gastrointestinal discomfort (1), dizzy (2), palpitate (1); intervention group: gastrointestinal discomfort (2), dizzy (2), palpitate (1) |
| Zhao 2016 (1) | Elderly T2DM (60-82 years old) | control group: 69.3±7.8 years old; intervention group: 68.4±8.7 years old | 40/40 | Yuye Decoction+Repaglinide | 150ml, 2/d | Repaglinide | 1mg, 3/d | 3 months | FBG, OGTT, HbA1c, FINS, HOMA-IR, HOMA-β^*^ | NM |
| Traditional Chinese patent medicines | | | | | | | | | | |
| Wu 2012 | Elderly T2DM (60-80 years old) | control group: 63.5±6.5 years old; intervention group: 65.5±7.5 years old | 34/34 | Shenqi Jiangtang Granules+Acarbose | 2g, 3/d | Acarbose | 50mg, 3/d | 4 months | FBG, 2hPBG | NM |
| Liu 2018 | Elderly T2DM (60-78 years old) | control group: 66.8±10.2 years old; intervention group: 67.2±9.4 years old | 30/30 | Shenqi Jiangtang Granules+Metformin+Gliquidone | 1g, 3/d | Metformin+Gliquidone | Metformin: 0.5g, 2/d; Gliquidone: 30-60mg, 1/d | 12 weeks | FBG, 2hPBG, HbA1c, TC, TG, HDL^*^, LDL | no adverse reaction |
| Xia 2016 | Elderly T2DM of Qi-Yin Deficiency (≥60 years old) | control group: 73.62±6.54 years old; intervention group: 72.98±5.61 years old | 60/60 | Shenqi Jiangtang Granules+Acarbose | 1g, 3/d | Acarbose | 50-100mg, 3/d | 4 months | FBG, 2hPBG, HbA1c, IL-6, TNF-α, hs-CRP | control group: vomit (1), diarrhea (2); intervention group: vomit (2), diarrhea (3) |
| Bao 2019 | Elderly T2DM with deficiency of both qi and yin and blood stasis syndrome (≥60 years old) | control group: 68.58±5.49 years old; intervention group: 67.56±5.23 years old | 30/30 | Danzhi Jiangtang Capsules+Conventional Western Medicine Treatment | 5 capsules, 3/d | Conventional Western Medicine Treatment | NM | 3 months | FBG, HbA1c, DSQL | NM |
| Niu 2008 | Elderly T2DM (60-72 years old) | control group: 59.3±7.9 years old; intervention group: 58.4±8.7 years old | 32/28 | Danzhi Jiangtang Capsules+Gliclazide | 2g, 3/d | Gliclazide | 80-120mg, 3/d | 12 weeks | FBG^*^, 2hPBG^*^, HbA1c^*^, FINS, HOMA-IS, HOMA-IR | control group: hypoglycemia (3), abnormal liver function (1), decreased WBC (2); intervention group: hypoglycemia (2), decreased PLT (1) |
| Deng 2015 | Elderly T2DM (60-85 years old) | 71.29±3.85 years old | 49/49 | Jinkui Shenqi Pills+Metformin | 5g, 3/d | Metformin | 500mg, 3/d | 30 weeks | FBG, 2hPBG, HbA1c, FINS | control group: cardiovascular disease (6); intervention group: cardiovascular disease (1) |
| Cheng 2020 | Elderly T2DM of deficiency of Qi and Yin syndrome (60-80 years old) | control group: 68.95±6.43 years old; intervention group: 69.11±6.52 years old | 73/73 | Shiwei Yuquan Tablets+Gliquidone | 1.2g, 4/d | Gliquidone | 30mg, 3/d | 3 months | FBG, 2hPBG, HbA1c, TC, TG, FINS, HOMA-IR, 25(OH)D, TGF-β1, MMP | no adverse reaction |
| Xiao 2021 | Elderly T2DM (60-80 years old) | control group: 69.7±5.5 years old; intervention group: 68.9±5.2 years old | 49/49 | Yuquan Pills+Shagliptin | 6g, 4/d | Shagliptin | 5mg, 1/d | 12 weeks | FBG, 2hPBG, HbA1c, TC, TG, CRP, PV, EDI, HOMA-IR, HOMA-β, HOMA-IS, TNF-α, IL-18, NEUT#, LYM# | control group: headache (2); intervention group: headache (1), abdominal distension (1), diarrhea (1) |
| Li 2023 | Elderly T2DM (≥60 years old) | control group: 69.30±4.86 years old; intervention group: 69.13±5.22 years old | 40/40 | Liuwei Dihuang Pills^†^+Metformin | 8 pills, 3/d | Metformin | 0.5-1.0g, 2/d | 12 weeks | FBG, 2hPBG, HbA1c, FINS^*^, HOMA-IR, HOMA-β^*^, HOMA-IS, TNF-α^*^, IL-6, IL-8 | control group: drowsiness (2), nausea (1), loss of appetite (1); intervention group: drowsiness (2), nausea (0), loss of appetite (1) |
| Xiao 2018 | Elderly T2DM (65-88 years old) | control group: 66.23±5.89 years old; intervention group: 66.45±6.01 years old | 34/34 | Xiaoke Pills^†^+Acarbose | 5-10pills, 3/d | Acarbose | 25mg, 3/d | 3 months | FBG, 2hPBG, HbA1c, SF-36 | NM |
| Li 2022 (1) | Elderly T2DM (61-89 years old) | control group: 75.58±6.37 years old; intervention group: 76.08±6.42 years old | 50/50 | Shenqi Jiangtang Tablets^†^+Insulin Glargine Injection | 10.5g, 3/d | Insulin glargine | 0.2IU/kg, 1/d | 3 months | FBG, 2hPBG, HbA1c, TC, TG, HDL, LDL, HOMA-IR, HOMA-β, 2h-postprandial c-peptide, 2h-postprandial insulin, CRP | control group: vomit (1), gastrointestinal discomfort (1), hypoglycemia (1); intervention group: vomit (1), gastrointestinal discomfort (1), hypoglycemia (0) |
| Yu 2007 | Elderly T2DM (60-83 years old) | control group: 64.1 years old; intervention group: 65.5 years old | 32/32 | Jinqi Jiangtang Tablets^†^+ Glipizide sustained release tablets | 6-10 tablets, 3/d | Glipizide sustained release tablets | 5-15mg, 1/d | 12 weeks | FBG, 2hPBG, HbA1c, TC, TG, FINS, ALT^*^, Cr^*^, urine-ALB | no adverse reaction |
| Han 2023 (2) | Elderly T2DM (61-78 years old) | control group: 69.6±2.7 years old; intervention group: 69.3±2.5 years old | 42/38 | Jinlida Granules^†^+Dapagliflozin | 9g, 3/d | Dapagliflozin | 10mg, 1/d | 3 months | FBG, 2hPBG, HbA1c, HOMA-IR, HOMA-β, SOD, MDA, CRP | control group: vomit (0), infect (1), nausea (0); intervention group: vomit (1), infect (0), nausea (1) |
| Jiang 2020 | Elderly T2DM (60-75 years old) | control group: 66.8±3.7 years old; intervention group: 67.3±4.1 years old | 42/42 | Jinlida Granules^†^+Dapagliflozin | 1 dose, 3/d | Dapagliflozin | 10mg, 1/d | 12 weeks | FBG, 2hPBG, HbA1c, FINS, HOMA-IR, HOMA-β, BMI, NEUT#, LYM#, NLR, CRP, Amyloid protein A, SOD, MDA | control group: genital fungal infection (1); intervention group: nausea (1), hypotension (1) |
| Hu 2014 | Elderly T2DM (60-80 years old) | control group: 68.7 ± 3.2 years old; intervention group: 67.8 ±3.6 years old | 30/30 | Jinlida Granules^†^+Gliclazide | 9g, 3/d | Gliclazide | 30-60mg, 1/d | 12 weeks | FBG, 2hPBG, HbA1c, FINS, HOMA-IR, HOMA-β | NM |
| Wu 2020 | Elderly T2DM | control group: 70.19±10.02 years old; intervention group: 70.25±10.05 years old | 45/45 | Liuwei Dihuang Pills^†^+Metformin | 8 pills, 3/d | Metformin | 0.5g, 2/d | 3 months | FBG, 2hPBG, HbA1c, TC, TG, HDL, LDL | NM |
| Hou 2019 | Elderly T2DM (60-85 years old) | control group: 69.14± 5.68 years old; intervention group: 67.98 ± 5.54 years old | 60/60 | Maiwei Dihuang Pills^†^+Metformin+Pioglitazone Hydrochloride | 6g, 2/d | Metformin+Pioglitazone Hydrochloride | Metformin: 0.5g, 2/d-2g/d, 3/d; Pioglitazone Hydrochloride: 15-45mg, 1/d | 12 weeks | FBG^*^, 2hPBG^*^, HbA1c, FINS, HOMA-IR, Adiponectin, TC, TG, LDL, HDL, SOD, GSH-Px, MDA, AOPP, IgA, IgG^*^, IgM^*^, Complement C3, Complement C4^*^ | NM |
| Zhang 2003 (1) | Elderly T2DM (60-91 years old) | control group: 70 years old; intervention group: 73 years old | 32/30 | Jinqi Jiangtang Tablets^†^+Repaglinide | NovoNorm: 0.5-1.0mg, 1/d; Jinqi Jiangtang Tablets: 7-10 tablets, 3/d | Repaglinide | 5-10mg, 3/d | 12 weeks | FBG, 2hPBG, HbA1c, FINS^*^, ALT^*^, Cr^*^, BUN^*^, TC^*^, TG | control group: hypoglycemia (4); intervention group: hypoglycemia (1), abdominal pain (1) |
| Zhang 2003 (2) | Elderly T2DM (60-91 years old) | control group: 70 years old; intervention group: 73 years old | 32/30 | Jinqi Jiangtang Tablets^†^+Repaglinide | NovoNorm: 0.5-1.0mg, 1/d; Jinqi Jiangtang Tablets: 7-10 tablets, 3/d | Repaglinide | 5-10mg, 3/d | 12 weeks | FBG, 2hPBG, HbA1c, FINS^*^, ALT^*^, Cr^*^, BUN^*^, TC^*^, TG | control group: hypoglycemia (4); intervention group: hypoglycemia (1), abdominal pain (1) |
| Dai 2005 | Elderly T2DM with deficiency of liver and kidney yin syndrome (61-76 years old) | 66.8±4.6 years old | 54/44 | Qiju Dihuang Pills^†^+Glipizide | 8 pills, 3/d | Glipizide | 5-30mg/d, 3/d | 3 months | FBG^*^, 2hPBG, TC, TG | NM |
| Wang 2012 (1) | Elderly T2DM (65-85 years old) | 67.3±6.7 years old | 50/50 | Xiaoke Pills^†^+Intensive insulin pump therapy | 5-10 pills, 3/d | Intensive insulin pump therapy | Adjust dosage based on blood sugar levels | 3 months | FBG, 2hPBG, HbA1c | NM |
| Zhong 2017 | Elderly T2DM (55-78 years old) | control group:68.58±9.64 years old; intervention group: 66.35±11.32 years old | 36/32 | Xiaoke pills^†^+Metformin | Metformin: 0.25-0.5g, 3/d; Xiaoke pill: 5-10 pills, 3/d | Metformin+Glibenclamide | Metformin: 0.25-0.5g, 3/d; Glibenclamide: 2.5mg, 3/d | 3 months | FBG, 2hPBG, HbA1c, BMI^*^ | control group: hypoglycemia (2); intervention group: hypoglycemia (0) |
| Zhao 2021 | Elderly T2DM (60-79 years old) | control group: 69.04±9.01 years old; intervention group: 68.93±5.68 years old | 60/60 | Xuefu Zhuyu Pills^†^+Conventional Western Medicine Treatment | 8g, 2/d | Conventional Western Medicine Treatment | NM | 3 months | FBG, 2hPBG, HbA1c, fibrinogen | NM |
| Liu 2008 | Elderly T2DM (≥60 years old) | control group: 64±3.2 years old; intervention group: 63±2.7 years old | 36/36 | Xuezhikang Capsules^†^+Conventional Western Medicine Treatment | 0.6g, 2/d | Conventional Western Medicine Treatment+Fluvastatin | Fluvastatin: 40mg, 1/d | 12 weeks | TC, TG, HDL^*^, LDL, hs-CRP, FIB^*^, PT^*^, APTT^*^ | control group: nausea (1), headache (1); intervention group: diarrhea (1) |
| Zhao 2016 (2) | Elderly T2DM with deficiency of kidney yin syndrome (65-82 years old) | control group: 71.39±12.14 years old; intervention group: 69.85±10.57 years old | 50/50 | Liuwei Dihuang Pills^†^+OADs | 8 pills, 3/d | OADs | NM | 3 months | FBG, 2hPBG, HbA1c, FINS, HOMA-IR, ISI | no adverse reaction |
| Traditional Chinese Medicine Extracts | | | | | | | | | | |
| Cheng 2023 | Elderly T2DM (60-75 years old) | control group: 64.59±6.17 years old; intervention group: 65.36±5.23 years old | 36/36 | Corn silk aqueous extract | 2.4g, 2/d | placebo | 2.4g, 2/d | 3 months | FBG, 2hPBG, HbA1c, TC, TG^*^, HDL^*^, LDL^*^, ALT, AST, ALB^*^, Tbil^*^, Dbil, Ibil^*^, BUN^*^, Cr^*^, UA^*^, HOMA-IR, HOMA-β, HOMA-IS | NM |

^1^ Ages were displayed as mean ± standard deviation or mean.

^2^ “^†^” indicated that it was included in Pharmacopoeia of the People's Republic of China 2020.

^3^ “*” showed no significant difference between the intervention group and the control group.

Abbreviation: NM: Not mentioned, FBG: Fasting blood glucose, 2hPBG: 2-hour Postprandial blood glucose, HbA1c: Glycosylated hemoglobin, FINS: Fasting insulin, HOMA: Homeostasis model assessment, Lp (a): Lipoprotein(a), CRP: C-reaction protein, SOD: Superoxide Dismutase, MDA: Malondialdehyde, TC: Cholesterol, TG: Triglyceride, LDL: Low density lipoprotein, HDL: High density lipoprotein, ALT: Alanine aminotransferase, AST: Aspartate aminotransferase, ALB: Albumin, TBil: Total bilirubin, Dbil: Direct bilirubin, Ibil: Indirect bilirubin, BUN: Blood urea nitrogen, Cr: Creatinine, UA: Uric acid, IL: Interleukin, DSQL: Diabetes specific Quality of Life scale, PV: Plasma viscosity, EDI: Erythrocyte deformation index, TNF: Tumor necrosis factor, NEUT#: Neutrophil, LYM#: Lymphocyte, ApoB: Apolipoprotein b, ISI: Insulin sensitivity index, NLR: Neutrophil to lymphocyte ratio, TGF-β1: Transforming growth factor-β, MMP: Matrix metalloproteinases, GSH-Px: Glutathione peroxidase, AOPP: Advanced oxidation protein products, IgA: Immunoglobulin A, IgG: Immunoglobulin G, CAT: Catalase, SF-36: The medical outcomes study 36-item short from health survey, SBP: Systolic pressure, DBP: Diastolic pressure, BMI: Body Mass Index, OGTT: Oral glucose tolerance test, β2-MG: β2-Microglobulin, APTT: Activated partial thromboplastin time, PT: Prothrombin time, TT: Thrombin time, FIB: Fibrinogen, OADs: Oral antidiabetic agents.
