# Supplementary material for: Mechanism of traditional Chinese medicine in elderly diabetes mellitus and a systematic review of its clinical application
Source: Front Pharmacol. 2024 Mar 6;15:1339148. doi: 10.3389/fphar.2024.1339148 (PMC10953506; doi:10.3389/fphar.2024.1339148)
Supplement: Supplementary file 2 [file DataSheet1.zip › Supplementary Table S1-17/Supplementary Table S10.docx]

Supplementary Table S10 | Interventional drugs composition of TCM for elderly DCM.

| Study | Interventional drugs composition |
| --- | --- |
| Traditional Chinese Prescription | |
| Xing 2023 | Yangyin Yiqi Huoxue Recipe: Astragalus mongholicus Bunge [Fabaceae, Astragali radix] 20g, Poria cocos (Schw.)Wolf Poria [Polyporaceae, Poria] 15g, Codonopsis pilosula (Franch.) Nannf. [Campanulaceae, Codonopsis radix] 12g, Atractylodes macrocephala Koidz. [Asteraceae, Atractylodis macrocephalae rhizoma] 12g, Ophiopogon japonicus (Thunb.) Ker Gawl. [Asparagaceae, Ophiopogonis radix] 15g, Cornus officinalis Siebold & Zucc. [Cornaceae, Corni fructus] 12g, Schisandra chinensis (Turcz.) Baill. [Schisandraceae, Schisandrae chinensis fructus] 9g, Angelica sinensis (Oliv.) Diels [Apiaceae, Angelicae sinensis radix] 12g, Salvia miltiorrhiza Bunge [Lamiaceae, Salviae miltiorrhizae radix et rhizoma] 15g, Conioselinum anthriscoides 'Chuanxiong' [Apiaceae, Chuanxiong rhizoma] 12g, Pueraria montana var. lobata (Willd.) Maesen & S.M.Almeida ex Sanjappa & Predeep [Fabaceae, Puerariae lobatae radix] 15g, Allium chinense G.Don [Amaryllidaceae, Allii macrostemonis bulbus] 10g, Dolomiaea costus (Falc.) Kasana & A.K.Pandey [Asteraceae, Aucklandiae radix] 12g, Glycyrrhiza glabra L. [Fabaceae, Glycyrrhizae radix et rhizoma] 10g |
| Chen 2021 | Zhigancao Decoction: Glycyrrhiza uralensis Fisch. ex DC. [Fabaceae, Glycyrrhizae radix et rhizoma praeparata cum melle] 12g, Rehmannia glutinosa (Gaertn.) DC. [Orobanchaceae, Rehmanniae Radix] 20g, Panax ginseng C.A.Mey. [Araliaceae, Ginseng radix et rhizoma] 15g, Neolitsea cassia (L.) Kosterm. [Lauraceae, Cinnamomi ramulus] 12g, Equus asinus L. [Equidae, Asini corii colla] 5g, Ophiopogon japonicus (Thunb.) Ker Gawl. [Asparagaceae, Ophiopogonis radix] 9g, Cannabis sativa L. [Cannabaceae, Cannabis fructus] 10g, Zingiber officinale Roscoe [Zingiberaceae, Zingiberis rhizoma recens] 5 pieces, Ziziphus jujuba Mill. [Rhamnaceae, Jujubae fructus] 10 pieces |
