# Supplementary material for: Mechanism of traditional Chinese medicine in elderly diabetes mellitus and a systematic review of its clinical application
Source: Front Pharmacol. 2024 Mar 6;15:1339148. doi: 10.3389/fphar.2024.1339148 (PMC10953506; doi:10.3389/fphar.2024.1339148)
Supplement: Supplementary file 2 [file DataSheet1.zip › Supplementary Table S1-17/Supplementary Table S11.docx]

Supplementary Table S11 | Interventional drugs composition of TCM for elderly DOP.

| Study | Interventional drugs composition |
| --- | --- |
| Traditional Chinese Prescription | |
| Liu 2018 | Bushen Huoxue Prescription: Ligustrum lucidum W.T.Aiton [Oleaceae, Ligustri lucidi fructus] 15g, Eclipta prostrata (L.) L. [Asteraceae, Ecliptae herba] 15g, Curculigo orchioides Gaertn. [Hypoxidaceae, Curculiginis rhizoma] 10g, Epimedium sagittatum (Siebold & Zucc.) Maxim. [Berberidaceae, Epimedii folium] 10g, Lycium barbarum L. [Solanaceae, Lycii fructus] 15g, Cuscuta chinensis Lam. [Convolvulaceae, Cuscutae semen] 15g, Eucommia ulmoides Oliv. [Eucommiaceae, Eucommiae cortex] 10g, Dipsacus asper Wall. ex DC. [Caprifoliaceae, Dipsaci radix] 10g, Morindae officinalis radix [Rubiaceae, Morindae officinalis radix] 10g, Angelica sinensis (Oliv.) Diels [Apiaceae, Angelicae sinensis radix] 10g, Salvia miltiorrhiza Bunge [Lamiaceae, Salviae miltiorrhizae radix et rhizoma] 15g, Drynaria roosii Nakaike [Polypodiaceae, Drynariae rhizoma] 12g |
| Li 2018 (2) | Bushen Yigu Recipe: Astragalus mongholicus Bunge [Fabaceae, Astragali radix] 30g, Conioselinum anthriscoides 'Chuanxiong' [Apiaceae, Chuanxiong rhizoma] 10g, Rehmannia glutinosa (Gaertn.) DC. [Orobanchaceae, Rehmanniae radix praeparata] 20g, Atractylodes macrocephala Koidz. [Asteraceae, Atractylodis macrocephalae rhizoma] 20g, Angelica sinensis (Oliv.) Diels [Apiaceae, Angelicae sinensis radix] 20g, Drynaria roosii Nakaike [Polypodiaceae, Drynariae rhizoma] 20g, Cullen corylifolium (L.) Medik. [Fabaceae, Psoraleae fructus] 30g, Epimedium sagittatum (Siebold & Zucc.) Maxim. [Berberidaceae, Epimedii folium] 20g, Achyranthes bidentata Blume [Amaranthaceae, Achyranthis bidentatae radix] 15g, Lycium barbarum L. [Solanaceae, Lycii fructus] 15g, Cornus officinalis Siebold & Zucc. [Cornaceae, Corni fructus] 20g, Taxillus chinensis (DC.) Danser [Loranthaceae, Taxilli herba] 20g, Morus alba L. [Moraceae, mulberry] 15g |
| Lin 2021 | Bushen Zhuanggu Prescription: Epimedium sagittatum (Siebold & Zucc.) Maxim. [Berberidaceae, Epimedii folium] 20g, Drynaria roosii Nakaike [Polypodiaceae, Drynariae rhizoma] 15g, Dipsacus asper Wall. ex DC. [Caprifoliaceae, Dipsaci radix] 10g, Achyranthes bidentata Blume [Amaranthaceae, Achyranthis bidentatae radix] 10g, Rehmannia glutinosa (Gaertn.) DC. [Orobanchaceae, Rehmanniae radix praeparata] 15g, Angelica sinensis (Oliv.) Diels [Apiaceae, Angelicae sinensis radix] 20g, Paeonia lactiflora Pall. [Paeoniaceae, Paeoniae radix alba] 10g, Eucommia ulmoides Oliv. [Eucommiaceae, Eucommiae cortex] 15g, Spatholobus suberectus Dunn [Fabaceae, Spatholobi caulis] 10g, Corydalis yanhusuo (Y.H.Chou & Chun C.Hsu) W.T.Wang ex Z.Y.Su & C.Y.Wu [Papaveraceae, Corydalis rhizoma] 10g, Glycyrrhiza glabra L. [Fabaceae, Glycyrrhizae radix et rhizoma]9g |
| Zong 2017 | Qishu Tanggu Decoction: Astragalus mongholicus Bunge [Fabaceae, Astragali radix] 30g, Dioscorea oppositifolia L. [Dioscoreaceae, Dioscoreae rhizoma] 30g, Atractylodes macrocephala Koidz. [Asteraceae, Atractylodis macrocephalae rhizoma] 10g, Poria cocos(Schw.)Wolf Poria [Polyporaceae, Poria] 12g, Dolomiaea costus (Falc.) Kasana & A.K.Pandey [Asteraceae, Aucklandiae radix] 10g, Citrus × limon (L.) Osbeck [Rutaceae, Citri sarcodactylis fructus] 10g, Lycium barbarum L. [Solanaceae, Lycii fructus] 10g, Salvia miltiorrhiza Bunge [Lamiaceae, Salviae miltiorrhizae radix et rhizoma] 20g, Lablab purpureus subsp. purpureus [Fabaceae, Lablab semen album] 12g, Coix lacryma-jobi var. ma-yuen (Rom.Caill.) Stapf [Poaceae, Coicis semen] 30g, Angelica sinensis (Oliv.) Diels [Apiaceae, Angelicae sinensis radix] 10g, Corydalis yanhusuo (Y.H.Chou & Chun C.Hsu) W.T.Wang ex Z.Y.Su & C.Y.Wu [Papaveraceae, Corydalis rhizoma] 10g, Whitmania pigra Whitman [Hirudinidae, Hirudo] 10g, Cullen corylifolium (L.) Medik. [Fabaceae, Psoraleae fructus] 10g, Cervi cornus colla 10g, Pheretima aspergillum (E.Perrier) [Megascolecidae, Pheretima] 10g, Epimedium sagittatum (Siebold & Zucc.) Maxim. [Berberidaceae, Epimedii folium] 10g, Rehmannia glutinosa (Gaertn.) DC. [Orobanchaceae, Rehmanniae radix praeparata] 20g, Cornus officinalis Siebold & Zucc. [Cornaceae, Corni fructus] 12g, Cistanche deserticola Ma [Orobanchaceae, Cistanches herba] 10g, Zingiber kawagoi Hayata [Zingiberaceae] 10g, Prunus persica (L.) Batsch [Rosaceae, Persicae semen] 10g, Carthamus tinctorius L. [Asteraceae, Carthami flos] 10g |
| Zhang 2019 | ShenTong ZhuYu Decoction: Angelica sinensis (Oliv.) Diels [Apiaceae, Angelicae sinensis radix] 9g, Carthamus tinctorius L. [Asteraceae, Carthami flos] 9g, Prunus persica (L.) Batsch [Rosaceae, Persicae semen] 9g, Achyranthes bidentata Blume [Amaranthaceae, Achyranthis bidentatae radix] 9g, Conioselinum anthriscoides 'Chuanxiong' [Apiaceae, Chuanxiong rhizoma] 6g, Glycyrrhiza glabra L. [Fabaceae, Glycyrrhizae radix et rhizoma] 6g, Faeces Trogopteri 6g, Commiphora myrrha (T.Nees) Engl. [Burseraceae, Myrrha] 6g, Pheretima aspergillum (E.Perrier) [Megascolecidae, Pheretima] 6g, Gentiana macrophylla Pall. [Gentianaceae, Gentianae macrophyllae radix] 3g, Cyperus rotundus L. [Cyperaceae, Cyperi rhizoma] 3g, Hansenia weberbaueriana (Fedde ex H.Wolff) Pimenov & Kljuykov [Apiaceae, Notopterygii rhizoma et radix] 3g |
| Xiao 2020 | Tonifying Kidney and Strengthening Bone Prescription: Epimedium sagittatum (Siebold & Zucc.) Maxim. [Berberidaceae, Epimedii folium] 20g, Drynaria roosii Nakaike [Polypodiaceae, Drynariae rhizoma] 15g, Dipsacus asper Wall. ex DC. [Caprifoliaceae, Dipsaci radix] 10g, Cullen corylifolium (L.) Medik. [Fabaceae, Psoraleae fructus] 10g, Salvia miltiorrhiza Bunge [Lamiaceae, Salviae miltiorrhizae radix et rhizoma] 10g, Astragalus mongholicus Bunge [Fabaceae, Astragali radix] 15g, Carthamus tinctorius L. [Asteraceae, Carthami flos] 12g, Rehmannia glutinosa (Gaertn.) DC. [Orobanchaceae, Rehmanniae radix praeparata] 15g, Angelica sinensis (Oliv.) Diels [Apiaceae, Angelicae sinensis radix] 20g, Dioscorea oppositifolia L. [Dioscoreaceae, Dioscoreae rhizoma] 15g, Lilium lancifolium Thunb. [Liliaceae, Lilii bulbus] 20g, Atractylodes macrocephala Koidz. [Asteraceae, Atractylodis macrocephalae rhizoma] 20g, Spatholobus suberectus Dunn [Fabaceae, Spatholobi caulis] 10g, Glycyrrhiza glabra L. [Fabaceae, Glycyrrhizae radix et rhizoma]9g |
| Li 2015 | Traditional Chinese medicine formulas: Salvia miltiorrhiza Bunge [Lamiaceae, Salviae miltiorrhizae radix et rhizoma] 10g, Astragalus mongholicus Bunge [Fabaceae, Astragali radix] 10g, Cullen corylifolium (L.) Medik. [Fabaceae, Psoraleae fructus] 20g, Taxillus chinensis (DC.) Danser [Loranthaceae, Taxilli herba] 20g, Epimedium sagittatum (Siebold & Zucc.) Maxim. [Berberidaceae, Epimedii folium] 20g, Eucommia ulmoides Oliv. [Eucommiaceae, Eucommiae cortex] 20g |
| Yang 2021 (2) | Zishen Jiangtang Pills: Astragalus mongholicus Bunge [Fabaceae, Astragali radix], Rehmannia glutinosa (Gaertn.) DC. [Orobanchaceae, Rehmanniae Radix], Rehmannia glutinosa (Gaertn.) DC. [Orobanchaceae, Rehmanniae radix praeparata], Schisandra chinensis (Turcz.) Baill. [Schisandraceae, Schisandrae chinensis fructus], Chinemys reevesii (Gray) [testudinidae, Testudinis carapax et plastrum], Trionyx sinensis Wiegmann [Trionychidae, Trionycis carapax], Cibotium barometz (L.) J.Sm. [Cyatheaceae, Cibotii rhizoma], Epimedium sagittatum (Siebold & Zucc.) Maxim. [Berberidaceae, Epimedii folium] |
| Hu 2017 | Zuogui Pills: Rehmannia glutinosa (Gaertn.) DC. [Orobanchaceae, Rehmanniae radix praeparata], Dioscorea oppositifolia L. [Dioscoreaceae, Dioscoreae rhizoma], Lycium barbarum L. [Solanaceae, Lycii fructus], Testudinis carapacis et plastri colla, Cervi cornus colla, Cuscuta chinensis Lam. [Convolvulaceae, Cuscutae semen], Cornus officinalis Siebold & Zucc. [Cornaceae, Corni fructus], Achyranthes bidentata Blume [Amaranthaceae, Achyranthis bidentatae radix] |
| Traditional Chinese patent medicines | |
| Yu 2016 | Jintiange Capsules: Artificial tiger bone powder |
| Song 2016 | Jintiange Capsules: Artificial tiger bone powder |
| Gao 2012 | Tangmaikang Granules: Astragalus mongholicus Bunge [Fabaceae, Astragali radix] 240g, Rehmannia glutinosa (Gaertn.) DC. [Orobanchaceae, Rehmanniae Radix] 260g, Paeonia lactiflora Pall. [Paeoniaceae, Paeoniae radix rubra] 260g, Salvia miltiorrhiza Bunge [Lamiaceae, Salviae miltiorrhizae radix et rhizoma] 240g, Achyranthes bidentata Blume [Amaranthaceae, Achyranthis bidentatae radix] 150g, Ophiopogon japonicus (Thunb.) Ker Gawl. [Asparagaceae, Ophiopogonis radix] 150g, Pueraria montana var. lobata (Willd.) Maesen & S.M.Almeida ex Sanjappa & Predeep [Fabaceae, Puerariae lobatae radix] 150g, Morus alba L. [Moraceae, Mori folium] 150g, Coptis chinensis Franch. [Ranunculaceae, Coptidis rhizoma] 50g, Polygonatum sibiricum Redouté [Asparagaceae, Polygonati rhizoma] 150g, Epimedium sagittatum (Siebold & Zucc.) Maxim. [Berberidaceae, Epimedii folium] 200g |
| Traditional Chinese Medicine Extracts | |
| Luo 2014 | Qianggu Capsules: Total flavonoids of Drynaria roosii Nakaike [Polypodiaceae, Drynariae rhizoma] |
