# Supplementary material for: Mechanism of traditional Chinese medicine in elderly diabetes mellitus and a systematic review of its clinical application
Source: Front Pharmacol. 2024 Mar 6;15:1339148. doi: 10.3389/fphar.2024.1339148 (PMC10953506; doi:10.3389/fphar.2024.1339148)
Supplement: Supplementary file 2 [file DataSheet1.zip › Supplementary Table S1-17/Supplementary Table S12.docx]

Supplementary Table S12 | Frequency of Traditional Chinese Medicine for the treatment of elderly DOP in Traditional Chinese Prescription.

| Traditional Chinese Medicine | Frequency |
| --- | --- |
| Epimedium sagittatum (Siebold & Zucc.) Maxim. [Berberidaceae, Epimedii folium] | 7 |
| Angelica sinensis (Oliv.) Diels [Apiaceae, Angelicae sinensis radix] | 6 |
| Rehmannia glutinosa (Gaertn.) DC. [Orobanchaceae, Rehmanniae radix praeparata] | 6 |
| Astragalus mongholicus Bunge [Fabaceae, Astragali radix] | 5 |
| Achyranthes bidentata Blume [Amaranthaceae, Achyranthis bidentatae radix] | 4 |
| Cullen corylifolium (L.) Medik. [Fabaceae, Psoraleae fructus] | 4 |
| Drynaria roosii Nakaike [Polypodiaceae, Drynariae rhizoma] | 4 |
| Lycium barbarum L. [Solanaceae, Lycii fructus] | 4 |
| Salvia miltiorrhiza Bunge [Lamiaceae, Salviae miltiorrhizae radix et rhizoma] | 4 |
| Atractylodes macrocephala Koidz. [Asteraceae, Atractylodis macrocephalae rhizoma] | 3 |
| Carthamus tinctorius L. [Asteraceae, Carthami flos] | 3 |
| Cornus officinalis Siebold & Zucc. [Cornaceae, Corni fructus] | 3 |
| Dioscorea oppositifolia L. [Dioscoreaceae, Dioscoreae rhizoma] | 3 |
| Dipsacus asper Wall. ex DC. [Caprifoliaceae, Dipsaci radix] | 3 |
| Eucommia ulmoides Oliv. [Eucommiaceae, Eucommiae cortex] | 3 |
| Glycyrrhiza glabra L. [Fabaceae, Glycyrrhizae radix et rhizoma] | 3 |
| Cervi cornus colla | 2 |
| Conioselinum anthriscoides 'Chuanxiong' [Apiaceae, Chuanxiong rhizoma] | 2 |
| Corydalis yanhusuo (Y.H.Chou & Chun C.Hsu) W.T.Wang ex Z.Y.Su & C.Y.Wu [Papaveraceae, Corydalis rhizoma] | 2 |
| Cuscuta chinensis Lam. [Convolvulaceae, Cuscutae semen] | 2 |
| Pheretima aspergillum (E.Perrier) [Megascolecidae, Pheretima] | 2 |
| Prunus persica (L.) Batsch [Rosaceae, Persicae semen] | 2 |
| Spatholobus suberectus Dunn [Fabaceae, Spatholobi caulis] | 2 |
| Taxillus chinensis (DC.) Danser [Loranthaceae, Taxilli herba] | 2 |
| Chinemys reevesii (Gray) [testudinidae, Testudinis carapax et plastrum] | 1 |
| Cibotium barometz (L.) J.Sm. [Cyatheaceae, Cibotii rhizoma] | 1 |
| Cistanche deserticola Ma [Orobanchaceae, Cistanches herba] | 1 |
| Citrus × limon (L.) Osbeck [Rutaceae, Citri sarcodactylis fructus] | 1 |
| Coix lacryma-jobi var. ma-yuen (Rom.Caill.) Stapf [Poaceae, Coicis semen] | 1 |
| Commiphora myrrha (T.Nees) Engl. [Burseraceae, Myrrha] | 1 |
| Curculigo orchioides Gaertn. [Hypoxidaceae, Curculiginis rhizoma] | 1 |
| Cyperus rotundus L. [Cyperaceae, Cyperi rhizoma] | 1 |
| Dolomiaea costus (Falc.) Kasana & A.K.Pandey [Asteraceae, Aucklandiae radix] | 1 |
| Eclipta prostrata (L.) L. [Asteraceae, Ecliptae herba] | 1 |
| Faeces Trogopteri | 1 |
| Gentiana macrophylla Pall. [Gentianaceae, Gentianae macrophyllae radix] | 1 |
| Hansenia weberbaueriana (Fedde ex H.Wolff) Pimenov & Kljuykov [Apiaceae, Notopterygii rhizoma et radix] | 1 |
| Lablab purpureus subsp. purpureus [Fabaceae, Lablab semen album] | 1 |
| Ligustrum lucidum W.T.Aiton [Oleaceae, Ligustri lucidi fructus] | 1 |
| Lilium lancifolium Thunb. [Liliaceae, Lilii bulbus] | 1 |
| Morindae officinalis radix [Rubiaceae, Morindae officinalis radix] | 1 |
| Morus alba L. [Moraceae, mulberry] | 1 |
| Paeonia lactiflora Pall. [Paeoniaceae, Paeoniae radix alba] | 1 |
| Poria cocos(Schw.)Wolf Poria [Polyporaceae, Poria] | 1 |
| Rehmannia glutinosa (Gaertn.) DC. [Orobanchaceae, Rehmanniae Radix] | 1 |
| Schisandra chinensis (Turcz.) Baill. [Schisandraceae, Schisandrae chinensis fructus] | 1 |
| Testudinis carapacis et plastri colla | 1 |
| Trionyx sinensis Wiegmann [Trionychidae, Trionycis carapax] | 1 |
| Whitmania pigra Whitman [Hirudinidae, Hirudo] | 1 |
| Zingiber kawagoi Hayata [Zingiberaceae] | 1 |
