# Supplementary material for: Mechanism of traditional Chinese medicine in elderly diabetes mellitus and a systematic review of its clinical application
Source: Front Pharmacol. 2024 Mar 6;15:1339148. doi: 10.3389/fphar.2024.1339148 (PMC10953506; doi:10.3389/fphar.2024.1339148)
Supplement: Supplementary file 2 [file DataSheet1.zip › Supplementary Table S1-17/Supplementary Table S13.docx]

Supplementary Table S13 | Interventional drugs composition of TCM for elderly diabetes with cognitive impairment.

| Study | Interventional drugs composition |
| --- | --- |
| Traditional Chinese Prescription | |
| Yu 2022 | Bushen Jianpi Huoxue Formula: Rehmannia glutinosa (Gaertn.) DC. [Orobanchaceae, Rehmanniae radix praeparata] 15g, Lycium barbarum L. [Solanaceae, Lycii fructus] 15g, Achyranthes bidentata Blume [Amaranthaceae, Achyranthis bidentatae radix] 15g, Astragalus mongholicus Bunge [Fabaceae, Astragali radix] 20g, Codonopsis pilosula (Franch.) Nannf. [Campanulaceae, Codonopsis radix] 15g, Atractylodes macrocephala Koidz. [Asteraceae, Atractylodis macrocephalae rhizoma] 15g, Angelica sinensis (Oliv.) Diels [Apiaceae, Angelicae sinensis radix] 15g, Prunus persica (L.) Batsch [Rosaceae, Persicae semen] 15g, Carthamus tinctorius L. [Asteraceae, Carthami flos] 12g, Acorus calamus var. angustatus Besser [Acoraceae, Acori tatarinowii rhizoma] 15g, Glycyrrhiza uralensis Fisch. ex DC. [Fabaceae, Glycyrrhizae radix et rhizoma praeparata cum melle] 6g |
| Zhao 2016 (4) | Chinese medicine for nourishing kidney，eliminating phlegm and damp: Panax ginseng C.A.Mey. [Araliaceae, Ginseng radix et rhizoma] 20g, Dioscorea oppositifolia L. [Dioscoreaceae, Dioscoreae rhizoma] 15g, Poria cocos (Schw.)Wolf Poria [Polyporaceae, Poria] 15g, Salvia miltiorrhiza Bunge [Lamiaceae, Salviae miltiorrhizae radix et rhizoma] 15g, Cistanche deserticola Ma [Orobanchaceae, Cistanches herba] 15g, Pinellia ternata (Thunb.) Makino [Araceae, Pinelliae rhizoma] 10g, Wurfbainia villosa (Lour.) Škorničk. & A.D.Poulsen [Zingiberaceae, Amomi fructus] 10g, Acorus calamus var. angustatus Besser [Acoraceae, Acori tatarinowii rhizoma] 8g, Glycyrrhiza glabra L. [Fabaceae, Glycyrrhizae radix et rhizoma] 8g |
| Zhao 2016 (5) | Tonifying Deficiency for Dispelling Turbidity and Removing Obstruction in Collaterals Method: Panax ginseng C.A.Mey. [Araliaceae, Ginseng radix et rhizoma], Cervi cornus colla, Astragalus mongholicus Bunge [Fabaceae, Astragali radix], Rehmannia glutinosa (Gaertn.) DC. [Orobanchaceae, Rehmanniae Radix], Panax notoginseng (Burkill) F.H.Chen [Araliaceae, Notoginseng radix et rhizoma], Paeonia × suffruticosa Andrews [Paeoniaceae, Moutan cortex], Acorus calamus var. angustatus Besser [Acoraceae, Acori tatarinowii rhizoma], Sinapis alba L. [Brassicaceae, Sinapis semen], Gynostemma pentaphyllum (Thunb.) Makino [Cucurbitaceae, Gynostemma pentaphyllum] |
| Liu 2016 | Tonifying Deficiency for Dispelling Turbidity and Removing Obstruction in Collaterals Method: Panax ginseng C.A.Mey. [Araliaceae, Ginseng radix et rhizoma], Cervi cornus colla, Astragalus mongholicus Bunge [Fabaceae, Astragali radix], Rehmannia glutinosa (Gaertn.) DC. [Orobanchaceae, Rehmanniae Radix], Panax notoginseng (Burkill) F.H.Chen [Araliaceae, Notoginseng radix et rhizoma], Paeonia × suffruticosa Andrews [Paeoniaceae, Moutan cortex], Acorus calamus var. angustatus Besser [Acoraceae, Acori tatarinowii rhizoma], Sinapis alba L. [Brassicaceae, Sinapis semen], Gynostemma pentaphyllum (Thunb.) Makino [Cucurbitaceae, Gynostemma pentaphyllum] |
| Yan 2019 | Yiqi bushen huoxue decoction: Panax ginseng C.A.Mey. [Araliaceae, Ginseng radix et rhizoma], Cervus nippon Temminck [Cervidae, Cervi cornu pantotrichum], Rehmannia glutinosa (Gaertn.) DC. [Orobanchaceae, Rehmanniae Radix], Panax notoginseng (Burkill) F.H.Chen [Araliaceae, Notoginseng radix et rhizoma], Paeonia × suffruticosa Andrews [Paeoniaceae, Moutan cortex], Acorus calamus var. angustatus Besser [Acoraceae, Acori tatarinowii rhizoma], Polygala tenuifolia Willd. [Polygalaceae, Polygalae radix] |
| Gao 2017 | Yizhi Heji: Rehmannia glutinosa (Gaertn.) DC. [Orobanchaceae, Rehmanniae radix praeparata] 30g, Morindae officinalis radix [Rubiaceae, Morindae officinalis radix] 30g, Codonopsis pilosula (Franch.) Nannf. [Campanulaceae, Codonopsis radix] 12g, Ophiopogon japonicus (Thunb.) Ker Gawl. [Asparagaceae, Ophiopogonis radix] 15g, Cuscuta chinensis Lam. [Convolvulaceae, Cuscutae semen] 30g, Ziziphus jujuba Mill. [Rhamnaceae, Ziziphi spinosae semen] 30g, Polygala tenuifolia Willd. [Polygalaceae, Polygalae radix] 6g, Bupleurum chinense DC. [Apiaceae, BUPLEURI RADIX] 3g, Paeonia lactiflora Pall. [Paeoniaceae, Paeoniae radix alba] 15g, Poria cocos (Schw.)Wolf Poria [Polyporaceae, Poria] 5g, Salvia miltiorrhiza Bunge [Lamiaceae, Salviae miltiorrhizae radix et rhizoma] 9g, Glycyrrhiza glabra L. [Fabaceae, Glycyrrhizae radix et rhizoma] 3g |
| Traditional Chinese patent medicines | |
| Guo 2020 | Jinlida Granules: Panax ginseng C.A.Mey. [Araliaceae, Ginseng radix et rhizoma] 184.5g, Polygonatum sibiricum Redouté [Asparagaceae, Polygonati rhizoma] 244.5g, Atractylodes lancea (Thunb.) DC. [Asteraceae, Atractylodis rhizoma] 122.2g, Sophora flavescens Aiton [Fabaceae, Sophorae flavescentis radix] 100g, Ophiopogon japonicus (Thunb.) Ker Gawl. [Asparagaceae, Ophiopogonis radix] 244.5g, Rehmannia glutinosa (Gaertn.) DC. [Orobanchaceae, Rehmanniae Radix] 184.5g, Reynoutria multiflora (Thunb.) Moldenke [Polygonaceae, Polygoni multiflori radix] 149g, Cornus officinalis Siebold & Zucc. [Cornaceae, Corni fructus] 244.5g, Poria cocos(Schw.)Wolf Poria [Polyporaceae, Poria] 149g, Eupatorium fortunei Turcz. [Asteraceae, Eupatorii herba] 100g, Coptis chinensis Franch. [Ranunculaceae, Coptidis rhizoma] 100g, Anemarrhena asphodeloides Bunge [Asparagaceae, Anemarrhenae rhizoma] 122.2g, Epimedium sagittatum (Siebold & Zucc.) Maxim. [Berberidaceae, Epimedii folium] 100g, Salvia miltiorrhiza Bunge [Lamiaceae, Salviae miltiorrhizae radix et rhizoma] 160g, Pueraria montana var. lobata (Willd.) Maesen & S.M.Almeida ex Sanjappa & Predeep [Fabaceae, Puerariae lobatae radix] 244.5g, Litchi chinensis Sonn. [Sapindaceae, Litchi semen] 244.5g, Lycium barbarum L. [Solanaceae, Lycii cortex] 149g |
| Zhao 2017 | Xiaoke Pills: Pueraria montana var. lobata (Willd.) Maesen & S.M.Almeida ex Sanjappa & Predeep [Fabaceae, Puerariae lobatae radix] 265g, Rehmannia glutinosa (Gaertn.) DC. [Orobanchaceae, Rehmanniae Radix] 159g, Astragalus mongholicus Bunge [Fabaceae, Astragali radix] 53g, Trichosanthes kirilowii Maxim. [Cucurbitaceae, Trichosanthis radix] 265g, Zea mays L. [Poaceae, corn silk] 265g, Schisandra chinensis (Turcz.) Baill. [Schisandraceae, Schisandrae chinensis fructus] 53g, Dioscorea oppositifolia L. [Dioscoreaceae, Dioscoreae rhizoma] 26.5g, Glibenclamide 0.25g |
| Traditional Chinese Medicine Extracts | |
| Wang 2012 (2) | Ginkgo biloba Tablets: Ginkgo biloba L. [Ginkgoaceae, Ginkgo leaves extract] 40g |
