# Supplementary material for: Mechanism of traditional Chinese medicine in elderly diabetes mellitus and a systematic review of its clinical application
Source: Front Pharmacol. 2024 Mar 6;15:1339148. doi: 10.3389/fphar.2024.1339148 (PMC10953506; doi:10.3389/fphar.2024.1339148)
Supplement: Supplementary file 2 [file DataSheet1.zip › Supplementary Table S1-17/Supplementary Table S14.docx]

Supplementary Table S14 | Frequency of Traditional Chinese Medicine for the treatment of elderly diabetes with cognitive impairment in Traditional Chinese Prescription.

| Traditional Chinese Medicine | Frequency |
| --- | --- |
| Rehmannia glutinosa (Gaertn.) DC. [Orobanchaceae, Rehmanniae Radix] | 5 |
| Acorus calamus var. angustatus Besser [Acoraceae, Acori tatarinowii rhizoma] | 4 |
| Panax ginseng C.A.Mey. [Araliaceae, Ginseng radix et rhizoma] | 4 |
| Astragalus mongholicus Bunge [Fabaceae, Astragali radix] | 4 |
| Paeonia × suffruticosa Andrews [Paeoniaceae, Moutan cortex] | 3 |
| Panax notoginseng (Burkill) F.H.Chen [Araliaceae, Notoginseng radix et rhizoma] | 3 |
| Gynostemma pentaphyllum (Thunb.) Makino [Cucurbitaceae, Gynostemma pentaphyllum] | 2 |
| Pueraria montana var. lobata (Willd.) Maesen & S.M.Almeida ex Sanjappa & Predeep [Fabaceae, Puerariae lobatae radix] | 2 |
| Sinapis alba L. [Brassicaceae, Sinapis semen] | 2 |
| Cervi cornus colla | 2 |
| Achyranthes bidentata Blume [Amaranthaceae, Achyranthis bidentatae radix] | 1 |
| Anemarrhena asphodeloides Bunge [Asparagaceae, Anemarrhenae rhizoma] | 1 |
| Angelica sinensis (Oliv.) Diels [Apiaceae, Angelicae sinensis radix] | 1 |
| Atractylodes lancea (Thunb.) DC. [Asteraceae, Atractylodis rhizoma] | 1 |
| Atractylodes macrocephala Koidz. [Asteraceae, Atractylodis macrocephalae rhizoma] | 1 |
| Carthamus tinctorius L. [Asteraceae, Carthami flos] | 1 |
| Cervus nippon Temminck [Cervidae, Cervi cornu pantotrichum] | 1 |
| Codonopsis pilosula (Franch.) Nannf. [Campanulaceae, Codonopsis radix] | 1 |
| Coptis chinensis Franch. [Ranunculaceae, Coptidis rhizoma] | 1 |
| Cornus officinalis Siebold & Zucc. [Cornaceae, Corni fructus] | 1 |
| Dioscorea oppositifolia L. [Dioscoreaceae, Dioscoreae rhizoma] | 1 |
| Epimedium sagittatum (Siebold & Zucc.) Maxim. [Berberidaceae, Epimedii folium] | 1 |
| Eupatorium fortunei Turcz. [Asteraceae, Eupatorii herba] | 1 |
| Glycyrrhiza uralensis Fisch. ex DC. [Fabaceae, Glycyrrhizae radix et rhizoma praeparata cum melle] | 1 |
| Litchi chinensis Sonn. [Sapindaceae, Litchi semen] | 1 |
| Lycium barbarum L. [Solanaceae, Lycii cortex] | 1 |
| Lycium barbarum L. [Solanaceae, Lycii fructus] | 1 |
| Ophiopogon japonicus (Thunb.) Ker Gawl. [Asparagaceae, Ophiopogonis radix] | 1 |
| Polygala tenuifolia Willd. [Polygalaceae, Polygalae radix] | 1 |
| Polygonatum sibiricum Redouté [Asparagaceae, Polygonati rhizoma] | 1 |
| Poria cocos(Schw.)Wolf Poria [Polyporaceae, Poria] | 1 |
| Prunus persica (L.) Batsch [Rosaceae, Persicae semen] | 1 |
| Rehmannia glutinosa (Gaertn.) DC. [Orobanchaceae, Rehmanniae radix praeparata] | 1 |
| Reynoutria multiflora (Thunb.) Moldenke [Polygonaceae, Polygoni multiflori radix] | 1 |
| Salvia miltiorrhiza Bunge [Lamiaceae, Salviae miltiorrhizae radix et rhizoma] | 1 |
| Schisandra chinensis (Turcz.) Baill. [Schisandraceae, Schisandrae chinensis fructus] | 1 |
| Sophora flavescens Aiton [Fabaceae, Sophorae flavescentis radix] | 1 |
| Trichosanthes kirilowii Maxim. [Cucurbitaceae, Trichosanthis radix] | 1 |
| Zea mays L. [Poaceae, corn silk] | 1 |
