# Supplementary material for: Mechanism of traditional Chinese medicine in elderly diabetes mellitus and a systematic review of its clinical application
Source: Front Pharmacol. 2024 Mar 6;15:1339148. doi: 10.3389/fphar.2024.1339148 (PMC10953506; doi:10.3389/fphar.2024.1339148)
Supplement: Supplementary file 2 [file DataSheet1.zip › Supplementary Table S1-17/Supplementary Table S15.docx]

Supplementary Table S15 | Interventional drugs composition of TCM for elderly diabetes with vascular injury.

| Study | Interventional drugs composition |
| --- | --- |
| Traditional Chinese Prescription | |
| Cheng 2019 | Modified Huangqi Guizhi Wuwu Tang: Astragalus mongholicus Bunge [Fabaceae, Astragali radix] 30g, Neolitsea cassia (L.) Kosterm. [Lauraceae, Cinnamomi ramulus] 10g, Paeonia lactiflora Pall. [Paeoniaceae, Paeoniae radix alba] 15g, Zingiber officinale Roscoe [Zingiberaceae, Zingiberis rhizoma recens] 10g, Ziziphus jujuba Mill. [Rhamnaceae, Jujubae fructus] 10g, Trichosanthes kirilowii Maxim. [Cucurbitaceae, Trichosanthis radix] 15g, Pseudostellaria heterophylla (Miq.) Pax [Caryophyllaceae, Pseudostellariae radix] 20g, Pheretima aspergillum (E.Perrier) [Megascolecidae, Pheretima] 10g, Spatholobus suberectus Dunn [Fabaceae, Spatholobi caulis] 30g, Salvia miltiorrhiza Bunge [Lamiaceae, Salviae miltiorrhizae radix et rhizoma] 20g, Buthus martensii Karsch [Buthidae, Scorpio] 5g, Glycyrrhiza glabra L. [Fabaceae, Glycyrrhizae radix et rhizoma] 5g |
| Guan 2021 | Tangmai Tongluo Decoction: Astragalus mongholicus Bunge [Fabaceae, Astragali radix] 30g, Carthamus tinctorius L. [Asteraceae, Carthami flos] 5g, Buthus martensii Karsch [Buthidae, Scorpio] 5g, Glycyrrhiza glabra L. [Fabaceae, Glycyrrhizae radix et rhizoma] 5g, Conioselinum anthriscoides 'Chuanxiong' [Apiaceae, Chuanxiong rhizoma] 10g, Angelica sinensis (Oliv.) Diels [Apiaceae, Angelicae sinensis radix] 10g, Pueraria montana var. lobata (Willd.) Maesen & S.M.Almeida ex Sanjappa & Predeep [Fabaceae, Puerariae lobatae radix] 10g, Achyranthes bidentata Blume [Amaranthaceae, Achyranthis bidentatae radix] 15g, Pheretima aspergillum (E.Perrier) [Megascolecidae, Pheretima] 15g, Poria cocos(Schw.)Wolf Poria [Polyporaceae, Poria] 15g, Trichosanthes kirilowii Maxim. [Cucurbitaceae, Trichosanthis radix] 15g, Salvia miltiorrhiza Bunge [Lamiaceae, Salviae miltiorrhizae radix et rhizoma] 20g, Pseudostellaria heterophylla (Miq.) Pax [Caryophyllaceae, Pseudostellariae radix] 20g |
| Liu 2022 | Yiqi Tongluo Qingre cream: Astragalus mongholicus Bunge [Fabaceae, Astragali radix] 40g, Codonopsis pilosula (Franch.) Nannf. [Campanulaceae, Codonopsis radix] 30g, Dendrobium nobile Lindl. [Orchidaceae, Dendrobii caulis] 15g, Pueraria montana var. lobata (Willd.) Maesen & S.M.Almeida ex Sanjappa & Predeep [Fabaceae, Puerariae lobatae radix] 20g, Rehmannia glutinosa (Gaertn.) DC. [Orobanchaceae, Rehmanniae Radix] 30g, Whitmania pigra Whitman [Hirudinidae, Hirudo] 15g, Pheretima aspergillum (E.Perrier) [Megascolecidae, Pheretima] 15g, Paeonia lactiflora Pall. [Paeoniaceae, Paeoniae radix rubra] 20g, Angelica sinensis (Oliv.) Diels [Apiaceae, Angelicae sinensis radix] 20g, Coptis chinensis Franch. [Ranunculaceae, Coptidis rhizoma] 5g, Scutellaria baicalensis Georgi [Lamiaceae, Scutellariae radix] 10g, Glycyrrhiza glabra L. [Fabaceae, Glycyrrhizae radix et rhizoma] 10g |
| Traditional Chinese patent medicines | |
| Wang 2013 (2) | Maixuekang Capsules: Whitmania pigra Whitman [Hirudinidae, Hirudo]; Yixinshu Capsules: Panax ginseng C.A.Mey. [Araliaceae, Ginseng radix et rhizoma] 200g, Ophiopogon japonicus (Thunb.) Ker Gawl. [Asparagaceae, Ophiopogonis radix] 200g, Schisandra chinensis (Turcz.) Baill. [Schisandraceae, Schisandrae chinensis fructus] 133g, Astragalus mongholicus Bunge [Fabaceae, Astragali radix] 200g, Salvia miltiorrhiza Bunge [Lamiaceae, Salviae miltiorrhizae radix et rhizoma] 267g, Conioselinum anthriscoides 'Chuanxiong' [Apiaceae, Chuanxiong rhizoma] 133g, Crataegus pinnatifida Bunge [Rosaceae, Crataegi fructus] 200g |
| Wang 2017 | Naoxintong Capsules: Astragalus mongholicus Bunge [Fabaceae, Astragali radix] 66g, Paeonia lactiflora Pall. [Paeoniaceae, Paeoniae radix rubra] 27g, Salvia miltiorrhiza Bunge [Lamiaceae, Salviae miltiorrhizae radix et rhizoma] 27g, Angelica sinensis (Oliv.) Diels [Apiaceae, Angelicae sinensis radix] 27g, Conioselinum anthriscoides 'Chuanxiong' [Apiaceae, Chuanxiong rhizoma] 27g, Prunus persica (L.) Batsch [Rosaceae, Persicae semen] 27g, Carthamus tinctorius L. [Asteraceae, Carthami flos] 13g, Boswellia frereana Birdw. [Burseraceae, Olibanum] 13g, Commiphora myrrha (T.Nees) Engl. [Burseraceae, Myrrha] 13g, Spatholobus suberectus Dunn [Fabaceae, Spatholobi caulis] 20g, Achyranthes bidentata Blume [Amaranthaceae, Achyranthis bidentatae radix] 27g, Neolitsea cassia (L.) Kosterm. [Lauraceae, Cinnamomi ramulus] 20g, Morus alba L. [Moraceae, Mori ramulus] 27g, Pheretima aspergillum (E.Perrier) [Megascolecidae, Pheretima] 27g, Buthus martensii Karsch [Buthidae, Scorpio] 13g, Whitmania pigra Whitman [Hirudinidae, Hirudo] 27g |
| Shou 2012 | Shexiang Baoxin Pills: Moschus berezovskii Flerov [Cervidae, Moschus], Panax ginseng C.A.Mey. [Araliaceae, Ginseng radix et rhizoma], Bos taurus domesticus Gmelin [Bovidae, Bovis calculus], Cinnamomum verum J.Presl [Lauraceae, Cinnamomi cortex], Liquidambar orientalis Mill. [Altingiaceae, Styrax], Bufo bufo gargarizans Cantor [Bufonidae, Bufonis venenum], Borneolum syntheticum |
| Yu 2018 | Yangxinshi Tablets: Astragalus mongholicus Bunge [Fabaceae, Astragali radix] 120g, Codonopsis pilosula (Franch.) Nannf. [Campanulaceae, Codonopsis radix] 100g, Salvia miltiorrhiza Bunge [Lamiaceae, Salviae miltiorrhizae radix et rhizoma] 80g, Pueraria montana var. lobata (Willd.) Maesen & S.M.Almeida ex Sanjappa & Predeep [Fabaceae, Puerariae lobatae radix] 80g, Epimedium sagittatum (Siebold & Zucc.) Maxim. [Berberidaceae, Epimedii folium] 80g, Crataegus pinnatifida Bunge [Rosaceae, Crataegi fructus] 80g, Rehmannia glutinosa (Gaertn.) DC. [Orobanchaceae, Rehmanniae Radix] 60g, Angelica sinensis (Oliv.) Diels [Apiaceae, Angelicae sinensis radix] 60g, Coptis chinensis Franch. [Ranunculaceae, Coptidis rhizoma] 60g, Corydalis yanhusuo (Y.H.Chou & Chun C.Hsu) W.T.Wang ex Z.Y.Su & C.Y.Wu [Papaveraceae, Corydalis rhizoma] 60g, Ganoderma lucidum（Leyss.ex Fr.）Karst. [Polyporaceae, Ganoderma] 60g, Panax ginseng C.A.Mey. [Araliaceae, Ginseng radix et rhizoma] 25g, Glycyrrhiza uralensis Fisch. ex DC. [Fabaceae, Glycyrrhizae radix et rhizoma praeparata cum melle] 25g |
| Traditional Chinese Medicine Extracts | |
| LI 2017 | Tanshinones Capsules: Salvia miltiorrhiza Bunge [Lamiaceae, Tanshinones] |
| Jiang 2021 | Xuesaitong Tablets: Panax notoginseng （Burk.） F.H.Chen [Araliaceae, Notoginseng total saponins] 50mg/tablet |
| Li 2016 (3) | Xinnao Shutong Tablet: Tribulus terrestris L. [Zygophyllaceae, Tribuli fructus] 15mg |
| Chen 2017 | Ginkgo biloba leaves: gGinkgo biloba L. [Ginkgoaceae, Ginkgo leaves extract] 40mg |
