# Supplementary material for: Mechanism of traditional Chinese medicine in elderly diabetes mellitus and a systematic review of its clinical application
Source: Front Pharmacol. 2024 Mar 6;15:1339148. doi: 10.3389/fphar.2024.1339148 (PMC10953506; doi:10.3389/fphar.2024.1339148)
Supplement: Supplementary file 2 [file DataSheet1.zip › Supplementary Table S1-17/Supplementary Table S16.docx]

Supplementary Table S16 | Adverse reactions and number of participants in the control group and intervention group.

| Adverse reactions in the control group | Symptom occurrence frequency | Number of participants experiencing symptoms | Adverse reactions in the interventional group | Symptom occurrence frequency | Number of participants experiencing symptoms |
| --- | --- | --- | --- | --- | --- |
| hypoglycemia | 13 | 42 | gastrointestinal discomfort | 9 | 24 |
| gastrointestinal discomfort | 7 | 19 | dizzy | 11 | 22 |
| dizzy | 13 | 18 | nausea | 12 | 18 |
| loss of appetite | 8 | 18 | hypoglycemia | 10 | 17 |
| diarrhea | 9 | 17 | diarrhea | 9 | 14 |
| vomit | 9 | 16 | vomit | 9 | 13 |
| nausea | 9 | 13 | loss of appetite | 5 | 10 |
| headache | 8 | 11 | rash | 5 | 9 |
| abdominal distension | 4 | 9 | abdominal distension | 5 | 8 |
| rash | 4 | 7 | headache | 5 | 6 |
| abnormal liver function | 4 | 6 | electrolyte disturbance | 1 | 3 |
| bleeding | 1 | 6 | hypotension | 2 | 3 |
| cardiovascular disease | 1 | 6 | abdominal pain | 2 | 2 |
| hypotension | 3 | 5 | blurred vision | 1 | 2 |
| feeble | 2 | 4 | cough | 1 | 2 |
| cough | 1 | 3 | drowsiness | 1 | 2 |
| dry | 3 | 3 | dry | 2 | 2 |
| abnormal renal function | 1 | 2 | fever | 1 | 2 |
| acute pancreatitis | 1 | 2 | acute pancreatitis | 1 | 1 |
| decreased WBC | 1 | 2 | cardiovascular disease | 1 | 1 |
| drowsiness | 1 | 2 | decreased PLT | 1 | 1 |
| electrolyte disturbance | 1 | 2 | feeble | 1 | 1 |
| proteinuria | 1 | 2 | flushed face | 1 | 1 |
| anemia | 1 | 1 | palpitate | 1 | 1 |
| diarrhea (1), dizzy | 1 | 1 | pharyngeal discomfort | 1 | 1 |
| fever | 1 | 1 | proteinuria | 1 | 1 |
| genital fungal infection | 1 | 1 |  |  |  |
| infect | 1 | 1 |  |  |  |
| palpitate | 1 | 1 |  |  |  |
| pharyngeal discomfort | 1 | 1 |  |  |  |
