# Supplementary material for: Mechanism of traditional Chinese medicine in elderly diabetes mellitus and a systematic review of its clinical application
Source: Front Pharmacol. 2024 Mar 6;15:1339148. doi: 10.3389/fphar.2024.1339148 (PMC10953506; doi:10.3389/fphar.2024.1339148)
Supplement: Supplementary file 2 [file DataSheet1.zip › Supplementary Table S1-17/Supplementary Table S17.docx]

Supplementary Table S17 | Jadad Scale.

| Study | The generation of random sequences | The generation of random sequences | The generation of random sequences | The generation of random sequences | The generation of random sequences |
| --- | --- | --- | --- | --- | --- |
| Cheng 2023 | 2 | 1 | 2 | 0 | 5 |
| Chen 2016 | 2 | 2 | 0 | 0 | 4 |
| Chen 2017 | 2 | 1 | 0 | 1 | 4 |
| Jiang 2019 | 2 | 1 | 0 | 1 | 4 |
| Xing 2023 | 2 | 1 | 0 | 1 | 4 |
| Lin 2019 | 2 | 1 | 0 | 1 | 4 |
| Liu 2022 | 2 | 1 | 0 | 1 | 4 |
| Luo 2014 | 2 | 1 | 0 | 1 | 4 |
| Ni 2021 | 2 | 1 | 0 | 1 | 4 |
| Sun 2012 (2) | 2 | 1 | 0 | 1 | 4 |
| WANG 2023 | 2 | 1 | 0 | 1 | 4 |
| Xu 2022 | 2 | 1 | 0 | 1 | 4 |
| Yu 2021 | 2 | 1 | 0 | 1 | 4 |
| Yu 2022 | 2 | 1 | 0 | 1 | 4 |
| Li 2023 | 2 | 1 | 0 | 0 | 3 |
| Ailiyasi 2019 | 2 | 1 | 0 | 0 | 3 |
| Zha 2022 | 2 | 1 | 0 | 0 | 3 |
| Chen 2022 | 2 | 1 | 0 | 0 | 3 |
| Chen 2021 | 2 | 1 | 0 | 0 | 3 |
| Cheng 2020 | 2 | 1 | 0 | 0 | 3 |
| Cheng 2019 | 2 | 1 | 0 | 0 | 3 |
| Deng 2015 | 2 | 1 | 0 | 0 | 3 |
| Feng 2023 | 2 | 1 | 0 | 0 | 3 |
| Gao 2010 | 2 | 1 | 0 | 0 | 3 |
| Guan 2021 | 2 | 1 | 0 | 0 | 3 |
| Guo 2016 | 2 | 1 | 0 | 0 | 3 |
| Guo 2020 | 2 | 1 | 0 | 0 | 3 |
| Han 2023 (2) | 2 | 1 | 0 | 0 | 3 |
| Hou 2019 | 2 | 1 | 0 | 0 | 3 |
| Hu 2014 | 2 | 1 | 0 | 0 | 3 |
| Hu 2016 | 2 | 1 | 0 | 0 | 3 |
| Hu 2018 (1) | 2 | 1 | 0 | 0 | 3 |
| Huang 2010 | 2 | 1 | 0 | 0 | 3 |
| Jiang 2021 | 2 | 1 | 0 | 0 | 3 |
| Jiang 2023 | 2 | 1 | 0 | 0 | 3 |
| Li 2020 | 2 | 1 | 0 | 0 | 3 |
| Li 2023 | 2 | 1 | 0 | 0 | 3 |
| Li 2016 (1) | 2 | 1 | 0 | 0 | 3 |
| Li 2016 (3) | 2 | 1 | 0 | 0 | 3 |
| Li 2019 | 2 | 1 | 0 | 0 | 3 |
| Liu 2023 | 2 | 1 | 0 | 0 | 3 |
| Liu 2018 | 2 | 1 | 0 | 0 | 3 |
| Liu 2008 | 1 | 1 | 0 | 1 | 3 |
| Ma 2022 | 2 | 1 | 0 | 0 | 3 |
| Ma 2017 (1) | 2 | 1 | 0 | 0 | 3 |
| Shen 2021 | 2 | 1 | 0 | 0 | 3 |
| Su 2019 | 2 | 1 | 0 | 0 | 3 |
| Su 2020 (2) | 2 | 1 | 0 | 0 | 3 |
| Su 2020 (1) | 2 | 1 | 0 | 0 | 3 |
| Sun 2022 | 1 | 1 | 1 | 0 | 3 |
| Wang 2018 (1) | 1 | 1 | 0 | 1 | 3 |
| Wang 2022 (2) | 2 | 1 | 0 | 0 | 3 |
| Wang 2020 | 2 | 1 | 0 | 0 | 3 |
| Wang 2017 | 2 | 1 | 0 | 0 | 3 |
| Wang 2013 (2) | 1 | 1 | 0 | 1 | 3 |
| Wang 2013 (1) | 1 | 1 | 0 | 1 | 3 |
| Wang 2012 (2) | 1 | 1 | 0 | 1 | 3 |
| Wei 2021 | 2 | 1 | 0 | 0 | 3 |
| Wu 2020 | 2 | 1 | 0 | 0 | 3 |
| Xia 2016 | 2 | 1 | 0 | 0 | 3 |
| Xu 2017 | 2 | 1 | 0 | 0 | 3 |
| XU 2020 | 2 | 1 | 0 | 0 | 3 |
| Xue 2010 | 1 | 1 | 0 | 1 | 3 |
| Yan 2019 | 1 | 1 | 0 | 1 | 3 |
| Yang 2019 | 2 | 1 | 0 | 0 | 3 |
| Yang 2021 (2) | 2 | 1 | 0 | 0 | 3 |
| Zhang 2003 (1) | 1 | 1 | 0 | 1 | 3 |
| Zhang 2003 (2) | 1 | 1 | 0 | 1 | 3 |
| Zhang 2021 | 2 | 1 | 0 | 0 | 3 |
| Zhang 2022 | 2 | 1 | 0 | 0 | 3 |
| Zhao 2022 | 2 | 1 | 0 | 0 | 3 |
| Zhao 2016 (2) | 2 | 1 | 0 | 0 | 3 |
| Zhong 2020 | 2 | 1 | 0 | 0 | 3 |
| Zhu 2013 | 1 | 1 | 0 | 1 | 3 |
| Zhu 2021 | 2 | 1 | 0 | 0 | 3 |
| BAI 2008 | 1 | 1 | 0 | 0 | 2 |
| Chen 2015 | 1 | 1 | 0 | 0 | 2 |
| Chen 2018 | 1 | 1 | 0 | 0 | 2 |
| Dai 2005 | 1 | 1 | 0 | 0 | 2 |
| DAI 2020 | 1 | 1 | 0 | 0 | 2 |
| Ding 2020 | 1 | 1 | 0 | 0 | 2 |
| Fang 2019 | 1 | 1 | 0 | 0 | 2 |
| Feng 2015 | 1 | 1 | 0 | 0 | 2 |
| Fu 2013 | 1 | 1 | 0 | 0 | 2 |
| Gao 2012 | 1 | 1 | 0 | 0 | 2 |
| Gao 2017 | 1 | 1 | 0 | 0 | 2 |
| Guo 2021 | 1 | 1 | 0 | 0 | 2 |
| Hong 2010 | 1 | 1 | 0 | 0 | 2 |
| Hu 2018 (2) | 1 | 1 | 0 | 0 | 2 |
| Hu 2017 | 1 | 1 | 0 | 0 | 2 |
| Huang 2014 | 1 | 1 | 0 | 0 | 2 |
| Jiang 2020 | 1 | 1 | 0 | 0 | 2 |
| Jin 2019 | 1 | 1 | 0 | 0 | 2 |
| Li 2022 (2) | 1 | 1 | 0 | 0 | 2 |
| Li 2018 (1) | 1 | 1 | 0 | 0 | 2 |
| LI 2017 | 1 | 1 | 0 | 0 | 2 |
| Li 2016 (2) | 1 | 1 | 0 | 0 | 2 |
| Li 2014 | 1 | 1 | 0 | 0 | 2 |
| Li 2015 | 1 | 1 | 0 | 0 | 2 |
| Li 2011 | 1 | 1 | 0 | 0 | 2 |
| Li 2022 (1) | 1 | 1 | 0 | 0 | 2 |
| Li 2012 | 1 | 1 | 0 | 0 | 2 |
| Li 2018 (2) | 1 | 1 | 0 | 0 | 2 |
| Lin 2022 | 1 | 1 | 0 | 0 | 2 |
| Lin 2021 | 1 | 1 | 0 | 0 | 2 |
| Liu 2016 | 1 | 1 | 0 | 0 | 2 |
| Liu 2011 | 1 | 1 | 0 | 0 | 2 |
| Liu 2018 | 1 | 1 | 0 | 0 | 2 |
| Liu 2016 | 1 | 1 | 0 | 0 | 2 |
| Ma 2017 (2) | 1 | 1 | 0 | 0 | 2 |
| Nie 2015 | 1 | 1 | 0 | 0 | 2 |
| Niu 2008 | 1 | 1 | 0 | 0 | 2 |
| Ou 2011 | 1 | 1 | 0 | 0 | 2 |
| Pan 2016 | 1 | 1 | 0 | 0 | 2 |
| Peng 2013 | 1 | 1 | 0 | 0 | 2 |
| Shen 2013 | 1 | 1 | 0 | 0 | 2 |
| Shi 2019 | 1 | 1 | 0 | 0 | 2 |
| Shou 2012 | 1 | 1 | 0 | 0 | 2 |
| Shu 2010 | 1 | 1 | 0 | 0 | 2 |
| Song 2016 | 1 | 1 | 0 | 0 | 2 |
| Sun 2012 (1) | 1 | 1 | 0 | 0 | 2 |
| Wang 2022 (3) | 1 | 1 | 0 | 0 | 2 |
| Wang 2022 (1) | 1 | 1 | 0 | 0 | 2 |
| Wang 2009 | 1 | 1 | 0 | 0 | 2 |
| Wang 2018 (2) | 1 | 1 | 0 | 0 | 2 |
| Wang 2007 | 1 | 1 | 0 | 0 | 2 |
| Wang 2012 (1) | 1 | 1 | 0 | 0 | 2 |
| Wang 2016 | 1 | 1 | 0 | 0 | 2 |
| Wei 2012 | 1 | 1 | 0 | 0 | 2 |
| Wen 2006 | 1 | 1 | 0 | 0 | 2 |
| Wu 2012 | 1 | 1 | 0 | 0 | 2 |
| Wu 2015 | 1 | 1 | 0 | 0 | 2 |
| Xiao 2021 | 1 | 1 | 0 | 0 | 2 |
| Xiao 2018 | 1 | 1 | 0 | 0 | 2 |
| Xiao 2020 | 1 | 1 | 0 | 0 | 2 |
| Xie 2016 | 1 | 1 | 0 | 0 | 2 |
| Yan 2014 | 1 | 1 | 0 | 0 | 2 |
| Yang 2021 (1) | 1 | 1 | 0 | 0 | 2 |
| Yang 2016 | 1 | 0 | 1 | 0 | 2 |
| Yi 2009 | 1 | 1 | 0 | 0 | 2 |
| Yu 2018 | 1 | 1 | 0 | 0 | 2 |
| Yu 2016 | 1 | 1 | 0 | 0 | 2 |
| Yu 2007 | 1 | 1 | 0 | 0 | 2 |
| Zhang 2014 | 1 | 1 | 0 | 0 | 2 |
| Zhang 2012 | 1 | 1 | 0 | 0 | 2 |
| Zhang 2019 | 1 | 1 | 0 | 0 | 2 |
| Zhao 2017 | 1 | 1 | 0 | 0 | 2 |
| Zhao 2016 (4) | 1 | 1 | 0 | 0 | 2 |
| Zhao 2016 (5) | 1 | 1 | 0 | 0 | 2 |
| Zhao 2016 (1) | 1 | 1 | 0 | 0 | 2 |
| Zhao 2016 (3) | 1 | 1 | 0 | 0 | 2 |
| Zhao 2021 | 1 | 1 | 0 | 0 | 2 |
| Zhong 2017 | 1 | 1 | 0 | 0 | 2 |
| Zhou 2013 | 1 | 1 | 0 | 0 | 2 |
| Zhou 2014 | 1 | 1 | 0 | 0 | 2 |
| Zhu 2014 | 1 | 1 | 0 | 0 | 2 |
| Zong 2017 | 1 | 1 | 0 | 0 | 2 |
| Zou 2017 | 1 | 1 | 0 | 0 | 2 |
| Bao 2019 | 1 | 0 | 0 | 0 | 1 |
| Han 2023 (1) | 1 | 0 | 0 | 0 | 1 |
