# Supplementary material for: Mechanism of traditional Chinese medicine in elderly diabetes mellitus and a systematic review of its clinical application
Source: Front Pharmacol. 2024 Mar 6;15:1339148. doi: 10.3389/fphar.2024.1339148 (PMC10953506; doi:10.3389/fphar.2024.1339148)
Supplement: Supplementary file 2 [file DataSheet1.zip › Supplementary Table S1-17/Supplementary Table S2.docx]

Supplementary Table S2 | Interventional drugs composition of TCM for elderly diabetes and islet function.

| Study | Interventional drugs composition |
| --- | --- |
| Traditional Chinese Prescription | |
| Zhou 2014 | Erban Decoction: Lobelia chinensis Lour. [Campanulaceae, Lobeliae chinensis herba] 15g, Scutellaria barbata D.Don [Lamiaceae, Scutellariae barbatae herba] 15g, Lonicera japonica Thunb. [Caprifoliaceae, Lonicerae japonicae flos] 15g, Astragalus mongholicus Bunge [Fabaceae, Astragali radix] 15g, Codonopsis pilosula (Franch.) Nannf. [Campanulaceae, Codonopsis radix] 15g, Dioscorea oppositifolia L. [Dioscoreaceae, Dioscoreae rhizoma] 15g, Coptis chinensis Franch. [Ranunculaceae, Coptidis rhizoma] 6g, Scrophularia ningpoensis Hemsl. [Scrophulariaceae, Scrophulariae radix] 10g, Cryptotympana pustulata Fabricius [Cicadidae, Cicadae periostracum] 10g, Atractylodes macrocephala Koidz. [Asteraceae, Atractylodis macrocephalae rhizoma] 10g, Aloe ferox Mill. [Asphodelaceae, Aloe] 10g, Glycyrrhiza glabra L. [Fabaceae, Glycyrrhizae radix et rhizoma] 10g, Ophiopogon japonicus (Thunb.) Ker Gawl. [Asparagaceae, Ophiopogonis radix] 12g, Bassia scoparia (L.) A.J.Scott [Amaranthaceae, Kochiae fructus] 12g |
| Sun 2022 | Gegen Qinlian Decoction: Rheum palmatum L. [Polygonaceae, Rhei radix et rhizoma] 6g, Coptis chinensis Franch. [Ranunculaceae, Coptidis rhizoma] 6g, Scutellaria baicalensis Georgi [Lamiaceae, Scutellariae radix] 6g, Anemarrhena asphodeloides Bunge [Asparagaceae, Anemarrhenae rhizoma] 9g, Pueraria montana var. lobata (Willd.) Maesen & S.M.Almeida ex Sanjappa & Predeep [Fabaceae, Puerariae lobatae radix] 9g |
| Jiang 2023 | Gegen Qinlian Decoction: Pueraria montana var. lobata (Willd.) Maesen & S.M.Almeida ex Sanjappa & Predeep [Fabaceae, Puerariae lobatae radix] 30g, Scutellaria baicalensis Georgi [Lamiaceae, Scutellariae radix] 15g, Coptis chinensis Franch. [Ranunculaceae, Coptidis rhizoma] 15g, Citrus reticulata Blanco [Rutaceae, Citri reticulatae pericarpium] 12g, Astragalus mongholicus Bunge [Fabaceae, Astragali radix] 10g, Glycyrrhiza glabra L. [Fabaceae, Glycyrrhizae radix et rhizoma] 6g, Zingiber officinale Roscoe [Zingiberaceae, Zingiberis rhizoma] 3g |
| Ni 2021 | Gegen Qinlian Decoction: Rheum palmatum L. [Polygonaceae, Rhei radix et rhizoma] 6g, Coptis chinensis Franch. [Ranunculaceae, Coptidis rhizoma] 6g, Scutellaria baicalensis Georgi [Lamiaceae, Scutellariae radix] 6g, Anemarrhena asphodeloides Bunge [Asparagaceae, Anemarrhenae rhizoma] 9g, Pueraria montana var. lobata (Willd.) Maesen & S.M.Almeida ex Sanjappa & Predeep [Fabaceae, Puerariae lobatae radix] 9g |
| Wang 2022 (1) | Huanglian Wendan Decoction: Coptis chinensis Franch. [Ranunculaceae, Coptidis rhizoma] 10g, Poria cocos (Schw.)Wolf Poria [Polyporaceae, Poria] 20g, Anemarrhena asphodeloides Bunge [Asparagaceae, Anemarrhenae rhizoma] 10g, Trichosanthes kirilowii Maxim. [Cucurbitaceae, Trichosanthis fructus] 20g, Bambusa tuldoides Munro [Poaceae, Bambusae caulis in taenias] 10g, Nelumbo nucifera Gaertn. [Nelumbonaceae, Nelumbinis folium] 20g, Alisma plantago-aquatica subsp. orientale (Sam.) Sam. [Alismataceae, Alismatis rhizoma] 15g, Zingiber officinale Roscoe [Zingiberaceae, Zingiberis rhizoma recens] 10g, Citrus reticulata Blanco [Rutaceae, Citri reticulatae pericarpium] 15g, Citrus × aurantium L. [Rutaceae, Aurantii fructus immaturus] 15g, Pinellia ternata (Thunb.) Makino [Araceae, Pinelliae rhizoma] 10g, Glycyrrhiza glabra L. [Fabaceae, Glycyrrhizae radix et rhizoma] 5g |
| Zhou 2013 | hypoglycemic basic formula: Astragalus mongholicus Bunge [Fabaceae, Astragali radix] 15g, Ophiopogon japonicus (Thunb.) Ker Gawl. [Asparagaceae, Ophiopogonis radix] 15g, Dioscorea oppositifolia L. [Dioscoreaceae, Dioscoreae rhizoma] 15g, Prunus mume (Siebold) Siebold & Zucc. [Rosaceae, Mume fructus] 12g, Poria cocos(Schw.)Wolf Poria [Polyporaceae, Poria] 10g, Pueraria montana var. lobata (Willd.) Maesen & S.M.Almeida ex Sanjappa & Predeep [Fabaceae, Puerariae lobatae radix] 30g, Salvia miltiorrhiza Bunge [Lamiaceae, Salviae miltiorrhizae radix et rhizoma] 18g |
| Liu 2016 | Invigorating the Spleen, Invigorating Qi and Tonifying the Kidney Decoction: Dioscorea oppositifolia L. [Dioscoreaceae, Dioscoreae rhizoma] 30g, Atractylodes lancea (Thunb.) DC. [Asteraceae, Atractylodis rhizoma] 15g, Pseudostellaria heterophylla (Miq.) Pax [Caryophyllaceae, Pseudostellariae radix] 15g, Cornus officinalis Siebold & Zucc. [Cornaceae, Corni fructus] 15g, Rehmannia glutinosa (Gaertn.) DC. [Orobanchaceae, Rehmanniae Radix] 30g, Salvia miltiorrhiza Bunge [Lamiaceae, Salviae miltiorrhizae radix et rhizoma] 30g, Coptis chinensis Franch. [Ranunculaceae, Coptidis rhizoma] 15g, Achyranthes bidentata Blume [Amaranthaceae, Achyranthis bidentatae radix] 15g, Astragalus mongholicus Bunge [Fabaceae, Astragali radix] 30g |
| Xue 2010 | Jiangtangjing Granules: Astragalus mongholicus Bunge [Fabaceae, Astragali radix], Rehmannia glutinosa (Gaertn.) DC. [Orobanchaceae, Rehmanniae Radix], Polygonatum sibiricum Redouté [Asparagaceae, Polygonati rhizoma], Dioscorea oppositifolia L. [Dioscoreaceae, Dioscoreae rhizoma], Crataegus pinnatifida Bunge [Rosaceae, Crataegi fructus], Pueraria montana var. lobata (Willd.) Maesen & S.M.Almeida ex Sanjappa & Predeep [Fabaceae, Puerariae lobatae radix], Coix lacryma-jobi var. ma-yuen (Rom.Caill.) Stapf [Poaceae, Coicis semen], Sinapis alba L. [Brassicaceae, Sinapis semen], Whitmania pigra Whitman [Hirudinidae, Hirudo] |
| Zhu 2013 | Jiangtangjing Granules: Astragalus mongholicus Bunge [Fabaceae, Astragali radix], Rehmannia glutinosa (Gaertn.) DC. [Orobanchaceae, Rehmanniae Radix], Polygonatum sibiricum Redouté [Asparagaceae, Polygonati rhizoma], Dioscorea oppositifolia L. [Dioscoreaceae, Dioscoreae rhizoma], Crataegus pinnatifida Bunge [Rosaceae, Crataegi fructus], Pueraria montana var. lobata (Willd.) Maesen & S.M.Almeida ex Sanjappa & Predeep [Fabaceae, Puerariae lobatae radix], Coix lacryma-jobi var. ma-yuen (Rom.Caill.) Stapf [Poaceae, Coicis semen], Sinapis alba L. [Brassicaceae, Sinapis semen], Whitmania pigra Whitman [Hirudinidae, Hirudo] |
| Fu 2013 | Jiawei Yuye Decoction: Astragalus mongholicus Bunge [Fabaceae, Astragali radix] 30g, Anemarrhena asphodeloides Bunge [Asparagaceae, Anemarrhenae rhizoma] 15g, Dioscorea oppositifolia L. [Dioscoreaceae, Dioscoreae rhizoma] 15g, Pueraria montana var. lobata (Willd.) Maesen & S.M.Almeida ex Sanjappa & Predeep [Fabaceae, Puerariae lobatae radix] 15g, Trichosanthes kirilowii Maxim. [Cucurbitaceae, Trichosanthis radix] 15g, Scrophularia ningpoensis Hemsl. [Scrophulariaceae, Scrophulariae radix] 12g, Atractylodes lancea (Thunb.) DC. [Asteraceae, Atractylodis rhizoma] 12g, Schisandra chinensis (Turcz.) Baill. [Schisandraceae, Schisandrae chinensis fructus] 12g, Gallus gallus domesticus Brisson [Phasianidae, Galli gigerii endothelium corneum] 10g, Pheretima aspergillum (E.Perrier) [Megascolecidae, Pheretima] 10g |
| Ding 2020 | Jingui Shenqi Prescription: Rehmannia glutinosa (Gaertn.) DC. [Orobanchaceae, Rehmanniae Radix] 30～60g, Cornus officinalis Siebold & Zucc. [Cornaceae, Corni fructus] 10g, Alisma plantago-aquatica subsp. orientale (Sam.) Sam. [Alismataceae, Alismatis rhizoma] 10g, Paeonia × suffruticosa Andrews [Paeoniaceae, Moutan cortex] 10g, Schisandra chinensis (Turcz.) Baill. [Schisandraceae, Schisandrae chinensis fructus] 10g, Asparagus cochinchinensis (Lour.) Merr. [Asparagaceae, Asparagi radix] 10g, Dioscorea oppositifolia L. [Dioscoreaceae, Dioscoreae rhizoma] 15～20g, Poria cocos(Schw.)Wolf Poria [Polyporaceae, Poria] 12g, Pueraria montana var. lobata (Willd.) Maesen & S.M.Almeida ex Sanjappa & Predeep [Fabaceae, Puerariae lobatae radix] 12g, Gypsum fibrosum 30g, Trichosanthes kirilowii Maxim. [Cucurbitaceae, Trichosanthis radix] 15g, Cinnamomum verum J.Presl [Lauraceae, Cinnamomi cortex] 2～3g |
| Wu 2015 | Jinlian Mixture: Euphorbia humifusa Willd. [Euphorbiaceae, Euphorbiae humifusae herba] 20g, Coptis chinensis Franch. [Ranunculaceae, Coptidis rhizoma] 6g, Astragalus mongholicus Bunge [Fabaceae, Astragali radix] 20g, Polygonatum sibiricum Redouté [Asparagaceae, Polygonati rhizoma] 15g |
| Ma 2022 | Liuwei Dihuang Decoction: Cornus officinalis Siebold & Zucc. [Cornaceae, Corni fructus] 10g, Rehmannia glutinosa (Gaertn.) DC. [Orobanchaceae, Rehmanniae radix praeparata] 20g, Alisma plantago-aquatica subsp. orientale (Sam.) Sam. [Alismataceae, Alismatis rhizoma] 12g, Atractylodes macrocephala Koidz. [Asteraceae, Atractylodis macrocephalae rhizoma] 12g, Poria cocos(Schw.)Wolf Poria [Polyporaceae, Poria] 20g, Paeonia × suffruticosa Andrews [Paeoniaceae, Moutan cortex] 10g, Astragalus mongholicus Bunge [Fabaceae, Astragali radix] 30g, Schisandra chinensis (Turcz.) Baill. [Schisandraceae, Schisandrae chinensis fructus] 8g, Pueraria montana var. lobata (Willd.) Maesen & S.M.Almeida ex Sanjappa & Predeep [Fabaceae, Puerariae lobatae radix] 12g, Epimedium sagittatum (Siebold & Zucc.) Maxim. [Berberidaceae, Epimedii folium] 12g, Ophiopogon japonicus (Thunb.) Ker Gawl. [Asparagaceae, Ophiopogonis radix] 12g, Anemarrhena asphodeloides Bunge [Asparagaceae, Anemarrhenae rhizoma] 12g |
| Zhu 2021 | Liuwei Dihuang Decoction: Rehmannia glutinosa (Gaertn.) DC. [Orobanchaceae, Rehmanniae Radix] 45g, Rehmannia glutinosa (Gaertn.) DC. [Orobanchaceae, Rehmanniae radix praeparata] 20g, Paeonia lactiflora Pall. [Paeoniaceae, Paeoniae radix alba] 20g, Astragalus mongholicus Bunge [Fabaceae, Astragali radix] 20g, Dioscorea oppositifolia L. [Dioscoreaceae, Dioscoreae rhizoma] 20g, Angelica sinensis (Oliv.) Diels [Apiaceae, Angelicae sinensis radix] 15g, Pseudostellaria heterophylla (Miq.) Pax [Caryophyllaceae, Pseudostellariae radix] 15g, Poria cocos(Schw.)Wolf Poria [Polyporaceae, Poria] 15g, Alisma plantago-aquatica subsp. orientale (Sam.) Sam. [Alismataceae, Alismatis rhizoma] 12g, Paeonia × suffruticosa Andrews [Paeoniaceae, Moutan cortex] 10g, Cornus officinalis Siebold & Zucc. [Cornaceae, Corni fructus] 10g, Trichosanthes kirilowii Maxim. [Cucurbitaceae, Trichosanthis radix] 10g, Prunus persica (L.) Batsch [Rosaceae, Persicae semen] 10g, Glycyrrhiza glabra L. [Fabaceae, Glycyrrhizae radix et rhizoma] 6g |
| Zhao 2022 | Liuwei Dihuang Pills: Rehmannia glutinosa (Gaertn.) DC. [Orobanchaceae, Rehmanniae radix praeparata] 15g, Dioscorea oppositifolia L. [Dioscoreaceae, Dioscoreae rhizoma] 15g, Paeonia × suffruticosa Andrews [Paeoniaceae, Moutan cortex] 15g, Alisma plantago-aquatica subsp. orientale (Sam.) Sam. [Alismataceae, Alismatis rhizoma] 15g, Cornus officinalis Siebold & Zucc. [Cornaceae, Corni fructus] 15g, Poria cocos(Schw.)Wolf Poria [Polyporaceae, Poria] 20g, Salvia miltiorrhiza Bunge [Lamiaceae, Salviae miltiorrhizae radix et rhizoma] 15g, Trichosanthes kirilowii Maxim. [Cucurbitaceae, Trichosanthis radix] 10g, Scutellaria baicalensis Georgi [Lamiaceae, Scutellariae radix] 10g, Rehmannia glutinosa (Gaertn.) DC. [Orobanchaceae, Rehmanniae Radix] 10g, Astragalus mongholicus Bunge [Fabaceae, Astragali radix] 20g, Asparagus cochinchinensis (Lour.) Merr. [Asparagaceae, Asparagi radix] 15g, Glycyrrhiza glabra L. [Fabaceae, Glycyrrhizae radix et rhizoma] 6g |
| Zha 2022 | Modified Shenqi Dihuang Decoction: Astragalus mongholicus Bunge [Fabaceae, Astragali radix] 30g, Pseudostellaria heterophylla (Miq.) Pax [Caryophyllaceae, Pseudostellariae radix] 30g, Rehmannia glutinosa (Gaertn.) DC. [Orobanchaceae, Rehmanniae radix praeparata] 15g, Poria cocos (Schw.) Wolf Poria [Polyporaceae, Poria] 15g, Paeonia × suffruticosa Andrews [Paeoniaceae, Moutan cortex] 15g, Cornus officinalis Siebold & Zucc. [Cornaceae, Corni fructus] 15g, Alisma plantago-aquatica subsp. orientale (Sam.) Sam. [Alismataceae, Alismatis rhizoma] 10g, Dioscorea oppositifolia L. [Dioscoreaceae, Dioscoreae rhizoma] 10g, Panax notoginseng (Burkill) F.H.Chen [Araliaceae, Notoginseng radix et rhizoma] 3g |
| Zou 2017 | Modified Taohe Chengqi Decoction: Prunus persica (L.) Batsch [Rosaceae, Persicae semen], Rheum palmatum L. [Polygonaceae, Rhei radix et rhizoma], Neolitsea cassia (L.) Kosterm. [Lauraceae, Cinnamomi ramulus], Ophiopogon japonicus (Thunb.) Ker Gawl. [Asparagaceae, Ophiopogonis radix], Natrii sulfas exsiccatus, Glycyrrhiza glabra L. [Fabaceae, Glycyrrhizae radix et rhizoma], Rehmannia glutinosa (Gaertn.) DC. [Orobanchaceae, Rehmanniae Radix] |
| Han 2023 （1） | Renshen Baihu Decoction: Gypsum fibrosum 30g, Panax ginseng C.A.Mey. [Araliaceae, Ginseng radix et rhizoma] 5g, Anemarrhena asphodeloides Bunge [Asparagaceae, Anemarrhenae rhizoma] 10g, Paeonia lactiflora Pall. [Paeoniaceae, Paeoniae radix alba] 15g, Dioscorea oppositifolia L. [Dioscoreaceae, Dioscoreae rhizoma] 15g, Polygonatum sibiricum Redouté [Asparagaceae, Polygonati rhizoma] 15g, Scrophularia ningpoensis Hemsl. [Scrophulariaceae, Scrophulariae radix] 15g, Rehmannia glutinosa (Gaertn.) DC. [Orobanchaceae, Rehmanniae Radix] 15g, Lycium barbarum L. [Solanaceae, Lycii cortex] 9g, Trichosanthes kirilowii Maxim. [Cucurbitaceae, Trichosanthis radix] 9g, Ophiopogon japonicus (Thunb.) Ker Gawl. [Asparagaceae, Ophiopogonis radix] 9g, Coptis chinensis Franch. [Ranunculaceae, Coptidis rhizoma] 9g |
| Wei 2021 | Sanhuang Decoction: Rheum palmatum L. [Polygonaceae, Rhei radix et rhizoma] 6g, Coptis chinensis Franch. [Ranunculaceae, Coptidis rhizoma] 3g, Scutellaria baicalensis Georgi [Lamiaceae, Scutellariae radix] 10g |
| Ailiyasi 2019 | Sanhuang Decoction: Rheum palmatum L. [Polygonaceae, Rhei radix et rhizoma] 6g, Coptis chinensis Franch. [Ranunculaceae, Coptidis rhizoma] 3g, Scutellaria baicalensis Georgi [Lamiaceae, Scutellariae radix] 10g |
| XU 2020 | self-designed Yangyin Xiaoke Recipe: Ophiopogon japonicus (Thunb.) Ker Gawl. [Asparagaceae, Ophiopogonis radix] 10g, Ligustrum lucidum W.T.Aiton [Oleaceae, Ligustri lucidi fructus] 10g, Rehmannia glutinosa (Gaertn.) DC. [Orobanchaceae, Rehmanniae radix praeparata] 15g, Glycyrrhiza glabra L. [Fabaceae, Glycyrrhizae radix et rhizoma] 10g, Dendrobium nobile Lindl. [Orchidaceae, Dendrobii caulis] 10g, Astragalus mongholicus Bunge [Fabaceae, Astragali radix] 15g, Codonopsis pilosula (Franch.) Nannf. [Campanulaceae, Codonopsis radix] 12g, Atractylodes macrocephala Koidz. [Asteraceae, Atractylodis macrocephalae rhizoma] 20g, Dioscorea oppositifolia L. [Dioscoreaceae, Dioscoreae rhizoma] 15g |
| Wang 2013 (1) | Shendi Shengjin Capsule: Panax ginseng C.A.Mey. [Araliaceae, Ginseng radix et rhizoma], Rehmannia glutinosa (Gaertn.) DC. [Orobanchaceae, Rehmanniae Radix], Ophiopogon japonicus (Thunb.) Ker Gawl. [Asparagaceae, Ophiopogonis radix], Salvia miltiorrhiza Bunge [Lamiaceae, Salviae miltiorrhizae radix et rhizoma], Panax notoginseng (Burkill) F.H.Chen [Araliaceae, Notoginseng radix et rhizoma], Astragalus mongholicus Bunge [Fabaceae, Astragali radix], Pueraria montana var. lobata (Willd.) Maesen & S.M.Almeida ex Sanjappa & Predeep [Fabaceae, Puerariae lobatae radix], Dioscorea oppositifolia L. [Dioscoreaceae, Dioscoreae rhizoma] |
| Li 2014 | Shengmai San and Liuwei Dihuang Pills: Codonopsis pilosula (Franch.) Nannf. [Campanulaceae, Codonopsis radix] 15g, Schisandra chinensis (Turcz.) Baill. [Schisandraceae, Schisandrae chinensis fructus] 6g, Ophiopogon japonicus (Thunb.) Ker Gawl. [Asparagaceae, Ophiopogonis radix] 15g, Cornus officinalis Siebold & Zucc. [Cornaceae, Corni fructus] 10g, Dioscorea oppositifolia L. [Dioscoreaceae, Dioscoreae rhizoma] 15g, Paeonia × suffruticosa Andrews [Paeoniaceae, Moutan cortex] 10g, Poria cocos(Schw.)Wolf Poria [Polyporaceae, Poria] 15g, Alisma plantago-aquatica subsp. orientale (Sam.) Sam. [Alismataceae, Alismatis rhizoma] 10g, Coptis chinensis Franch. [Ranunculaceae, Coptidis rhizoma] 6g, Rehmannia glutinosa (Gaertn.) DC. [Orobanchaceae, Rehmanniae Radix] 15g, Astragalus mongholicus Bunge [Fabaceae, Astragali radix] 20g |
| Ma 2017 (1) | Shenqi Maiwei Dihuang decoction: Astragalus mongholicus Bunge [Fabaceae, Astragali radix] 20g, Pseudostellaria heterophylla (Miq.) Pax [Caryophyllaceae, Pseudostellariae radix] 30g, Cornus officinalis Siebold & Zucc. [Cornaceae, Corni fructus] 15g, Rehmannia glutinosa (Gaertn.) DC. [Orobanchaceae, Rehmanniae radix praeparata] 15g, Poria cocos (Schw.) Wolf Poria [Polyporaceae, Poria] 15g, Paeonia × suffruticosa Andrews [Paeoniaceae, Moutan cortex] 10g, Alisma plantago-aquatica subsp. orientale (Sam.) Sam. [Alismataceae, Alismatis rhizoma] 10g, Panax notoginseng (Burkill) F.H.Chen [Araliaceae, Notoginseng radix et rhizoma] 3g |
| Su 2020 (1) | Yangyin Xiaoke Decoction: Citrus × aurantium L. [Rutaceae, Aurantii fructus] 6g, Glycyrrhiza glabra L. [Fabaceae, Glycyrrhizae radix et rhizoma] 6g, Lycium barbarum L. [Solanaceae, Lycii fructus] 12g, Anemarrhena asphodeloides Bunge [Asparagaceae, Anemarrhenae rhizoma] 12g, Polygonatum sibiricum Redouté [Asparagaceae, Polygonati rhizoma] 12g, Trichosanthes kirilowii Maxim. [Cucurbitaceae, Trichosanthis radix] 20g, Astragalus mongholicus Bunge [Fabaceae, Astragali radix] 30g, Pueraria montana var. lobata (Willd.) Maesen & S.M.Almeida ex Sanjappa & Predeep [Fabaceae, Puerariae lobatae radix] 15g, Dioscorea oppositifolia L. [Dioscoreaceae, Dioscoreae rhizoma] 15g, Ophiopogon japonicus (Thunb.) Ker Gawl. [Asparagaceae, Ophiopogonis radix] 15g, Coptis chinensis Franch. [Ranunculaceae, Coptidis rhizoma] 15g, Rehmannia glutinosa (Gaertn.) DC. [Orobanchaceae, Rehmanniae Radix] 15g, Salvia miltiorrhiza Bunge [Lamiaceae, Salviae miltiorrhizae radix et rhizoma] 15g |
| Li 2011 | Yiqi Bushen Huoxue Recipe: Astragalus mongholicus Bunge [Fabaceae, Astragali radix] 40g, Polygonatum sibiricum Redouté [Asparagaceae, Polygonati rhizoma] 30g, Codonopsis pilosula (Franch.) Nannf. [Campanulaceae, Codonopsis radix] 15g, Atractylodes macrocephala Koidz. [Asteraceae, Atractylodis macrocephalae rhizoma] 15g, Poria cocos (Schw.) Wolf Poria [Polyporaceae, Poria] 20g, Dioscorea oppositifolia L. [Dioscoreaceae, Dioscoreae rhizoma] 20g, Eucommia ulmoides Oliv. [Eucommiaceae, Eucommiae cortex] 15g, Rehmannia glutinosa (Gaertn.) DC. [Orobanchaceae, Rehmanniae Radix] 20g, Cornus officinalis Siebold & Zucc. [Cornaceae, Corni fructus] 15g, Polygonatum odoratum (Mill.) Druce [Asparagaceae, Polygonati odorati rhizoma] 15g, Ophiopogon japonicus (Thunb.) Ker Gawl. [Asparagaceae, Ophiopogonis radix] 15g, Lycium barbarum L. [Solanaceae, Lycii fructus] 15g, Salvia miltiorrhiza Bunge [Lamiaceae, Salviae miltiorrhizae radix et rhizoma] 15g, Paeonia lactiflora Pall. [Paeoniaceae, Paeoniae radix rubra] 20g, Conioselinum anthriscoides 'Chuanxiong' [Apiaceae, Chuanxiong rhizoma] 15g, Liquidambar formosana Hance [Altingiaceae, Liquidambaris fructus] 20g |
| DAI 2020 | Yiqi Yangyin Bushen Recipe: Gallus gallus domesticus Brisson [Phasianidae, Galli gigerii endothelium corneum] 9g, Glycyrrhiza glabra L. [Fabaceae, Glycyrrhizae radix et rhizoma] 6g, Pueraria montana var. lobata (Willd.) Maesen & S.M.Almeida ex Sanjappa & Predeep [Fabaceae, Puerariae lobatae radix] 15g, Salvia miltiorrhiza Bunge [Lamiaceae, Salviae miltiorrhizae radix et rhizoma] 9g, Ligustrum lucidum W.T.Aiton [Oleaceae, Ligustri lucidi fructus] 12g, Cullen corylifolium (L.) Medik. [Fabaceae, Psoraleae fructus] 9g, Lycium barbarum L. [Solanaceae, Lycii fructus] 15g, Cornus officinalis Siebold & Zucc. [Cornaceae, Corni fructus] 9g, Dioscorea oppositifolia L. [Dioscoreaceae, Dioscoreae rhizoma] 30g, Rehmannia glutinosa (Gaertn.) DC. [Orobanchaceae, Rehmanniae radix praeparata] 15g, Astragalus mongholicus Bunge [Fabaceae, Astragali radix] 30g |
| Zhao 2016 (1) | Yuye Decoction: Dioscorea oppositifolia L. [Dioscoreaceae, Dioscoreae rhizoma] 30g, Astragalus mongholicus Bunge [Fabaceae, Astragali radix] 15g, Anemarrhena asphodeloides Bunge [Asparagaceae, Anemarrhenae rhizoma] 12g, Gallus gallus domesticus Brisson [Phasianidae, Galli gigerii endothelium corneum] 6g, Pueraria montana var. lobata (Willd.) Maesen & S.M.Almeida ex Sanjappa & Predeep [Fabaceae, Puerariae lobatae radix] 15g, Schisandra chinensis (Turcz.) Baill. [Schisandraceae, Schisandrae chinensis fructus] 10g, Trichosanthes kirilowii Maxim. [Cucurbitaceae, Trichosanthis radix] 10g |
| Traditional Chinese patent medicines | |
| Wu 2012 | Shenqi Jiangtang Granules: Panax ginseng C.A.Mey. [Araliaceae, Total ginsenoside of ginseng stems and leaves], Astragalus mongholicus Bunge [Fabaceae, Astragali radix], Rehmannia glutinosa (Gaertn.) DC. [Orobanchaceae, Rehmanniae Radix], Dioscorea oppositifolia L. [Dioscoreaceae, Dioscoreae rhizoma], Trichosanthes kirilowii Maxim. [Cucurbitaceae, Trichosanthis radix], Rubus chingii Hu [Rosaceae, Rubi fructus], Ophiopogon japonicus (Thunb.) Ker Gawl. [Asparagaceae, Ophiopogonis radix], Schisandra chinensis (Turcz.) Baill. [Schisandraceae, Schisandrae chinensis fructus], Lycium barbarum L. [Solanaceae, Lycii fructus], Alisma plantago-aquatica subsp. orientale (Sam.) Sam. [Alismataceae, Alismatis rhizoma], Poria cocos (Schw.) Wolf Poria [Polyporaceae, Poria] |
| Liu 2018 | Shenqi Jiangtang Granules: Panax ginseng C.A.Mey. [Araliaceae, Total ginsenoside of ginseng stems and leaves], Astragalus mongholicus Bunge [Fabaceae, Astragali radix], Rehmannia glutinosa (Gaertn.) DC. [Orobanchaceae, Rehmanniae Radix], Dioscorea oppositifolia L. [Dioscoreaceae, Dioscoreae rhizoma], Trichosanthes kirilowii Maxim. [Cucurbitaceae, Trichosanthis radix], Rubus chingii Hu [Rosaceae, Rubi fructus], Ophiopogon japonicus (Thunb.) Ker Gawl. [Asparagaceae, Ophiopogonis radix], Schisandra chinensis (Turcz.) Baill. [Schisandraceae, Schisandrae chinensis fructus], Lycium barbarum L. [Solanaceae, Lycii fructus], Alisma plantago-aquatica subsp. orientale (Sam.) Sam. [Alismataceae, Alismatis rhizoma], Poria cocos (Schw.)Wolf Poria [Polyporaceae, Poria] |
| Xia 2016 | Shenqi Jiangtang Granules: Panax ginseng C.A.Mey. [Araliaceae, Total ginsenoside of ginseng stems and leaves], Astragalus mongholicus Bunge [Fabaceae, Astragali radix], Rehmannia glutinosa (Gaertn.) DC. [Orobanchaceae, Rehmanniae Radix], Dioscorea oppositifolia L. [Dioscoreaceae, Dioscoreae rhizoma], Trichosanthes kirilowii Maxim. [Cucurbitaceae, Trichosanthis radix], Rubus chingii Hu [Rosaceae, Rubi fructus], Ophiopogon japonicus (Thunb.) Ker Gawl. [Asparagaceae, Ophiopogonis radix], Schisandra chinensis (Turcz.) Baill. [Schisandraceae, Schisandrae chinensis fructus], Lycium barbarum L. [Solanaceae, Lycii fructus], Alisma plantago-aquatica subsp. orientale (Sam.) Sam. [Alismataceae, Alismatis rhizoma], Poria cocos (Schw.)Wolf Poria [Polyporaceae, Poria] |
| Bao 2019 | Danzhi Jiangtang Capsules: Pseudostellaria heterophylla (Miq.) Pax [Caryophyllaceae, Pseudostellariae radix], Paeonia × suffruticosa Andrews [Paeoniaceae, Moutan cortex], Whitmania pigra Whitman [Hirudinidae, Hirudo], Rehmannia glutinosa (Gaertn.) DC. [Orobanchaceae, Rehmanniae Radix], Cuscuta chinensis Lam. [Convolvulaceae, Cuscutae semen], Alisma plantago-aquatica subsp. orientale (Sam.) Sam. [Alismataceae, Alismatis rhizoma] |
| Niu 2008 | Danzhi Jiangtang Capsules: Pseudostellaria heterophylla (Miq.) Pax [Caryophyllaceae, Pseudostellariae radix], Paeonia × suffruticosa Andrews [Paeoniaceae, Moutan cortex], Whitmania pigra Whitman [Hirudinidae, Hirudo], Rehmannia glutinosa (Gaertn.) DC. [Orobanchaceae, Rehmanniae Radix], Cuscuta chinensis Lam. [Convolvulaceae, Cuscutae semen], Alisma plantago-aquatica subsp. orientale (Sam.) Sam. [Alismataceae, Alismatis rhizoma] |
| Deng 2015 | Jinkui Shenqi Pills: Poria cocos (Schw.) Wolf Poria [Polyporaceae, Poria], Rehmannia glutinosa (Gaertn.) DC. [Orobanchaceae, Rehmanniae Radix], Aconitum carmichaelii Debeaux [Ranunculaceae, Aconiti lateralis radix praeparata], Alisma plantago-aquatica subsp. orientale (Sam.) Sam. [Alismataceae, Alismatis rhizoma], Achyranthes bidentata Blume [Amaranthaceae, Achyranthis bidentatae radix], Paeonia × suffruticosa Andrews [Paeoniaceae, Moutan cortex], Cornus officinalis Siebold & Zucc. [Cornaceae, Corni fructus], Plantago asiatica L. [Plantaginaceae, Plantaginis semen] |
| Cheng 2020 | Shiwei Yuquan Tablets: Panax ginseng C.A.Mey. [Araliaceae, Ginseng radix et rhizoma], Ophiopogon japonicus (Thunb.) Ker Gawl. [Asparagaceae, Ophiopogonis radix], Schisandra chinensis (Turcz.) Baill. [Schisandraceae, Schisandrae chinensis fructus], Trichosanthes kirilowii Maxim. [Cucurbitaceae, Trichosanthis radix], Rehmannia glutinosa (Gaertn.) DC. [Orobanchaceae, Rehmanniae Radix], Astragalus mongholicus Bunge [Fabaceae, Astragali radix], Prunus mume (Siebold) Siebold & Zucc. [Rosaceae, Mume fructus], Pueraria montana var. lobata (Willd.) Maesen & S.M.Almeida ex Sanjappa & Predeep [Fabaceae, Puerariae lobatae radix], Poria cocos(Schw.)Wolf Poria [Polyporaceae, Poria], Glycyrrhiza glabra L. [Fabaceae, Glycyrrhizae radix et rhizoma] |
| Xiao 2021 | Yuquan Pills: Pueraria montana var. lobata (Willd.) Maesen & S.M.Almeida ex Sanjappa & Predeep [Fabaceae, Puerariae lobatae radix], Trichosanthes kirilowii Maxim. [Cucurbitaceae, Trichosanthis radix], Rehmannia glutinosa (Gaertn.) DC. [Orobanchaceae, Rehmanniae Radix], Ophiopogon japonicus (Thunb.) Ker Gawl. [Asparagaceae, Ophiopogonis radix], Schisandra chinensis (Turcz.) Baill. [Schisandraceae, Schisandrae chinensis fructus], Glycyrrhiza glabra L. [Fabaceae, Glycyrrhizae radix et rhizoma] |
| Li 2023 | Liuwei Dihuang Pills: Rehmannia glutinosa (Gaertn.) DC. [Orobanchaceae, Rehmanniae radix praeparata] 160g, Cornus officinalis Siebold & Zucc. [Cornaceae, Corni fructus] 80g, Paeonia × suffruticosa Andrews [Paeoniaceae, Moutan cortex] 60g, Dioscorea oppositifolia L. [Dioscoreaceae, Dioscoreae rhizoma] 80g, Smilax glabra Roxb. [Smilacaceae, Smilacis glabrae rhizoma] 60g, Alisma plantago-aquatica subsp. orientale (Sam.) Sam. [Alismataceae, Alismatis rhizoma] 60g |
| Xiao 2018 | Xiaoke Pills: Pueraria montana var. lobata (Willd.) Maesen & S.M.Almeida ex Sanjappa & Predeep [Fabaceae, Puerariae lobatae radix] 265g, Rehmannia glutinosa (Gaertn.) DC. [Orobanchaceae, Rehmanniae Radix] 159g, Astragalus mongholicus Bunge [Fabaceae, Astragali radix] 53g, Trichosanthes kirilowii Maxim. [Cucurbitaceae, Trichosanthis radix] 265g, Zea mays L. [Poaceae, corn silk] 265g, Schisandra chinensis (Turcz.) Baill. [Schisandraceae, Schisandrae chinensis fructus] 53g, Dioscorea oppositifolia L. [Dioscoreaceae, Dioscoreae rhizoma] 26.5g, Glibenclamide 0.25g |
| Li 2022 (1) | Shenqi Jiangtang Tablets: Panax ginseng C.A.Mey. [Araliaceae, Total ginsenoside of ginseng stems and leaves] 6g, Astragalus mongholicus Bunge [Fabaceae, Astragali radix] 124g, Rehmannia glutinosa (Gaertn.) DC. [Orobanchaceae, Rehmanniae Radix] 186g, Dioscorea oppositifolia L. [Dioscoreaceae, Dioscoreae rhizoma] 62g, Trichosanthes kirilowii Maxim. [Cucurbitaceae, Trichosanthis radix] 62g, Rubus chingii Hu [Rosaceae, Rubi fructus] 31g, Ophiopogon japonicus (Thunb.) Ker Gawl. [Asparagaceae, Ophiopogonis radix] 62g, Schisandra chinensis (Turcz.) Baill. [Schisandraceae, Schisandrae chinensis fructus] 62g, Lycium barbarum L. [Solanaceae, Lycii fructus] 124g, Alisma plantago-aquatica subsp. orientale (Sam.) Sam. [Alismataceae, Alismatis rhizoma] 62g, Poria cocos (Schw.) Wolf Poria [Polyporaceae, Poria] 62g |
| Yu 2007 | Jinqi Jiangtang Tablets: Coptis chinensis Franch. [Ranunculaceae, Coptidis rhizoma] 343g, Astragalus mongholicus Bunge [Fabaceae, Astragali radix] 513g, Lonicera japonica Thunb. [Caprifoliaceae, Lonicerae japonicae flos] 2058g |
| Han 2023 （2） | Jinlida Granules: Panax ginseng C.A.Mey. [Araliaceae, Ginseng radix et rhizoma] 184.5g, Polygonatum sibiricum Redouté [Asparagaceae, Polygonati rhizoma] 244.5g, Atractylodes lancea (Thunb.) DC. [Asteraceae, Atractylodis rhizoma] 122.2g, Sophora flavescens Aiton [Fabaceae, Sophorae flavescentis radix] 100g, Ophiopogon japonicus (Thunb.) Ker Gawl. [Asparagaceae, Ophiopogonis radix] 244.5g, Rehmannia glutinosa (Gaertn.) DC. [Orobanchaceae, Rehmanniae Radix] 184.5g, Reynoutria multiflora (Thunb.) Moldenke [Polygonaceae, Polygoni multiflori radix] 149g, Cornus officinalis Siebold & Zucc. [Cornaceae, Corni fructus] 244.5g, Poria cocos (Schw.) Wolf Poria [Polyporaceae, Poria] 149g, Eupatorium fortunei Turcz. [Asteraceae, Eupatorii herba] 100g, Coptis chinensis Franch. [Ranunculaceae, Coptidis rhizoma] 100g, Anemarrhena asphodeloides Bunge [Asparagaceae, Anemarrhenae rhizoma] 122.2g, Epimedium sagittatum (Siebold & Zucc.) Maxim. [Berberidaceae, Epimedii folium] 100g, Salvia miltiorrhiza Bunge [Lamiaceae, Salviae miltiorrhizae radix et rhizoma] 160g, Pueraria montana var. lobata (Willd.) Maesen & S.M.Almeida ex Sanjappa & Predeep [Fabaceae, Puerariae lobatae radix] 244.5g, Litchi chinensis Sonn. [Sapindaceae, Litchi semen] 244.5g, Lycium barbarum L. [Solanaceae, Lycii cortex] 149g |
| Jiang 2020 | Jinlida Granules: Panax ginseng C.A.Mey. [Araliaceae, Ginseng radix et rhizoma] 184.5g, Polygonatum sibiricum Redouté [Asparagaceae, Polygonati rhizoma] 244.5g, Atractylodes lancea (Thunb.) DC. [Asteraceae, Atractylodis rhizoma] 122.2g, Sophora flavescens Aiton [Fabaceae, Sophorae flavescentis radix] 100g, Ophiopogon japonicus (Thunb.) Ker Gawl. [Asparagaceae, Ophiopogonis radix] 244.5g, Rehmannia glutinosa (Gaertn.) DC. [Orobanchaceae, Rehmanniae Radix] 184.5g, Reynoutria multiflora (Thunb.) Moldenke [Polygonaceae, Polygoni multiflori radix] 149g, Cornus officinalis Siebold & Zucc. [Cornaceae, Corni fructus] 244.5g, Poria cocos (Schw.) Wolf Poria [Polyporaceae, Poria] 149g, Eupatorium fortunei Turcz. [Asteraceae, Eupatorii herba] 100g, Coptis chinensis Franch. [Ranunculaceae, Coptidis rhizoma] 100g, Anemarrhena asphodeloides Bunge [Asparagaceae, Anemarrhenae rhizoma] 122.2g, Epimedium sagittatum (Siebold & Zucc.) Maxim. [Berberidaceae, Epimedii folium] 100g, Salvia miltiorrhiza Bunge [Lamiaceae, Salviae miltiorrhizae radix et rhizoma] 160g, Pueraria montana var. lobata (Willd.) Maesen & S.M.Almeida ex Sanjappa & Predeep [Fabaceae, Puerariae lobatae radix] 244.5g, Litchi chinensis Sonn. [Sapindaceae, Litchi semen] 244.5g, Lycium barbarum L. [Solanaceae, Lycii cortex] 149g |
| Hu 2014 | Jinlida Granules: Panax ginseng C.A.Mey. [Araliaceae, Ginseng radix et rhizoma] 184.5g, Polygonatum sibiricum Redouté [Asparagaceae, Polygonati rhizoma] 244.5g, Atractylodes lancea (Thunb.) DC. [Asteraceae, Atractylodis rhizoma] 122.2g, Sophora flavescens Aiton [Fabaceae, Sophorae flavescentis radix] 100g, Ophiopogon japonicus (Thunb.) Ker Gawl. [Asparagaceae, Ophiopogonis radix] 244.5g, Rehmannia glutinosa (Gaertn.) DC. [Orobanchaceae, Rehmanniae Radix] 184.5g, Reynoutria multiflora (Thunb.) Moldenke [Polygonaceae, Polygoni multiflori radix] 149g, Cornus officinalis Siebold & Zucc. [Cornaceae, Corni fructus] 244.5g, Poria cocos (Schw.) Wolf Poria [Polyporaceae, Poria] 149g, Eupatorium fortunei Turcz. [Asteraceae, Eupatorii herba] 100g, Coptis chinensis Franch. [Ranunculaceae, Coptidis rhizoma] 100g, Anemarrhena asphodeloides Bunge [Asparagaceae, Anemarrhenae rhizoma] 122.2g, Epimedium sagittatum (Siebold & Zucc.) Maxim. [Berberidaceae, Epimedii folium] 100g, Salvia miltiorrhiza Bunge [Lamiaceae, Salviae miltiorrhizae radix et rhizoma] 160g, Pueraria montana var. lobata (Willd.) Maesen & S.M.Almeida ex Sanjappa & Predeep [Fabaceae, Puerariae lobatae radix] 244.5g, Litchi chinensis Sonn. [Sapindaceae, Litchi semen] 244.5g, Lycium barbarum L. [Solanaceae, Lycii cortex] 149g |
| Wu 2020 | Liuwei Dihuang Pills: Rehmannia glutinosa (Gaertn.) DC. [Orobanchaceae, Rehmanniae radix praeparata] 160g, Cornus officinalis Siebold & Zucc. [Cornaceae, Corni fructus] 80g, Paeonia × suffruticosa Andrews [Paeoniaceae, Moutan cortex] 60g, Dioscorea oppositifolia L. [Dioscoreaceae, Dioscoreae rhizoma] 80g, Smilax glabra Roxb. [Smilacaceae, Smilacis glabrae rhizoma] 60g, Alisma plantago-aquatica subsp. orientale (Sam.) Sam. [Alismataceae, Alismatis rhizoma] 60g |
| Hou 2019 | Maiwei Dihuang Pills: Ophiopogon japonicus (Thunb.) Ker Gawl. [Asparagaceae, Ophiopogonis radix] 60g, Schisandra chinensis (Turcz.) Baill. [Schisandraceae, Schisandrae chinensis fructus] 40g, Rehmannia glutinosa (Gaertn.) DC. [Orobanchaceae, Rehmanniae radix praeparata] 160g, Cornus officinalis Siebold & Zucc. [Cornaceae, Corni fructus] 80g, Paeonia × suffruticosa Andrews [Paeoniaceae, Moutan cortex] 60g, Dioscorea oppositifolia L. [Dioscoreaceae, Dioscoreae rhizoma] 80g, Poria cocos (Schw.) Wolf Poria [Polyporaceae, Poria] 60g, Alisma plantago-aquatica subsp. orientale (Sam.) Sam. [Alismataceae, Alismatis rhizoma] 60g |
| Zhang 2003 (1) | Jinqi Jiangtang Tablets: Coptis chinensis Franch. [Ranunculaceae, Coptidis rhizoma] 343g, Astragalus mongholicus Bunge [Fabaceae, Astragali radix] 513g, Lonicera japonica Thunb. [Caprifoliaceae, Lonicerae japonicae flos] 2058g |
| Zhang 2003 (2) | Jinqi Jiangtang Tablets: Coptis chinensis Franch. [Ranunculaceae, Coptidis rhizoma] 343g, Astragalus mongholicus Bunge [Fabaceae, Astragali radix] 513g, Lonicera japonica Thunb. [Caprifoliaceae, Lonicerae japonicae flos] 2058g |
| Dai 2005 | Qiju Dihuang Pills: Lycium barbarum L. [Solanaceae, Lycii fructus] 40g, Chrysanthemum × morifolium (Ramat.) Hemsl. [Asteraceae, Chrysanthemi flos] 40g, Rehmannia glutinosa (Gaertn.) DC. [Orobanchaceae, Rehmanniae radix praeparata] 160g, Cornus officinalis Siebold & Zucc. [Cornaceae, Corni fructus] 80g, Paeonia × suffruticosa Andrews [Paeoniaceae, Moutan cortex] 60g, Dioscorea oppositifolia L. [Dioscoreaceae, Dioscoreae rhizoma] 80g, Poria cocos(Schw.)Wolf Poria [Polyporaceae, Poria] 60g, Alisma plantago-aquatica subsp. orientale (Sam.) Sam. [Alismataceae, Alismatis rhizoma] 60g |
| Wang 2012 (1) | Xiaoke Pills: Pueraria montana var. lobata (Willd.) Maesen & S.M.Almeida ex Sanjappa & Predeep [Fabaceae, Puerariae lobatae radix] 265g, Rehmannia glutinosa (Gaertn.) DC. [Orobanchaceae, Rehmanniae Radix] 159g, Astragalus mongholicus Bunge [Fabaceae, Astragali radix] 53g, Trichosanthes kirilowii Maxim. [Cucurbitaceae, Trichosanthis radix] 265g, Zea mays L. [Poaceae, corn silk] 265g, Schisandra chinensis (Turcz.) Baill. [Schisandraceae, Schisandrae chinensis fructus] 53g, Dioscorea oppositifolia L. [Dioscoreaceae, Dioscoreae rhizoma] 26.5g, Glibenclamide 0.25g |
| Zhong 2017 | Xiaoke Pills: Pueraria montana var. lobata (Willd.) Maesen & S.M.Almeida ex Sanjappa & Predeep [Fabaceae, Puerariae lobatae radix] 265g, Rehmannia glutinosa (Gaertn.) DC. [Orobanchaceae, Rehmanniae Radix] 159g, Astragalus mongholicus Bunge [Fabaceae, Astragali radix] 53g, Trichosanthes kirilowii Maxim. [Cucurbitaceae, Trichosanthis radix] 265g, Zea mays L. [Poaceae, corn silk] 265g, Schisandra chinensis (Turcz.) Baill. [Schisandraceae, Schisandrae chinensis fructus] 53g, Dioscorea oppositifolia L. [Dioscoreaceae, Dioscoreae rhizoma] 26.5g, Glibenclamide 0.25g |
| Zhao 2021 | Xuefu Zhuyu Pills: Bupleurum chinense DC. [Apiaceae, BUPLEURI RADIX] 50g, Angelica sinensis (Oliv.) Diels [Apiaceae, Angelicae sinensis radix] 150g, Rehmannia glutinosa (Gaertn.) DC. [Orobanchaceae, Rehmanniae Radix] 150g, Paeonia lactiflora Pall. [Paeoniaceae, Paeoniae radix rubra] 100g, Carthamus tinctorius L. [Asteraceae, Carthami flos] 150g, Prunus persica (L.) Batsch [Rosaceae, Persicae semen] 200g, Citrus × aurantium L. [Rutaceae, Aurantii fructus] 100g, Glycyrrhiza glabra L. [Fabaceae, Glycyrrhizae radix et rhizoma] 50g, Conioselinum anthriscoides 'Chuanxiong' [Apiaceae, Chuanxiong rhizoma] 75g, Achyranthes bidentata Blume [Amaranthaceae, Achyranthis bidentatae radix] 150g, Platycodon grandiflorus (Jacq.) A. DC. [Campanulaceae, Platycodonis radix] 75g |
| Liu 2008 | Xuezhikang Capsules: Monascus |
| Zhao 2016 (2) | Liuwei Dihuang Pills: Rehmannia glutinosa (Gaertn.) DC. [Orobanchaceae, Rehmanniae radix praeparata] 160g, Cornus officinalis Siebold & Zucc. [Cornaceae, Corni fructus] 80g, Paeonia × suffruticosa Andrews [Paeoniaceae, Moutan cortex] 60g, Dioscorea oppositifolia L. [Dioscoreaceae, Dioscoreae rhizoma] 80g, Smilax glabra Roxb. [Smilacaceae, Smilacis glabrae rhizoma] 60g, Alisma plantago-aquatica subsp. orientale (Sam.) Sam. [Alismataceae, Alismatis rhizoma] 60g |
| Traditional Chinese Medicine Extracts | |
| Cheng 2023 | Corn silk aqueous extract: Zea mays L. [Poaceae, corn silk] |
