# Supplementary material for: Mechanism of traditional Chinese medicine in elderly diabetes mellitus and a systematic review of its clinical application
Source: Front Pharmacol. 2024 Mar 6;15:1339148. doi: 10.3389/fphar.2024.1339148 (PMC10953506; doi:10.3389/fphar.2024.1339148)
Supplement: Supplementary file 2 [file DataSheet1.zip › Supplementary Table S1-17/Supplementary Table S3a.docx]

Supplementary Table S3a | Frequency of Traditional Chinese Medicine for the treatment of elderly diabetes and islet function in Traditional Chinese Prescription.

| **Traditional Chinese Medicine** | **Frequency** |
| --- | --- |
| Astragalus mongholicus Bunge [Fabaceae, Astragali radix] | 20 |
| Dioscorea oppositifolia L. [Dioscoreaceae, Dioscoreae rhizoma] | 18 |
| Pueraria montana var. lobata (Willd.) Maesen & S.M.Almeida ex Sanjappa & Predeep [Fabaceae, Puerariae lobatae radix] | 13 |
| Coptis chinensis Franch. [Ranunculaceae, Coptidis rhizoma] | 12 |
| Rehmannia glutinosa (Gaertn.) DC. [Orobanchaceae, Rehmanniae Radix] | 12 |
| Cornus officinalis Siebold & Zucc. [Cornaceae, Corni fructus] | 10 |
| Poria cocos Schw.) olf Poria [Polyporaceae, Poria] | 10 |
| Alisma plantago-aquatica subsp. orientale (Sam.) Sam. [Alismataceae, Alismatis rhizoma] | 8 |
| Glycyrrhiza glabra L. [Fabaceae, Glycyrrhizae radix et rhizoma] | 8 |
| Ophiopogon japonicus (Thunb.) Ker Gawl. [Asparagaceae, Ophiopogonis radix] | 8 |
| Anemarrhena asphodeloides Bunge [Asparagaceae, Anemarrhenae rhizoma] | 7 |
| Paeonia × suffruticosa Andrews [Paeoniaceae, Moutan cortex] | 7 |
| Rehmannia glutinosa (Gaertn.) DC. [Orobanchaceae, Rehmanniae radix praeparata] | 7 |
| Salvia miltiorrhiza Bunge [Lamiaceae, Salviae miltiorrhizae radix et rhizoma] | 7 |
| Scutellaria baicalensis Georgi [Lamiaceae, Scutellariae radix] | 6 |
| Trichosanthes kirilowii Maxim. [Cucurbitaceae, Trichosanthis radix] | 6 |
| Polygonatum sibiricum Redouté [Asparagaceae, Polygonati rhizoma] | 5 |
| Rheum palmatum L. [Polygonaceae, Rhei radix et rhizoma] | 5 |
| Schisandra chinensis (Turcz.) Baill. [Schisandraceae, Schisandrae chinensis fructus] | 5 |
| Atractylodes macrocephala Koidz. [Asteraceae, Atractylodis macrocephalae rhizoma] | 4 |
| Codonopsis pilosula (Franch.) Nannf. [Campanulaceae, Codonopsis radix] | 4 |
| Pseudostellaria heterophylla (Miq.) Pax [Caryophyllaceae, Pseudostellariae radix] | 4 |
| Gallus gallus domesticus Brisson [Phasianidae, Galli gigerii endothelium corneum] | 3 |
| Lycium barbarum L. [Solanaceae, Lycii fructus] | 3 |
| Panax notoginseng (Burkill) F.H.Chen [Araliaceae, Notoginseng radix et rhizoma] | 3 |
| Asparagus cochinchinensis (Lour.) Merr. [Asparagaceae, Asparagi radix] | 2 |
| Atractylodes lancea (Thunb.) DC. [Asteraceae, Atractylodis rhizoma] | 2 |
| Citrus reticulata Blanco [Rutaceae, Citri reticulatae pericarpium] | 2 |
| Coix lacryma-jobi var. ma-yuen (Rom.Caill.) Stapf [Poaceae, Coicis semen] | 2 |
| Crataegus pinnatifida Bunge [Rosaceae, Crataegi fructus] | 2 |
| Gypsum fibrosum | 2 |
| Ligustrum lucidum W.T.Aiton [Oleaceae, Ligustri lucidi fructus] | 2 |
| Ophiopogon japonicus (Thunb.) Ker Gawl. [Asparagaceae, Ophiopogonis radix] | 2 |
| Paeonia lactiflora Pall. [Paeoniaceae, Paeoniae radix alba] | 2 |
| Panax ginseng C.A.Mey. [Araliaceae, Ginseng radix et rhizoma] | 2 |
| Prunus persica (L.) Batsch [Rosaceae, Persicae semen] | 2 |
| Scrophularia ningpoensis Hemsl. [Scrophulariaceae, Scrophulariae radix] | 2 |
| Sinapis alba L. [Brassicaceae, Sinapis semen] | 2 |
| Whitmania pigra Whitman [Hirudinidae, Hirudo] | 2 |
| Achyranthes bidentata Blume [Amaranthaceae, Achyranthis bidentatae radix] | 1 |
| Aloe ferox Mill. [Asphodelaceae, Aloe] | 1 |
| Anemarrhena asphodeloides Bunge [Asparagaceae, Anemarrhenae rhizoma] | 1 |
| Angelica sinensis (Oliv.) Diels [Apiaceae, Angelicae sinensis radix] | 1 |
| Bambusa tuldoides Munro [Poaceae, Bambusae caulis in taenias] | 1 |
| Bassia scoparia (L.) A.J.Scott [Amaranthaceae, Kochiae fructus] | 1 |
| Cinnamomum verum J.Presl [Lauraceae, Cinnamomi cortex]] | 1 |
| Citrus × aurantium L. [Rutaceae, Aurantii fructus immaturus] | 1 |
| Citrus × aurantium L. [Rutaceae, Aurantii fructus] | 1 |
| Conioselinum anthriscoides 'Chuanxiong' [Apiaceae, Chuanxiong rhizoma] | 1 |
| Cryptotympana pustulata Fabricius [Cicadidae, Cicadae periostracum] | 1 |
| Cullen corylifolium (L.) Medik. [Fabaceae, Psoraleae fructus] | 1 |
| Dendrobium nobile Lindl. [Orchidaceae, Dendrobii caulis] | 1 |
| Epimedium sagittatum (Siebold & Zucc.) Maxim. [Berberidaceae, Epimedii folium] | 1 |
| Eucommia ulmoides Oliv. [Eucommiaceae, Eucommiae cortex] | 1 |
| Euphorbia humifusa Willd. [Euphorbiaceae, Euphorbiae humifusae herba] | 1 |
| Liquidambar formosana Hance [Altingiaceae, Liquidambaris fructus] | 1 |
| Lobelia chinensis Lour. [Campanulaceae, Lobeliae chinensis herba] | 1 |
| Lonicera japonica Thunb. [Caprifoliaceae, Lonicerae japonicae flos] | 1 |
| Lycium barbarum L. [Solanaceae, Lycii cortex] | 1 |
| Natrii sulfas exsiccatus, Glycyrrhiza glabra L. [Fabaceae, Glycyrrhizae radix et rhizoma] | 1 |
| Nelumbo nucifera Gaertn. [Nelumbonaceae, Nelumbinis folium] | 1 |
| Neolitsea cassia (L.) Kosterm. [Lauraceae, Cinnamomi ramulus] | 1 |
| Paeonia lactiflora Pall. [Paeoniaceae, Paeoniae radix alba] | 1 |
| Pheretima aspergillum (E.Perrier) [Megascolecidae, Pheretima] | 1 |
| Pinellia ternata (Thunb.) Makino [Araceae, Pinelliae rhizoma] | 1 |
| Polygonatum odoratum (Mill.) Druce [Asparagaceae, Polygonati odorati rhizoma] | 1 |
| Polygonatum sibiricum Redouté [Asparagaceae, Polygonati rhizoma] | 1 |
| Prunus mume (Siebold) Siebold & Zucc. [Rosaceae, Mume fructus] | 1 |
| Scrophularia ningpoensis Hemsl. [Scrophulariaceae, Scrophulariae radix] | 1 |
| Scutellaria barbata D.Don [Lamiaceae, Scutellariae barbatae herba] | 1 |
| Trichosanthes kirilowii Maxim. [Cucurbitaceae, Trichosanthis fructus] | 1 |
| Trichosanthes kirilowii Maxim. [Cucurbitaceae, Trichosanthis radix] | 1 |
| Zingiber officinale Roscoe [Zingiberaceae, Zingiberis rhizoma recens] | 1 |
| Zingiber officinale Roscoe [Zingiberaceae, Zingiberis rhizoma] | 1 |
