# Supplementary material for: Mechanism of traditional Chinese medicine in elderly diabetes mellitus and a systematic review of its clinical application
Source: Front Pharmacol. 2024 Mar 6;15:1339148. doi: 10.3389/fphar.2024.1339148 (PMC10953506; doi:10.3389/fphar.2024.1339148)
Supplement: Supplementary file 2 [file DataSheet1.zip › Supplementary Table S1-17/Supplementary Table S3b.docx]

Supplementary Table S3b | Frequency of Traditional Chinese Medicine for the treatment of elderly diabetes and islet function in Traditional Chinese patent medicines.

| Traditional Chinese Medicine | Frequency |
| --- | --- |
| Rehmannia glutinosa (Gaertn.) DC. [Orobanchaceae, Rehmanniae Radix] | 16 |
| Alisma plantago-aquatica subsp. orientale (Sam.) Sam. [Alismataceae, Alismatis rhizoma] | 12 |
| Dioscorea oppositifolia L. [Dioscoreaceae, Dioscoreae rhizoma] | 12 |
| Astragalus mongholicus Bunge [Fabaceae, Astragali radix] | 11 |
| Poria cocos(Schw.)Wolf Poria [Polyporaceae, Poria] | 11 |
| Ophiopogon japonicus (Thunb.) Ker Gawl. [Asparagaceae, Ophiopogonis radix] | 10 |
| Schisandra chinensis (Turcz.) Baill. [Schisandraceae, Schisandrae chinensis fructus] | 10 |
| Cornus officinalis Siebold & Zucc. [Cornaceae, Corni fructus] | 9 |
| Trichosanthes kirilowii Maxim. [Cucurbitaceae, Trichosanthis radix] | 9 |
| Paeonia × suffruticosa Andrews [Paeoniaceae, Moutan cortex] | 8 |
| Pueraria montana var. lobata (Willd.) Maesen & S.M.Almeida ex Sanjappa & Predeep [Fabaceae, Puerariae lobatae radix] | 8 |
| Coptis chinensis Franch. [Ranunculaceae, Coptidis rhizoma] | 6 |
| Lycium barbarum L. [Solanaceae, Lycii fructus] | 5 |
| Rehmannia glutinosa (Gaertn.) DC. [Orobanchaceae, Rehmanniae radix praeparata] | 5 |
| Panax ginseng C.A.Mey. [Araliaceae, Total ginsenoside of ginseng stems and leaves] | 4 |
| Panax ginseng C.A.Mey. [Araliaceae, Ginseng radix et rhizoma] | 4 |
| Rubus chingii Hu [Rosaceae, Rubi fructus] | 4 |
| Anemarrhena asphodeloides Bunge [Asparagaceae, Anemarrhenae rhizoma] | 3 |
| Atractylodes lancea (Thunb.) DC. [Asteraceae, Atractylodis rhizoma] | 3 |
| Epimedium sagittatum (Siebold & Zucc.) Maxim. [Berberidaceae, Epimedii folium] | 3 |
| Eupatorium fortunei Turcz. [Asteraceae, Eupatorii herba] | 3 |
| Glycyrrhiza glabra L. [Fabaceae, Glycyrrhizae radix et rhizoma] | 3 |
| Litchi chinensis Sonn. [Sapindaceae, Litchi semen] | 3 |
| Lonicera japonica Thunb. [Caprifoliaceae, Lonicerae japonicae flos] | 3 |
| Lycium barbarum L. [Solanaceae, Lycii cortex] | 3 |
| Polygonatum sibiricum Redouté [Asparagaceae, Polygonati rhizoma] | 3 |
| Reynoutria multiflora (Thunb.) Moldenke [Polygonaceae, Polygoni multiflori radix] | 3 |
| Salvia miltiorrhiza Bunge [Lamiaceae, Salviae miltiorrhizae radix et rhizoma] | 3 |
| Smilax glabra Roxb. [Smilacaceae, Smilacis glabrae rhizoma] | 3 |
| Sophora flavescens Aiton [Fabaceae, Sophorae flavescentis radix] | 3 |
| Zea mays L. [Poaceae, corn silk] | 3 |
| Achyranthes bidentata Blume [Amaranthaceae, Achyranthis bidentatae radix] | 2 |
| Cuscuta chinensis Lam. [Convolvulaceae, Cuscutae semen] | 2 |
| Pseudostellaria heterophylla (Miq.) Pax [Caryophyllaceae, Pseudostellariae radix] | 2 |
| Whitmania pigra Whitman [Hirudinidae, Hirudo] | 2 |
| Aconitum carmichaelii Debeaux [Ranunculaceae, Aconiti lateralis radix praeparata] | 1 |
| Angelica sinensis (Oliv.) Diels [Apiaceae, Angelicae sinensis radix] | 1 |
| Bupleurum chinense DC. [Apiaceae, BUPLEURI RADIX] | 1 |
| Carthamus tinctorius L. [Asteraceae, Carthami flos] | 1 |
| Chrysanthemum × morifolium (Ramat.) Hemsl. [Asteraceae, Chrysanthemi flos] | 1 |
| Citrus × aurantium L. [Rutaceae, Aurantii fructus] | 1 |
| Conioselinum anthriscoides 'Chuanxiong' [Apiaceae, Chuanxiong rhizoma] | 1 |
| Monascus | 1 |
| Paeonia lactiflora Pall. [Paeoniaceae, Paeoniae radix rubra] | 1 |
| Plantago asiatica L. [Plantaginaceae, Plantaginis semen] | 1 |
| Platycodon grandiflorus (Jacq.) A.DC. [Campanulaceae, Platycodonis radix] | 1 |
| Prunus mume (Siebold) Siebold & Zucc. [Rosaceae, Mume fructus] | 1 |
| Prunus persica (L.) Batsch [Rosaceae, Persicae semen] | 1 |
