# Supplementary material for: Mechanism of traditional Chinese medicine in elderly diabetes mellitus and a systematic review of its clinical application
Source: Front Pharmacol. 2024 Mar 6;15:1339148. doi: 10.3389/fphar.2024.1339148 (PMC10953506; doi:10.3389/fphar.2024.1339148)
Supplement: Supplementary file 2 [file DataSheet1.zip › Supplementary Table S1-17/Supplementary Table S4.docx]

Supplementary Table S4 | TCM for elderly DKD.

| Study | Subjects (Age range) | Ages^1^ | No. of intervention group/control group | Treatment of intervention group^2^ | Dose of intervention group | Treatment of control group | Dose of control group | Duration | Outcomes^3^ | Adverse reactions |
| --- | --- | --- | --- | --- | --- | --- | --- | --- | --- | --- |
| Traditional Chinese Prescription | | | | | | | | | | |
| Chen 2016 | The poor effect of conventional western medicine on elderly DKD (stage Ⅲ and Ⅳ, ≥60 years old) | control group:64.1±2.9 years old; intervention group: 65.7±2.5 years old | 40/40 | Herbal Paste+Conventional Western Medicine Treatment | 15g, 2/d | Conventional Western Medicine Treatment | NM | 4 months | UACR, UAER, eGFR, 24h-UTP | NM |
| Li 2018 (1) | Elderly DKD (55-75 years old) | control group: 60.4 ± 3.2 years old; intervention group: 61.2 ± 2.2 years old | 45/45 | Huangqi Guizhi Wuwu Decoction+Conventional Western Medicine Treatment | 1 dose, 3/d | Conventional Western Medicine Treatment | NM | 18 weeks | UAER, VEGF, mRNM, TGF-β | NM |
| Zhao 2016 (3) | Early DKD (stage Ⅲ) in the Elderly | 67.5±7.2 years old | 32/32 | Huangqi Guizhi Wuwu Decoction+Insulin | 1 dose, 3/d | insulin | 18-24U, 3/d | 12 weeks | FBG, 2hPBG, HbA1c, Cr, urine-ALB, UTP | NM |
| Nie 2015 | Elderly DKD (stage Ⅲ and Ⅳ, 61-79 years old) | control group: 66.24 ± 3.57 years old; intervention group: 66.60 ± 4.41 years old | 50/50 | Jianshenling capsules+Irbesartan | 5 capsules, 3/d | Irbesartan | 150mg, 1/d | 3 months | hs-CRP, Cr, ALB | NM |
| Jin 2019 | Elderly DKD | control group: 68.34±6.22 years old; intervention group: 70.15±7.68 years old | 40/40 | Jinchanhua Decoction+Losartan | 1 dose, 2/d | Losartan | 100mg, 1/d | 16 weeks | Cys-C, Cr^*^, ALB, UACR | NM |
| Feng 2015 | Elderly DKD (40-64 years old) | control group: 46.7 ± 4.0 years old; intervention group: 46.3 ± 3.8 years old | 60/60 | Liuwei Dihuang Decoction+Sodium Ferulate Injection | 150ml, 2/d | Sodium Ferulate Injection | 0.3g, 1/d | 3 months | FBG^*^, 2hPBG^*^, HbA1c^*^, UAER, Ccr, TC^*^, TG^*^, HDL^*^, LDL^*^ | NM |
| Chen 2015 | Early DKD (stage Ⅲ) in the Elderly (≥65 years old) | control group: 72.5 ± 8.3 years old; intervention group: 72.2 ± 8.1 years old | 82/82 | Pingtang Gushen Recipe+Irbesartan | 150ml, 3/d | Irbesartan | 150mg, 1/d | 12 weeks | UAER, Cr, BUN, FBG, HbA1c, TC, TG, HDL, LDL | no adverse reaction |
| Ou 2011 | Elderly DKD (stage Ⅲ and Ⅳ, ≥65 years old) | control group: 80.1±6.6 years old; intervention group: 77.9±9.8 years old | 40/40 | Self-treating renal stasis Tongfu Decoction+losartan+Coated Aldehyde Oxystarch Capsules | 250ml, 2/d | Coated Aldehyde Oxystarch Capsules+losartan | Coated Aldehyde Oxystarch Capsules: 5-8 capsules, 3/d; losartan: 50mg, 1/d | 4 months | UAER, Cr, BUN, UA | NM |
| Li 2023 | Elderly DKD (stage Ⅲ,≥65 years old) | control group: 72.35±4.89 years old; intervention group: 72.26±4.14 years old | 51/51 | Shenqi Buyi Prescription with Xiaoke Prescription+Conventional Western Medicine Treatment | 200ml, 2/d | Conventional Western Medicine Treatment | NM | 12 weeks | FBG, 2hPBG, HbA1c, 24hUTP, urine-β2MG, HOMA-IR, FINS, HOMA-IS, TGF-β1, MMP9, TIMP1 | NM |
| Hu 2018 (1) | Elderly DKD | control group: 67.55±7.48 years old; intervention group: 69.04±7.53 years old | 34/34 | Si Mo Yin Zi+Irbesartan | 1 dose, 2/d | Lrbesartan | 150mg, 1/d | 4 months | MMSE, BDNF, Hcy, urine-α1 MG, Cr, BUN, UA, Ccr | control group: diarrhea (2), dizzy (2); intervention group: dizzy (2), gastrointestinal discomfort (1) |
| Lin 2022 | Elderly T2DM (≥60 years old) | control group: 64.45±3.13 years old; intervention group: 65.62±3.19 years old | 36/36 | Traditional Chinese Medicine decoction+Conventional Western Medicine Treatment | 1 dose, 2/d | Conventional Western Medicine Treatment | NM | 24 months | UACR, Cr, Ccr, β2-MG | NM |
| Yang 2021 (1) | Elderly DKD (≥60 years old) | control group: 61-84 years old; intervention group:60-85 years old | 55/55 | Wenyang Jiangzhuo Tongluo Recipe+Calcium dobesilate | 150ml, 2/d | Calcium dobesilate | 0.5g, 3/d | 4 months | FBG, TC, HbA1c, UAER | NM |
| Li 2019 | Elderly DKD with deficiency of both qi and yin syndrome (≥60 years old) | control group: 67±9.74 years old; intervention group: 68±10.02 years old | 50/50 | Yigi Yangyin Decoction+Conventional Western Medicine Treatment | 150ml, 2/d | Conventional Western Medicine Treatment | NM | 3 months | BUN, Cr, 24h-UTP, UAER | NM |
| Su 2019 | Elderly T2DM (60-80 years old) | control group: 66.21±2.98 years old; intervention group: 66.62±3.19 years old | 43/43 | Yiqi Guben Decoction+Recombinant insulin glargine Injection | 1 dose, 2/d | Recombinant insulin glargine | Adjust dosage based on blood sugar levels, 1/d | 24 months | incidence rate, UACR, Cr, Ccr, β2-MG | NM |
| Wang 2022 (2) | Elderly T2DM (≥60 years old) | control group: 66.21 ± 2.98 years old; intervention group: 66.62 ± 3.19 years old | 30/30 | Yiqi Guben Decoction+Recombinant insulin glargine Injection | 1 dose, 2/d | Recombinant insulin glargine | Adjust dosage based on blood sugar levels, 1/d | 24 months | incidence rate, UACR, Cr, Ccr, β2-MG | NM |
| Jiang 2019 | Early DKD (stage Ⅲ) in the Elderly (≥60 years old) | control group: 65.73 ± 4.36 years old; intervention group: 66.71 ± 4.42 years old | 44/44 | Yiqi Yangyin Decoction+Atorvastatin calcium+Metformin | Atorvastatin calcium: 10mg, 1/d; Yiqi Yangyin Decoction: 50ml, 2/d | Metformin | 0.5g, 2/d | 3 months | UAER, Cr, β2-MG, SCD40L, ET | control group: nausea (2), diarrhea (1); intervention group: abdominal distension (2), nausea (1), diarrhea (2) |
| Feng 2023 | Elderly DKD (stage Ⅲ and Ⅳ, ＞60 years old) | control group: 69.21 ± 4.25 years old; intervention group: 70.03 ± 4.51 years old | 48/53 | Yishen Paidu Formula+Conventional Western Medicine Treatment | 200ml, 2/d | Conventional Western Medicine Treatment | NM | 12 weeks | IL-6, hs-CRP, Cr, BUN, 24hUTP, β2-MG | control group: vomit (4), loss of appetite (3), fever (1), rash (2); intervention group: vomit (3), loss of appetite (5), fever (2), rash (3) |
| Gao 2010 | Elderly DKD (≥60 years old) | control group: 75.00±2.87 years old; intervention group: 75.00±3.96 years old | 43/43 | Yishen Tangshi'an Recipe+Iotensin or Valsartan | 1 dose, 2/d | Benazepril or Valsartan | lotensin: 10mg, 1/d; Valsartan: 80mg, 1/d | 3 months | UAER, urine-β2 MG^*^, HbA1c^*^, Cr^*^, BUN^*^, TG*, TC | no adverse reaction |
| Wen 2006 | Elderly DKD (stage Ⅲ and Ⅳ) | control group: 62.10±7.3 years old; intervention group: 63.50±6.9 years old | 50/48 | Zhenqing Prescription+Benazepril | 50ml, 3/d | Benazepril | 10mg, 1/d | 3 months | FBG, TG, TC, LDL, HDL, VLDL, UAER, 24h-UTP, BUN, Cr | NM |
| Zhang 2022 | Early and middle (stage Ⅲ and Ⅳ) DKD with Yang Qi Deficiency and Blood Stasis syndrome in elderly | control group: 55. 94±8. 12 years old; intervention group:56. 02±7. 59 years old | 65/61 | Zhenwu Decoction+Losartan Potassium Tablets | 1 dose, 2/d | Losartan Potassium Tablets | 100mg, 1/d | 3 months | UAER, 24h-UTP, BUN, Cr, TLR4, MyD88, NF-κB | NM |
| Zhang 2021 | Elderly DKD of Qi and Yin deficiency combined with blood stasis syndrome (≥60 years old) | control group: 62.65 ± 8.11 years old; intervention group: 61.97 ± 8.02 years old | 56/56 | Zicui Yishen Decoction+Conventional Western Medicine Treatment | 100ml, 2/d | Conventional Western Medicine Treatment | NM | 3 months | FBG, 2hPBG, HbA1c, Cr, BUN, 24h-UTP, WBV, PV, NLRP3, VEGF | no adverse reaction |
| Traditional Chinese patent medicines | | | | | | | | | | |
| Fang 2019 | Elderly DKD (65-82 years old) | control group: 72.75±5.56 years old; intervention group: 70.29±6.15 years old; combination therapy group: 71.67±5.87 years old | 45/45 | (1) Bailing Capsule^†^+Enalapril; (2) Bailing Capsules | 1g, 3/d | Enalapril | 25mg, 2/d | 3 months | FBG, 2hPBG, FINS, HOMA-IR, TC, TG, 24h-UTP, CRP, β2-MG, NMG, ALB, Cr, BUN | NM |
| Yu 2021 | Early DKD stage Ⅲ in the elderly | control group: 70.3±7.0 years old; intervention group: 61.8±6.7 years old; combination therapy group: 68.2±7.2 years old | 64/64 | (1) Bailing Capsule^†^+Pancreatic Kininogenase; (2) Bailing Capsule | Bailing Capsule: 1.5g, 3/d; Pancreatic Kininogenase: 240U, 3/d | Pancreatic Kininogenase | 240U, 3/d | 16 weeks | FBG*, 2hPBG*, HbA1c*, RBP, Cys-C, Hcy, NMG | control group: rash (1); intervention group: none |
| Wang 2018 (1) | Elderly DKD (＞60 years old) | 72.98 ± 18.19 years old | 30/30/30 | (1) Olmesartan; (2) Shenyan Kangfu Tablet^†^+olmesartan | (1) olmesartan: 20mg, 1/d; (2) Shenyan Kangfu Tablet: 2.4g, 3/d | Conventional Western Medicine Treatment | NM | 12 weeks | BUN^*^, Cr, UA, ALB, UTP^*^ | NM |
| Shen 2013 | Elderly DKD (stage Ⅲ and Ⅳ, ≥60 years old) | 66.5±5.5 years old | 34/34 | Bailing Capsules^†^+Atorvastatin calcium | 2g, 3/d | Atorvastatin calcium | 20mg, 1/d | 16 weeks | TC, TG, LDL, 24h-UTP, hs-CRP, Cr, UA | no adverse reaction |
| Wang 2016 | Early DKD (＜stage Ⅲ) in the Elderly (61-78 years old) | control group: 69.7±6.7 years old; intervention group: 68.8±6.2 years old | 50/50 | Bailing capsules^†^+Captopril | 5 pills, 3/d | Captopril | 2 tablets, 2/d | 6 months | UAER, Cr, BUN, TC^*^, TG^*^, LDL^*^, HDL^*^, FBG^*^, HbA1c^*^, CRP | no adverse reaction |
| Xu 2022 | Early DKD (stage Ⅱ and Ⅲ) in the elderly (80-94 years old) | control group: 85. 21±4. 03 years old; intervention group: 85. 19±4. 07 years old | 50/50 | Bailing Capsules^†^+Conventional Western Medicine Treatment | 2g, 3/d | Conventional Western Medicine Treatment | NM | 12 weeks | BUN, U-mALB, Cr, hs-CRP, IL-6, IL-1, TNF-α, GSH-Px, MDA, SOD | control group: pharyngeal discomfort (1), hypotension (2), electrolyte disturbance (2); intervention group: pharyngeal discomfort (1), hypotension (2), electrolyte disturbance (3) |
| Peng 2013 | Elderly DKD (stage Ⅲ, 60-81 years old) | 71±9.9 years old | 30/30 | Bailing Capsules^†^+Conventional Western Medicine Treatment | 1g, 3/d | Conventional Western Medicine Treatment | NM | 12 weeks | ALB, β2-MG, NMG, FBG*, TC*, TG* | NM |
| Yang 2016 | Elderly DKD (63-78 years old) | control group: 71.4±6.6 years old; intervention group: 72.3±7.2 years old | 45/45 | Bailing capsules^†^+Enalapril | 1g, 3/d | Enalapril | 5mg, 2/d | 12 weeks | mALB, Cr, BUN, IL-1, IL-6 | NM |
| Sun 2012 (1) | Elderly DKD (68-88 years old) | control group: 72.0±1.5 years old; intervention group: 75.6±4.6 years old | 45/45 | Bailing capsules^†^+Enalapril | 1g, 3/d | Enalapril | 5mg, 2/d | 12 weeks | SBP, DBP, 24h-UTP, β2-MG, Cr, BUN | NM |
| Hong 2010 | Elderly DKD (stage Ⅲ and Ⅳ, 52-72 years old) | control group: 65.80±5.0 years old; intervention group: 66.20±5.4 years old | 30/30 | Bailing Capsules^†^+Irbesartan | 5 capsules, 3/d | Irbesartan | 150mg, 1/d | 16 weeks | 24h-UTP, SBP^*^, DBP^*^, Cr, BUN | NM |
| Wang 2007 | Early DKD (stage Ⅲ) in the Elderly | control group: 64.60±2.4 years old; intervention group: 65.60±1.6 years old | 50/48 | Bailing Capsules^†^+Losartan | 3g, 3/d | Losartan | 100mg, 1/d | 16 weeks | Cr, K, UAER | no adverse reaction |
| Shen 2021 | Early DKD in the elderly (60-75 years old) | control group: 67. 08±5. 18 years old; intervention group: 67. 16 ± 4. 46 years old | 40/40 | Bailing Tablets+Acarbose+Insulin Aspart Injection+Metformin+Benazepril | 0.9g, 3/d | Acarbose+Insulin Aspart Injection+Metformin+Benazepril | Acarbose: 50mg, 3/d; Metformin:0.5g, 2/d; Benazepril: 5-20mg, 1/d; Insulin Aspart Injection | 12 weeks | BUN, Cr, urine-ALB, hs-CRP, TNF-α, IL-6, SOD, MDA, T-AOC | control group: abdominal distension (5), hypoglycemia (2), rash (1), diarrhea (2); intervention group: abdominal distension (3), hypoglycemia (1), rash (1), diarrhea (3) |
| Wang 2022 (3) | Early DKD (＜stage Ⅲ) in the elderly (≥55 years old) | control group: 63.8±8.3 years old; intervention group: 64.9±9.2 years old | 46/46 | Compound Danshen Dripping Pills^†^+Irbesartan | 1 pills, 3/d | irbesartan | 150mg, 1/d | 12 weeks | BUN, Cr, Ccr, TC, TG, mALB, WBV, PV, HCT | control group: dizzy (1), loss of appetite (1), nausea (1); intervention group: dizzy (1), loss of appetite (0), nausea (0) |
| BAI 2008 | Early DKD (stage Ⅲ) in the Elderly | 66.0±5.0 years old | 48/48 | Compound Danshen Dripping Pills^†^+Irbesatan | 10 capsules, 3/d | Irbesartan | 150mg, 1/d | 16 weeks | WBV, PV, FIB, HCT, TC, TG, HDL^*^, LDL, Cr^*^, BUN^*^, 24hUTP, Ccr, UAER^*^ | no adverse reaction |
| Wang 2018 (2) | Elderly DKD (60-85 years old) | control group: 72. 91 ± 5. 27 years old; intervention group: 73. 97 ± 5. 86 years old | 51/51 | Compound Danshen Dropping Pills^†^+Conventional Western Medicine Treatment+Irbesartan | Compound Danshen Dropping Pills: 15pills, 3/d; Irbesartan: 150mg, 1/d | Conventional Western Medicine Treatment+Irbesartan | Irbesartan: 150mg, 1/d | 3 months | FBG^*^, 2hPBG^*^, urine-ALB, urine-Cr | NM |
| Xie 2016 | Elderly DKD (60-86 years old) | control group: 69.1±4.1 years old; intervention group: 72.3±3.1 years old | 63/63 | Compound Danshen Dropping Pills^†^+Fosinopril Sodium | 10 pills, 3/d | Fosinopril Sodium | NM | 3 months | UACR | NM |
| Zhu 2014 | Early DKD (stage Ⅲ) in the Elderly (50-77 years old) | control group: 62.4±5.9 years old; intervention group: 63.6±6.1 years old | 43/43 | Compound Danshen Dropping Pills^†^+Irbesartan | 10 pills, 3/d | Irbesartan | 150mg, 1/d | 12 weeks | FBG^*^, 2hPBG^*^, HbA1c^*^, UAER, TC^*^, TG^*^, HDL^*^, LDL^*^ | NM |
| Ma 2017 (2) | Elderly DKD (≥60 years old) | control group: 67.0 ± 3.8 years old; intervention group: 66.4 ± 4.2 years old | 42/42 | Compound Danshen Dropping Pills^†^+Irbesartan | 15 pills, 3/d | Irbesartan | 150mg, 1/d | 3 months | TC, HDL, LDL, Cr^*^, Ccr^*^, UAER | NM |
| Lin 2019 | Early DKD (stage Ⅲ) in the Elderly (≥60 years old) | control group: 66. 23±5. 17 years old; intervention group: 66. 91±5. 42 years old | 89/87 | Congrong Yishen Granules^†^+Conventional Western Medicine Treatment | 2g, 2/d | Conventional Western Medicine Treatment | NM | 3 months | urine-ALB, Cr, UAER, β2-MG, Cys-C, EGFR, NO, ET-1, TXB2, 6-Keto-PGF-1α, WBV, PV, WBRV, MPAR, FIB, hs-CRP, IL-6, TNF-α, IL-17A, IL-23, TGF-β1, MMP9, TIMP1, MMP9/TIMP-1 | control group: dizzy (1); intervention group: nausea (2) |
| Su 2020 (2) | Elderly DKD (60-76 years old) | 62.83±7.29 years old | 47/47 | Jinlida Granules^†^+Benazepril | 9g, 3/d | Benazepril | 10mg, 1/d | 12 weeks | FBG, 2hPBG, HbA1c, UAER, HOMA-IR, TG, TC, VEGF, IGF-1 | control group: abdominal distension (1), dizzy (2), headache (1), nausea (1); intervention group: abdominal distension (1), dizzy (0), headache (0), nausea (1) |
| Huang 2010 | Early DKD (＜stage Ⅲ) in the Elderly (≥60 years old) | control group: 71.3±11.6 years old; intervention group: 71.1±10.7 years old | 32/32 | Jinshuibao Capsule^†^+Perindopril | 1.98g, 3/d | Perindopril | 4mg, 1/d | 12 weeks | 24h-UTP, UAER, urine-α1 MG, β2-MG, FBG^*^, HbA1c^*^ | NM |
| Pan 2016 | Early DKD (＜stage Ⅲ) in the Elderly (60-75 years old) | control group: 65.7 ± 5.2 years old; intervention group: 64.5 ± 4.7 years old | 40/40 | Jinshuibao capsule^†^+Telmisartan | 3 pills, 3/d | Telmisartan | 80mg, 1/d | 3 months | urine-ALB, UACR, β2-MG, Cr, BUN, SBP^*^, DBP^*^, TG^*^, TC^*^, HDL^*^, LDL | NM |
| Huang 2014 | Early DKD (＜stage Ⅲ) in the elderly (≥65 years old) | control group: 75.2±7.6 years old; intervention group: 74.8±7.4 years old | 48/48 | Jinshuibao Capsules^†^+Compound Danshen Dropping Pillsv^†^+Irbesartan | Jinshuibao Capsules: 3 capsules, 3/d; Compound Danshen Dropping Pills: 10 pills, 3/d | Irbesartan | 150mg, 1/d | 3 months | ALT^*^, AST^*^, BUN^*^, Cr^*^, UAER, TC^*^, TG^*^, HDL^*^, LDL^*^ | NM |
| Yi 2009 | Elderly DKD (stage Ⅲ and Ⅳ, ≥60 years old) | control group 1: 65.8±5.0 years old; control group 2: 66.2±5.4 years old; intervention group: 67.0±4.8 years old | 20/20/20 | Jinshuibao Capsules^†^+Irbesartan | Irbesartan: 150mg, 1/d; Jinshuibao Capsules: 3 capsules, 3/d | Irbesartan | 75mg, 1/d; 150mg, 1/d | 12 weeks | 24h-UTP, Cr, BUN, NMG, TC, TG, HDL, LDL | NM |
| Zhong 2020 | Elderly DKD ( stage Ⅲ-Ⅴ, ≥65 years old) | control group: 72.4 ± 7.1 years old; intervention group: 71.8 ± 6.7 years old | 30/30 | Jinshuibao Tablets^†^+Conventional Western Medicine Treatment | 1.68g, 3/d | Conventional Western Medicine Treatment | NM | 12 weeks | HbA1c, ALB, Cr, BUN, 24h-UTP, MIS | NM |
| Zhang 2014 | Elderly DKD (≥stage Ⅳ, 63-81 years old) | control group: 67.3±7.4 years old; intervention group: 68.2±7.6 years old | 52/50 | Niaoduqing Granules+Conventional Western Medicine Treatment+Valsartan+Atorvastatin calcium+Bayaspirin | 5g, 4/d | Conventional Western Medicine Treatment+Valsartan+Atorvastatin calcium+Bayaspirin | Valsartan: 160mg, 1/d; Atorvastatin calcium: 10mg, 1/d; Bayaspirin: 0.1g, 1/d; | 12 weeks | IL-lβ, IL-6, CRP, TNF-α, VEGF, UAER | NM |
| Sun 2012 (2) | Elderly DKD | control group: 62.23±11.99 years old; intervention group: 62.34±12.18 years old | 45/45 | Okra Capsules+Benazepril | 5 capsules, 3/d | Benazepril | 10mg, 1/d | 12 weeks | mAlb, 24h-UTP, FBG, Cr, BUN | control group: dizzy (1), headache (2); intervention group: dizzy (1) |
| Li 2016 (1) | Early DKD (＜stage Ⅲ) in the Elderly (60-88 years old) | control group: 64.8 ± 9.8 years old; intervention group: 66.9 ± 7.1 years old | 48/48 | Okra capsules+Calcium dobesilate | 2.5g, 3/d | Calcium Dobesilate | 500mg, 3/d | 12 weeks | BUN^*^, Cr^*^, HbA1c^*^, UAER, ET, Cys-C, SBP^*^ | no adverse reaction |
| WANG 2023 | Elderly DKD (60-75 years old) | control group: 67.67±6.88 years old; intervention group: 66.31±6.21 years old | 35/36 | Qi-Kui Granules+Conventional Western Medicine Treatment | 10g, 3/d | Conventional Western Medicine Treatment+Dulaglutide Injection | 1.5mg, 1/w | 12 weeks | FBG^*^, HbA1c^*^, SBP^*^, DBP^*^, TC^*^, TG^*^, HDL^*^, LDL^*^, Cr, UACR, 24h-UTP | NM |
| Shi 2019 | Early DKD (stage Ⅲ) in the Elderly (≥65 years old) | control group: 70.1±6.9 years old; intervention group: 69.2±6.7 years old | 50/50 | Shen'an Capsules+Rosuvastatin Calcium | 0.8g, 3/d | Rosuvastatin Calcium | 5mg, 1/d | 3 months | FBG, 2hPBG, HbA1c, TC, TG, LDL, HDL, UAER, urine-ALB, UACR | NM |
| Shu 2010 | Elderly DKD (stage Ⅳ, 67-83 years old) | control group: 73±10 years old; intervention group: 75±13 years old | 40/40 | Shenyan Kangfu Tablets^†^+Conventional Western Medicine Treatment | 8 tablets, 3/d | Conventional Western Medicine Treatment | NM | 4 months | 24h-UTP, ALB^*^, TC^*^, BUN, Cr | no adverse reaction |
| Guo 2021 | Elderly DKD (56-75 years old) | control group: 52.12+5.58 years old; intervention group: 67.09±8.45 years old | 30/30 | Shenyan Kangfu Tablets^†^+Conventional Western Medicine Treatment | 8 tablets, 3/d | Conventional Western Medicine Treatment | NM | 4 months | ET, HbA1c, UAER, TC, TG | control group: dizzy (1), nausea (1), blurred vision (0); intervention group: dizzy (3), nausea (2), blurred vision (2) |
| Li 2020 | Elderly DKD (63-79 years old) | control group: 66.1±1.2 years old; intervention group: 66.1±1.5 years old | 45/45 | Shenyankang Tablets^†^+Benazepril | 1.8g, 3/d | benazepril | 80mg, 1/d | 16 weeks | BUN, Cr, 24h-UTP, IL-6, TNF-α, SBP, DBP | control group: cough (3), gastrointestinal discomfort (6), dizzy (1); intervention group: cough (2), gastrointestinal discomfort (5), dizzy (2) |
| Liu 2023 | Early DKD (≤stage Ⅲ) in the elderly (≥60 years old) | control group: 67.42±2.47 years old; intervention group: 68.13±2.51 years old | 64/64 | Yishen Huashi Granule^†^+Dapagliflozin+Insulin Aspart Injection | 10g, 3/d | Dapagliflozin+Insulin Aspart Injection | Dapagliflozin: 10mg,1/d; Insulin Aspart Injection: 0.2U/kg, 3/d | 3 months | FBG, 2hPBG, LAGE, PPGE, BUN, Cr, 24h-UTP, UAER, GSK-3β, LXA4, hs-CRP, IL-6 | control group: hypoglycemia (3); intervention group: hypoglycemia (2) |
| HU 2018 (2) | Early DKD in the Elderly (60-85 years old) | control group: 72.5±10.2 years old; intervention group: 73.2±10.5 years old | 67/67 | Yishen Huashi Granules^†^+Sulodexide | 10g, 3/d | Sulodexide | 600LSU, 1/d | 4 months | FBG, 2hPBG, MAP, Cr, 24h-UAER, TC, TG, HDL, LDL, MDA, SOD, vWF, ET-1 | NM |
| Chen 2018 | Early DKD in the Elderly (65-80 years old) | control group: 67.55 ± 7.48 years old; intervention group: 69.04 ± 7.53 years old | 70/70 | Yishen Huashi Granules^†^+Sulodexide | 10g, 2/d | Sulodexide | 250LSU, 2/d | 12 weeks | Cr, BUN, ALB, UAER, 24h-UTP, TC, TG, LDL, HDL, MDA, SOD, vWF, ET-1 | control group: dizzy (1), headache (2), dry (1), rash (0); intervention group: dizzy (3), headache (1), dry (1), rash (1) |
| Hu 2016 | Early DKD (＜stage Ⅲ) in the Elderly (65-80 years old) | control group: 72.41 ± 5.85 years old; intervention group: 72.56 ±5.90 years old | 45/41 | Yishen Huashi Granules^†^+Sulodexide Injection | 10g, 3/d | Sulodexide Injection | Sulodexide Injection: 600LSU,1/d; After 2 weeks, Sulodexide capsules: 2 capsules, 2/d | 4 months | FBG, 2hPBG, 24h-UAER, Cr, MDA, SOD, vMF, ET-1 | control group: headache (1), dizzy (1), dry (1); intervention group: headache (1), dizzy (2), dry (1) |
| Traditional Chinese Medicine Extracts | | | | | | | | | | |
| Chen 2022 | Early DKD (＜stage Ⅲ) in the elderly (65-80 years old) | control group: 70. 89 ± 4. 29 years old; intervention group: 71. 32±4. 53 years old | 41/41 | Haikunshenxi capsule+Valsartan Dispersible Tablets | 0.44g, 3/d | Valsartan Dispersible Tablets | 80mg, 1/d | 12 weeks | hs-CRP, TNF-α, IL-6, TGF-β1, MMP-2, BUN, Cr, β2-MG, 24h-UTP | NM |

^1^ Ages were displayed as mean ± standard deviation or mean.

^2^ “^†^” indicated that it was included in Pharmacopoeia of the People's Republic of China 2020.

^3^ “*” showed no significant difference between the intervention group and the control group.

Abbreviation: NM: Not mentioned; UAER: Urinary albumin excretion rate, UTP: Urine-proteinuria, 2hPBG: 2-hour Postprandial blood glucose, ALB: Albumin, ALT: Alanine aminotransferase, AST: Aspartate aminotransferase, BDNF: Serum brain derived growth factor, BUN: Blood urea nitrogen, CCr: Creatinine clearance, Cr: creatinine, CRP: C-reaction protein, Cys-C: Cystatin C, DBP: Diastolic pressure, EGFR: Epidermal growth, factor receptor, ET: Endothelin, FBG: Fasting blood glucose, FIB: Fibrinogen, FINS: Fasting insulin, GSH-Px: Glutathione peroxidase, GSK-3β: Glycogen synthase kinase-3β, HbA1c: Glycosylated hemoglobin, HCT: Hematocrit, Hcy: Homocysteine, HDL: High density lipoprotein, HOMA: Homeostasis model assessment, CRP: C-reaction protein, IL: Interleukin, LAGE: Largest amplitude of glycemic excursions, LDL: Low density lipoprotein, LXA4: Lipoxygenase A4, MAP: Mean arterial pressure, MDA: Malondialdehyde, MIS: Malnutrition-Inflammation Score, MMP: matrix metalloproteinases, MMSE: Minimum Mental State Examination, MPAR: Platelet aggregation rate, MyD88: Myeloid differentiation primary response protein 88, NF-κB: Nuclear factor kappa-B, NLRP3: NOD-like receptor thermal protein domain associated protein 3, PPGE: Postprandial glucose excursion, PV: Plasma viscosity, RBP: Retinol Binding Protein, SBP: Systolic pressure, SOD: Superoxide dismutase, T-AOC: Total antioxidant capacity, TC: Cholesterol, TG: Triglyceride, TGF-β: Transforming growth factor-β, TIMP1: tissue inhibitor of metal protease 1, TLR4: Toll-like receptor 4, TNF: tumor necrosis factor, TXB2: Thromboxane B2, UA: Uric acid, UACR: Urinary microalbumin creatinine ratio, UAER: urinary albumin excretion rate, urine-α1 MG: urine α-1 Microglobulin, UTP: urine-proteinuria, VEGF: Vascular endothelial growth factor, vWF: von willebrand factor, WBRV: whole blood reduced viscosity, WBV: whole blood viscosity, β2-MG: β2-Microglobulin
