# Supplementary material for: Mechanism of traditional Chinese medicine in elderly diabetes mellitus and a systematic review of its clinical application
Source: Front Pharmacol. 2024 Mar 6;15:1339148. doi: 10.3389/fphar.2024.1339148 (PMC10953506; doi:10.3389/fphar.2024.1339148)
Supplement: Supplementary file 2 [file DataSheet1.zip › Supplementary Table S1-17/Supplementary Table S5.docx]

Supplementary Table S5 | Interventional drugs composition of TCM for elderly DKD.

| Study | Interventional drugs composition |
| --- | --- |
| Traditional Chinese Prescription | |
| Chen 2016 | Herbal Paste: Astragalus mongholicus Bunge [Fabaceae, Astragali radix] 30g, Pseudostellaria heterophylla (Miq.) Pax [Caryophyllaceae, Pseudostellariae radix] 15g, Dioscorea oppositifolia L. [Dioscoreaceae, Dioscoreae rhizoma] 15g, Ophiopogon japonicus (Thunb.) Ker Gawl. [Asparagaceae, Ophiopogonis radix] 20g, Trichosanthes kirilowii Maxim. [Cucurbitaceae, Trichosanthis radix] 12g, Schisandra chinensis (Turcz.) Baill. [Schisandraceae, Schisandrae chinensis fructus] 6g, Coptis chinensis Franch. [Ranunculaceae, Coptidis rhizoma] 6g, Rehmannia glutinosa (Gaertn.) DC. [Orobanchaceae, Rehmanniae radix praeparata] 10g, Angelica sinensis (Oliv.) Diels [Apiaceae, Angelicae sinensis radix] 15g, Poria cocos (Schw.) Wolf Poria [Polyporaceae, Poria] 15g, Alisma plantago-aquatica subsp. orientale (Sam.) Sam. [Alismataceae, Alismatis rhizoma] 12g, Tetrapanax papyrifer (Hook.) K.Koch [Araliaceae, Tetrapanacis medulla] 10g, Glycyrrhiza glabra L. [Fabaceae, Glycyrrhizae radix et rhizoma]6g |
| Li 2018 (1) | Huangqi Guizhi Wuwu Decoction: Astragalus mongholicus Bunge [Fabaceae, Astragali radix] 45g, Neolitsea cassia (L.) Kosterm. [Lauraceae, Cinnamomi ramulus] 20g, Spatholobus suberectus Dunn [Fabaceae, Spatholobi caulis] 20g, Trichosanthes kirilowii Maxim. [Cucurbitaceae, Trichosanthis radix] 20g, Paeonia lactiflora Pall. [Paeoniaceae, Paeoniae radix alba] 20g, Carthamus tinctorius L. [Asteraceae, Carthami flos] 15g, Achyranthes bidentata Blume [Amaranthaceae, Achyranthis bidentatae radix] 15g, Boswellia frereana Birdw. [Burseraceae, Olibanum] 15g, Zingiber officinale Roscoe [Zingiberaceae, Zingiberis rhizoma recens] 15g, Glycyrrhiza glabra L. [Fabaceae, Glycyrrhizae radix et rhizoma] 8g, Ziziphus jujuba Mill. [Rhamnaceae, Jujubae fructus] 2 pieces |
| Zhao 2016 (3) | Huangqi Guizhi Wuwu Decoction: Astragalus mongholicus Bunge [Fabaceae, Astragali radix] 40g, Neolitsea cassia (L.) Kosterm. [Lauraceae, Cinnamomi ramulus] 15g, Spatholobus suberectus Dunn [Fabaceae, Spatholobi caulis] 15g, Trichosanthes kirilowii Maxim. [Cucurbitaceae, Trichosanthis radix] 15g, Paeonia lactiflora Pall. [Paeoniaceae, Paeoniae radix alba] 15g, Carthamus tinctorius L. [Asteraceae, Carthami flos] 10g, Achyranthes bidentata Blume [Amaranthaceae, Achyranthis bidentatae radix] 10g, Boswellia frereana Birdw. [Burseraceae, Olibanum] 10g, Zingiber officinale Roscoe [Zingiberaceae, Zingiberis rhizoma recens] 10g, Glycyrrhiza glabra L. [Fabaceae, Glycyrrhizae radix et rhizoma] 6g, Ziziphus jujuba Mill. [Rhamnaceae, Jujubae fructus] 3 pieces |
| Nie 2015 | Jianshenling capsule: Astragalus mongholicus Bunge [Fabaceae, Astragali radix] 30g, Alisma plantago-aquatica subsp. orientale (Sam.) Sam. [Alismataceae, Alismatis rhizoma] 10g, Epimedium sagittatum (Siebold & Zucc.) Maxim. [Berberidaceae, Epimedii folium] 15g, Poria cocos (Schw.)Wolf Poria [Polyporaceae, Poria] 15g, Plantago asiatica L. [Plantaginaceae, Plantaginis semen] 15g, Atractylodes macrocephala Koidz. [Asteraceae, Atractylodis macrocephalae rhizoma] 10g, Fossil fragments 15g, Ostrea gigas Thunberg [ostreidae, Ostreae concha] 30g, Leonurus japonicus Houtt. [Lamiaceae, Leonuri herba] 15g, Whitmania pigra Whitman [Hirudinidae, Hirudo] 10g, Coix lacryma-jobi var. ma-yuen (Rom.Caill.) Stapf [Poaceae, Coicis semen] 20g, Vigna umbellata (Thunb.) Ohwi & H.Ohashi [Fabaceae, Vignae semen] 20g, Panax quinquefolius L. [Araliaceae, Panacis quinquefolii radix] 15g |
| Jin 2019 | Jinchanhua Decoction: Paecilomyces cicadae (Miquel.) Samson 20g, Salvia miltiorrhiza Bunge [Lamiaceae, Salviae miltiorrhizae radix et rhizoma] 20g, Astragalus mongholicus Bunge [Fabaceae, Astragali radix] 30g, Atractylodes macrocephala Koidz. [Asteraceae, Atractylodis macrocephalae rhizoma] 15g, Poria cocos (Schw.) Wolf Poria [Polyporaceae, Poria] 15g, Rosa laevigata Michx. [Rosaceae, Rosae laevigatae fructus] 15g, Euryale ferox Salisb. [Nymphaeaceae, Euryales semen] 15g |
| Feng 2015 | Liuwei Dihuang decoction: Cornus officinalis Siebold & Zucc. [Cornaceae, Corni fructus] 12g, Schisandra chinensis (Turcz.) Baill. [Schisandraceae, Schisandrae chinensis fructus] 12g, Eclipta prostrata (L.) L. [Asteraceae, Ecliptae herba] 15g, Gardenia jasminoides J.Ellis [Rubiaceae, Gardeniae fructus] 9g, Poria cocos(Schw.)Wolf Poria [Polyporaceae, Poria] 15g, Dioscorea oppositifolia L. [Dioscoreaceae, Dioscoreae rhizoma] 15g, Atractylodes macrocephala Koidz. [Asteraceae, Atractylodis macrocephalae rhizoma] 12g, Ligustrum lucidum W.T.Aiton [Oleaceae, Ligustri lucidi fructus] 15g, Rehmannia glutinosa (Gaertn.) DC. [Orobanchaceae, Rehmanniae radix praeparata] 12g, Paeonia lactiflora Pall. [Paeoniaceae, Paeoniae radix alba] 12g, Angelica sinensis (Oliv.) Diels [Apiaceae, Angelicae sinensis radix] 12g |
| Chen 2015 | Pingtang Gushen Recipe: Astragalus mongholicus Bunge [Fabaceae, Astragali radix] 60g, Rehmannia glutinosa (Gaertn.) DC. [Orobanchaceae, Rehmanniae Radix] 30g, Scrophularia ningpoensis Hemsl. [Scrophulariaceae, Scrophulariae radix] 30g, Atractylodes lancea (Thunb.) DC. [Asteraceae, Atractylodis rhizoma] 15g, Pueraria montana var. lobata (Willd.) Maesen & S.M.Almeida ex Sanjappa & Predeep [Fabaceae, Puerariae lobatae radix] 30g, Monascus 30g, Salvia miltiorrhiza Bunge [Lamiaceae, Salviae miltiorrhizae radix et rhizoma] 10g, Euryale ferox Salisb. [Nymphaeaceae, Euryales semen] 10g, Whitmania pigra Whitman [Hirudinidae, Hirudo] 3g |
| Ou 2011 | Self-treating renal stasis Tongfu Decoction: Panax ginseng C.A.Mey. [Araliaceae, Ginseng radix et rhizoma] 15g, Salvia miltiorrhiza Bunge [Lamiaceae, Salviae miltiorrhizae radix et rhizoma] 30g, Astragalus mongholicus Bunge [Fabaceae, Astragali radix] 30g, Rheum palmatum L. [Polygonaceae, Rhei radix et rhizoma] 20g, Citrus × aurantium L. [Rutaceae, Aurantii fructus] 10g, Whitmania pigra Whitman [Hirudinidae, Hirudo] 10g, Buthus martensii Karsch [Buthidae, Scorpio] 10g, Cryptotympana pustulata Fabricius [Cicadidae, Cicadae periostracum] 10g, Poria cocos(Schw.)Wolf Poria [Polyporaceae, Poria] 15g |
| Li 2023 | Shenqi Buyi Prescription with Xiaoke Prescription: Astragalus mongholicus Bunge [Fabaceae, Astragali radix] 30g, Codonopsis pilosula (Franch.) Nannf. [Campanulaceae, Codonopsis radix] 20g, Rehmannia glutinosa (Gaertn.) DC. [Orobanchaceae, Rehmanniae radix praeparata] 15g, Ophiopogon japonicus (Thunb.) Ker Gawl. [Asparagaceae, Ophiopogonis radix] 15g, Scrophularia ningpoensis Hemsl. [Scrophulariaceae, Scrophulariae radix] 15g, Trichosanthes kirilowii Maxim. [Cucurbitaceae, Trichosanthis radix] 15g, Ligustrum lucidum W.T.Aiton [Oleaceae, Ligustri lucidi fructus] 15g, Dendrobium nobile Lindl. [Orchidaceae, Dendrobii caulis] 20g, Salvia miltiorrhiza Bunge [Lamiaceae, Salviae miltiorrhizae radix et rhizoma] 20g, Conioselinum anthriscoides 'Chuanxiong' [Apiaceae, Chuanxiong rhizoma] 20g, Paeonia lactiflora Pall. [Paeoniaceae, Paeoniae radix rubra] 20g, Paeonia lactiflora Pall. [Paeoniaceae, Paeoniae radix alba]20g, Leonurus japonicus Houtt. [Lamiaceae, Leonuri herba] 20g, Litchi chinensis Sonn. [Sapindaceae, Litchi semen] 30g, Zea mays L. [Poaceae, corn silk] 20g, Atractylodes lancea (Thunb.) DC. [Asteraceae, Atractylodis rhizoma] 10g, Coptis chinensis Franch. [Ranunculaceae, Coptidis rhizoma] 10g, Schisandra chinensis (Turcz.) Baill. [Schisandraceae, Schisandrae chinensis fructus] 5g |
| Hu 2018 (1) | Si Mo Yin Zi: Aquilaria sinensis (Lour.) Spreng. [Thymelaeaceae, Aquilariae lignum resinatum] 6g, Lindera aggregata (Sims) Kosterm. [Lauraceae, Linderae radix] 10g, Dolomiaea costus (Falc.) Kasana & A.K.Pandey [Asteraceae, Aucklandiae radix] 6g, Citrus × aurantium L. [Rutaceae, Aurantii fructus] 12g |
| Lin 2022 | Conventional Western Medicine Treatment: Astragalus mongholicus Bunge [Fabaceae, Astragali radix] 30g, Atractylodes macrocephala Koidz. [Asteraceae, Atractylodis macrocephalae rhizoma] 15g, Poria cocos(Schw.)Wolf Poria [Polyporaceae, Poria] 20g, Polygonatum sibiricum Redouté [Asparagaceae, Polygonati rhizoma] 15g, Rosa laevigata Michx. [Rosaceae, Rosae laevigatae fructus] 30g, Bombyx mori Linnaeus [Bombycidae, Bombyx batryticatus] 10g, Cornus officinalis Siebold & Zucc. [Cornaceae, Corni fructus] 15g, Coix lacryma-jobi var. ma-yuen (Rom.Caill.) Stapf [Poaceae, Coicis semen] 30g, Euryale ferox Salisb. [Nymphaeaceae, Euryales semen] 15g, Salvia miltiorrhiza Bunge [Lamiaceae, Salviae miltiorrhizae radix et rhizoma] 30g, Conioselinum anthriscoides 'Chuanxiong' [Apiaceae, Chuanxiong rhizoma] 10g, Gallus gallus domesticus Brisson [Phasianidae, Galli gigerii endothelium corneum] 10g, Litchi chinensis Sonn. [Sapindaceae, Litchi semen] 10g, Glycyrrhiza uralensis Fisch. ex DC. [Fabaceae, Glycyrrhizae radix et rhizoma praeparata cum melle] 3g |
| Yang 2021 (1) | Wenyang Jiangzhuo Tongluo Recipe: Astragalus mongholicus Bunge [Fabaceae, Astragali radix] 30-60g, Codonopsis pilosula (Franch.) Nannf. [Campanulaceae, Codonopsis radix] 20g, Atractylodes macrocephala Koidz. [Asteraceae, Atractylodis macrocephalae rhizoma] 15g, Paeonia lactiflora Pall. [Paeoniaceae, Paeoniae radix alba] 18g, Coptis chinensis Franch. [Ranunculaceae, Coptidis rhizoma] 6-18g, Plantago asiatica L. [Plantaginaceae, Plantaginis semen] 30g, Aconitum carmichaelii Debeaux [Ranunculaceae, Aconiti lateralis radix praeparata] 10-15g, Epimedium sagittatum (Siebold & Zucc.) Maxim. [Berberidaceae, Epimedii folium] 20g, Dioscorea oppositifolia L. [Dioscoreaceae, Dioscoreae rhizoma] 30g, Whitmania pigra Whitman [Hirudinidae, Hirudo] 3-6g, Salvia miltiorrhiza Bunge [Lamiaceae, Salviae miltiorrhizae radix et rhizoma] 30g, Glycyrrhiza uralensis Fisch. ex DC. [Fabaceae, Glycyrrhizae radix et rhizoma praeparata cum melle] 6g |
| Li 2019 | Yigi Yangyin Decoction: Pseudostellaria heterophylla (Miq.) Pax [Caryophyllaceae, Pseudostellariae radix] 20g, Astragalus mongholicus Bunge [Fabaceae, Astragali radix] 20g, Rehmannia glutinosa (Gaertn.) DC. [Orobanchaceae, Rehmanniae Radix] 15g, Ophiopogon japonicus (Thunb.) Ker Gawl. [Asparagaceae, Ophiopogonis radix] 15g, Pueraria montana var. lobata (Willd.) Maesen & S.M.Almeida ex Sanjappa & Predeep [Fabaceae, Puerariae lobatae radix] 15g, Rehmannia glutinosa (Gaertn.) DC. [Orobanchaceae, Rehmanniae radix praeparata] 15g, Cornus officinalis Siebold & Zucc. [Cornaceae, Corni fructus] 10g, Dioscorea oppositifolia L. [Dioscoreaceae, Dioscoreae rhizoma]10g, Coptis chinensis Franch. [Ranunculaceae, Coptidis rhizoma] 10g, Glycyrrhiza glabra L. [Fabaceae, Glycyrrhizae radix et rhizoma] 8g |
| Su 2019 | Yiqi Guben Decoction: Astragalus mongholicus Bunge [Fabaceae, Astragali radix] 30g, Atractylodes macrocephala Koidz. [Asteraceae, Atractylodis macrocephalae rhizoma] 15g, Poria cocos(Schw.)Wolf Poria [Polyporaceae, Poria] 20g, Polygonatum sibiricum Redouté [Asparagaceae, Polygonati rhizoma] 15g, Rosa laevigata Michx. [Rosaceae, Rosae laevigatae fructus]30g, Bombyx mori Linnaeus [Bombycidae, Bombyx batryticatus] 10g, Cornus officinalis Siebold & Zucc. [Cornaceae, Corni fructus] 15g, Coix lacryma-jobi var. ma-yuen (Rom.Caill.) Stapf [Poaceae, Coicis semen] 30g, Euryale ferox Salisb. [Nymphaeaceae, Euryales semen] 15g, Salvia miltiorrhiza Bunge [Lamiaceae, Salviae miltiorrhizae radix et rhizoma] 30g, Conioselinum anthriscoides 'Chuanxiong' [Apiaceae, Chuanxiong rhizoma] 10g, Gallus gallus domesticus Brisson [Phasianidae, Galli gigerii endothelium corneum] 10g, Litchi chinensis Sonn. [Sapindaceae, Litchi semen] 10g, Glycyrrhiza uralensis Fisch. ex DC. [Fabaceae, Glycyrrhizae radix et rhizoma praeparata cum melle] 3g |
| Wang 2022 (2) | Yiqi Guben Decoction: Astragalus mongholicus Bunge [Fabaceae, Astragali radix] 30g, Atractylodes macrocephala Koidz. [Asteraceae, Atractylodis macrocephalae rhizoma] 15g, Poria cocos(Schw.)Wolf Poria [Polyporaceae, Poria] 20g, Polygonatum sibiricum Redouté [Asparagaceae, Polygonati rhizoma] 15g, Rosa laevigata Michx. [Rosaceae, Rosae laevigatae fructus] 30g, Bombyx mori Linnaeus [Bombycidae, Bombyx batryticatus] 10g, Cornus officinalis Siebold & Zucc. [Cornaceae, Corni fructus] 15g, Coix lacryma-jobi var. ma-yuen (Rom.Caill.) Stapf [Poaceae, Coicis semen] 30g, Euryale ferox Salisb. [Nymphaeaceae, Euryales semen] 15g, Salvia miltiorrhiza Bunge [Lamiaceae, Salviae miltiorrhizae radix et rhizoma] 30g, Conioselinum anthriscoides 'Chuanxiong' [Apiaceae, Chuanxiong rhizoma] 10g, Gallus gallus domesticus Brisson [Phasianidae, Galli gigerii endothelium corneum] 10g, Litchi chinensis Sonn. [Sapindaceae, Litchi semen] 10g, Glycyrrhiza uralensis Fisch. ex DC. [Fabaceae, Glycyrrhizae radix et rhizoma praeparata cum melle] 3g |
| Jiang 2019 | Yiqi Yangyin Decoction: Pseudostellaria heterophylla (Miq.) Pax [Caryophyllaceae, Pseudostellariae radix] 20g, Astragalus mongholicus Bunge [Fabaceae, Astragali radix] 20g, Angelica sinensis (Oliv.) Diels [Apiaceae, Angelicae sinensis radix] 15g, Cornus officinalis Siebold & Zucc. [Cornaceae, Corni fructus] 15g, Rehmannia glutinosa (Gaertn.) DC. [Orobanchaceae, Rehmanniae Radix] 15g, Rehmannia glutinosa (Gaertn.) DC. [Orobanchaceae, Rehmanniae radix praeparata] 15g, Dioscorea oppositifolia L. [Dioscoreaceae, Dioscoreae rhizoma] 15g, Ophiopogon japonicus (Thunb.) Ker Gawl. [Asparagaceae, Ophiopogonis radix] 15g, Alisma plantago-aquatica subsp. orientale (Sam.) Sam. [Alismataceae, Alismatis rhizoma] 15g |
| Feng 2023 | Yishen Paidu Formula: Astragalus mongholicus Bunge [Fabaceae, Astragali radix] 20g, Codonopsis pilosula (Franch.) Nannf. [Campanulaceae, Codonopsis radix] 10g, Atractylodes macrocephala Koidz. [Asteraceae, Atractylodis macrocephalae rhizoma] 10g, Dioscorea oppositifolia L. [Dioscoreaceae, Dioscoreae rhizoma] 15g, Cornus officinalis Siebold & Zucc. [Cornaceae, Corni fructus] 12g, Conioselinum anthriscoides 'Chuanxiong' [Apiaceae, Chuanxiong rhizoma] 12g, Poria cocos(Schw.)Wolf Poria [Polyporaceae, Poria] 15g, Paeonia × suffruticosa Andrews [Paeoniaceae, Moutan cortex] 10g, Cuscuta chinensis Lam. [Convolvulaceae, Cuscutae semen] 10g, Salvia miltiorrhiza Bunge [Lamiaceae, Salviae miltiorrhizae radix et rhizoma] 15g, Achyranthes bidentata Blume [Amaranthaceae, Achyranthis bidentatae radix] 15g, Rehmannia glutinosa (Gaertn.) DC. [Orobanchaceae, Rehmanniae radix praeparata] 15g, Euonymus alatus (Thunb.) Siebold [Celastraceae, Euonymus alatus] 15g, Epimedium sagittatum (Siebold & Zucc.) Maxim. [Berberidaceae, Epimedii folium] 15g |
| Gao 2010 | Yishen Tangshi'an Recipe: Astragalus mongholicus Bunge [Fabaceae, Astragali radix] 30g, Cornus officinalis Siebold & Zucc. [Cornaceae, Corni fructus] 15g, Pueraria montana var. lobata (Willd.) Maesen & S.M.Almeida ex Sanjappa & Predeep [Fabaceae, Puerariae lobatae radix] 15g, Alisma plantago-aquatica subsp. orientale (Sam.) Sam. [Alismataceae, Alismatis rhizoma] 12g, Salvia miltiorrhiza Bunge [Lamiaceae, Salviae miltiorrhizae radix et rhizoma] 30g, Angelica sinensis (Oliv.) Diels [Apiaceae, Angelicae sinensis radix] 12g, Dioscorea oppositifolia L. [Dioscoreaceae, Dioscoreae rhizoma] 20g |
| Wen 2006 | Zhenqing Prescription: Ligustrum lucidum W.T.Aiton [Oleaceae, Ligustri lucidi fructus], Dioscorea oppositifolia L. [Dioscoreaceae, Dioscoreae rhizoma], Pheretima aspergillum (E.Perrier) [Megascolecidae, Pheretima] |
| Zhang 2022 | Zhenwu Decoction: Panax quinquefolius L. [Araliaceae, Panacis quinquefolii radix] 30g, Dioscorea oppositifolia L. [Dioscoreaceae, Dioscoreae rhizoma] 20g, Atractylodes macrocephala Koidz. [Asteraceae, Atractylodis macrocephalae rhizoma] 20g, Poria cocos(Schw.)Wolf Poria [Polyporaceae, Poria] 15g, Paeonia lactiflora Pall. [Paeoniaceae, Paeoniae radix alba] 15g, Aconitum carmichaelii Debeaux [Ranunculaceae, Aconiti lateralis radix praeparata] 12g, Alisma plantago-aquatica subsp. orientale (Sam.) Sam. [Alismataceae, Alismatis rhizoma] 10g, Carthamus tinctorius L. [Asteraceae, Carthami flos] 10g, Prunus persica (L.) Batsch [Rosaceae, Persicae semen] 10g |
| Zhang 2021 | Zicui Yishen Decoction: Astragalus mongholicus Bunge [Fabaceae, Astragali radix] 30g, Euryale ferox Salisb. [Nymphaeaceae, Euryales semen] 30g, Smilax glabra Roxb. [Smilacaceae, Smilacis glabrae rhizoma] 30g, Pueraria montana var. lobata (Willd.) Maesen & S.M.Almeida ex Sanjappa & Predeep [Fabaceae, Puerariae lobatae radix] 20g, Rehmannia glutinosa (Gaertn.) DC. [Orobanchaceae, Rehmanniae Radix] 15g, Cornus officinalis Siebold & Zucc. [Cornaceae, Corni fructus] 15g, Dioscorea oppositifolia L. [Dioscoreaceae, Dioscoreae rhizoma] 15g, Scrophularia ningpoensis Hemsl. [Scrophulariaceae, Scrophulariae radix] 15g, Atractylodes lancea (Thunb.) DC. [Asteraceae, Atractylodis rhizoma] 15g, Alisma plantago-aquatica subsp. orientale (Sam.) Sam. [Alismataceae, Alismatis rhizoma] 15g, Carthamus tinctorius L. [Asteraceae, Carthami flos] 6g |
| Traditional Chinese patent medicines | |
| Fang 2019 | Bailing Capsules: Cordyceps sinensis（BerK.）Sacc. [Clavicipitaceae, Cordyceps] 0.2g or 0.5g/capsule |
| Yu 2021 | Bailing Capsules: Cordyceps sinensis（BerK.）Sacc. [Clavicipitaceae, Cordyceps] 0.2g or 0.5g/capsule |
| Wang 2018 (1) | Shenyan Kangfu Tablets: Panax quinquefolius L. [Araliaceae, Panacis quinquefolii radix] 17.4g, Panax ginseng C.A.Mey. [Araliaceae, Ginseng radix et rhizoma] 5.8g, Rehmannia glutinosa (Gaertn.) DC. [Orobanchaceae, Rehmanniae Radix] 58.1g, Eucommia ulmoides Oliv. [Eucommiaceae, Eucommiae cortex] 34.9g, Dioscorea oppositifolia L. [Dioscoreaceae, Dioscoreae rhizoma] 58.1g, Scleromitrion diffusum (Willd.) R. J. Wang [Rubiaceae, Hedyotis diffusa] 29.1g, Glycine max (L.) Merr. [Fabaceae, Sojae semen nigrum] 58.1g, Smilax glabra Roxb. [Smilacaceae, Smilacis glabrae rhizoma] 58.1g, Leonurus japonicus Houtt. [Lamiaceae, Leonuri herba] 58.1g, Salvia miltiorrhiza Bunge [Lamiaceae, Salviae miltiorrhizae radix et rhizoma] 29.1g, Alisma plantago-aquatica subsp. orientale (Sam.) Sam. [Alismataceae, Alismatis rhizoma] 29.1g, Imperata cylindrica (L.) Raeusch. [Poaceae, Imperatae rhizoma] 87.2g, Platycodon grandiflorus (Jacq.) A.DC. [Campanulaceae, Platycodonis radix] 58.1g |
| Shen 2013 | Bailing Capsules: Cordyceps sinensis（BerK.）Sacc. [Clavicipitaceae, Cordyceps] 0.2g or 0.5g/capsule |
| Wang 2016 | Bailing Capsules: Cordyceps sinensis（BerK.）Sacc. [Clavicipitaceae, Cordyceps] 0.2g or 0.5g/capsule |
| Xu 2022 | Bailing Capsules: Cordyceps sinensis（BerK.）Sacc. [Clavicipitaceae, Cordyceps] 0.2g or 0.5g/capsule |
| Peng 2013 | Bailing Capsules: Cordyceps sinensis（BerK.）Sacc. [Clavicipitaceae, Cordyceps] 0.2g or 0.5g/capsule |
| Yang 2016 | Bailing Capsules: Cordyceps sinensis（BerK.）Sacc. [Clavicipitaceae, Cordyceps] 0.2g or 0.5g/capsule |
| Sun 2012 (1) | Bailing Capsules: Cordyceps sinensis（BerK.）Sacc. [Clavicipitaceae, Cordyceps] 0.2g or 0.5g/capsule |
| Hong 2010 | Bailing Capsules: Cordyceps sinensis（BerK.）Sacc. [Clavicipitaceae, Cordyceps] 0.2g or 0.5g/capsule |
| Wang 2007 | Bailing Capsules: Cordyceps sinensis（BerK.）Sacc. [Clavicipitaceae, Cordyceps] 0.2g or 0.5g/capsule |
| Shen 2021 | Bailing Tablets: Cordyceps sinensis（BerK.）Sacc. [Clavicipitaceae, Cordyceps] |
| Wang 2022 (3) | Compound Danshen Dripping Pills: Salvia miltiorrhiza Bunge [Lamiaceae, Salviae miltiorrhizae radix et rhizoma] 90g, Panax notoginseng (Burkill) F.H.Chen [Araliaceae, Notoginseng radix et rhizoma] 17.6g, Borneolum syntheticum 1g |
| BAI 2008 | Compound Danshen Dripping Pills: Salvia miltiorrhiza Bunge [Lamiaceae, Salviae miltiorrhizae radix et rhizoma] 90g, Panax notoginseng (Burkill) F.H.Chen [Araliaceae, Notoginseng radix et rhizoma] 17.6g, Borneolum syntheticum 1g |
| Wang 2018 (2) | Compound Danshen Dripping Pills: Salvia miltiorrhiza Bunge [Lamiaceae, Salviae miltiorrhizae radix et rhizoma] 90g, Panax notoginseng (Burkill) F.H.Chen [Araliaceae, Notoginseng radix et rhizoma] 17.6g, Borneolum syntheticum 1g |
| Xie 2016 | Compound Danshen Dripping Pills: Salvia miltiorrhiza Bunge [Lamiaceae, Salviae miltiorrhizae radix et rhizoma] 90g, Panax notoginseng (Burkill) F.H.Chen [Araliaceae, Notoginseng radix et rhizoma] 17.6g, Borneolum syntheticum 1g |
| Zhu 2014 | Compound Danshen Dripping Pills: Salvia miltiorrhiza Bunge [Lamiaceae, Salviae miltiorrhizae radix et rhizoma] 90g, Panax notoginseng (Burkill) F.H.Chen [Araliaceae, Notoginseng radix et rhizoma] 17.6g, Borneolum syntheticum 1g |
| Ma 2017 (2) | Compound Danshen Dripping Pills: Salvia miltiorrhiza Bunge [Lamiaceae, Salviae miltiorrhizae radix et rhizoma] 90g, Panax notoginseng (Burkill) F.H.Chen [Araliaceae, Notoginseng radix et rhizoma] 17.6g, Borneolum syntheticum 1g |
| Lin 2019 | Congrong Yishen Granules: Schisandra chinensis (Turcz.) Baill. [Schisandraceae, Schisandrae chinensis fructus] 360g, Cistanche deserticola Ma [Orobanchaceae, Cistanches herba] 360g, Poria cocos(Schw.)Wolf Poria [Polyporaceae, Poria] 180g, Cuscuta chinensis Lam. [Convolvulaceae, Cuscutae semen] 360g, Plantago asiatica L. [Plantaginaceae, Plantaginis semen] 450g, Morindae officinalis radix [Rubiaceae, Morindae officinalis radix] 540g |
| Su 2020 (2) | Jinlida Granules: Panax ginseng C.A.Mey. [Araliaceae, Ginseng radix et rhizoma] 184.5g, Polygonatum sibiricum Redouté [Asparagaceae, Polygonati rhizoma] 244.5g, Atractylodes lancea (Thunb.) DC. [Asteraceae, Atractylodis rhizoma] 122.2g, Sophora flavescens Aiton [Fabaceae, Sophorae flavescentis radix] 100g, Ophiopogon japonicus (Thunb.) Ker Gawl. [Asparagaceae, Ophiopogonis radix] 244.5g, Rehmannia glutinosa (Gaertn.) DC. [Orobanchaceae, Rehmanniae Radix] 184.5g, Reynoutria multiflora (Thunb.) Moldenke [Polygonaceae, Polygoni multiflori radix] 149g, Cornus officinalis Siebold & Zucc. [Cornaceae, Corni fructus] 244.5g, Poria cocos(Schw.)Wolf Poria [Polyporaceae, Poria] 149g, Eupatorium fortunei Turcz. [Asteraceae, Eupatorii herba] 100g, Coptis chinensis Franch. [Ranunculaceae, Coptidis rhizoma] 100g, Anemarrhena asphodeloides Bunge [Asparagaceae, Anemarrhenae rhizoma] 122.2g, Epimedium sagittatum (Siebold & Zucc.) Maxim. [Berberidaceae, Epimedii folium] 100g, Salvia miltiorrhiza Bunge [Lamiaceae, Salviae miltiorrhizae radix et rhizoma] 160g, Pueraria montana var. lobata (Willd.) Maesen & S.M.Almeida ex Sanjappa & Predeep [Fabaceae, Puerariae lobatae radix] 244.5g, Litchi chinensis Sonn. [Sapindaceae, Litchi semen] 244.5g, Lycium barbarum L. [Solanaceae, Lycii cortex] 149g |
| Huang 2010 | Jinshuibao Capsules: Cordyceps sinensis（BerK.）Sacc. [Clavicipitaceae, Cordyceps] (Cs-4) 0.33g/capsule |
| Pan 2016 | Jinshuibao Capsules: Cordyceps sinensis（BerK.）Sacc. [Clavicipitaceae, Cordyceps] (Cs-4) 0.33g/capsule |
| Huang 2014 | Jinshuibao Capsules: Cordyceps sinensis（BerK.）Sacc. [Clavicipitaceae, Cordyceps]（Cs-4）0.33g/capsule; Compound Danshen Dripping Pills: Salvia miltiorrhiza Bunge [Lamiaceae, Salviae miltiorrhizae radix et rhizoma] 90g, Panax notoginseng (Burkill) F.H.Chen [Araliaceae, Notoginseng radix et rhizoma] 17.6g, Borneolum syntheticum 1g |
| Yi 2009 | Jinshuibao Capsules: Cordyceps sinensis（BerK.）Sacc. [Clavicipitaceae, Cordyceps] (Cs-4) 0.33g/capsule |
| Zhong 2020 | Jinshuibao Tablets: Cordyceps sinensis（BerK.）Sacc. [Clavicipitaceae, Cordyceps] (Cs-4) 0.2g/tablet |
| Zhang 2014 | Niaoduqing granules: Rheum palmatum L. [Polygonaceae, Rhei radix et rhizoma], Astragalus mongholicus Bunge [Fabaceae, Astragali radix], Morus alba L. [Moraceae, Mori cortex], Codonopsis pilosula (Franch.) Nannf. [Campanulaceae, Codonopsis radix], Atractylodes macrocephala Koidz. [Asteraceae, Atractylodis macrocephalae rhizoma], Poria cocos(Schw.)Wolf Poria [Polyporaceae, Poria], Reynoutria multiflora (Thunb.) Moldenke [Polygonaceae, Polygoni multiflori radix], Paeonia lactiflora Pall. [Paeoniaceae, Paeoniae radix alba], Salvia miltiorrhiza Bunge [Lamiaceae, Salviae miltiorrhizae radix et rhizoma], Plantago asiatica L. [Plantaginaceae, Plantaginis herba] |
| Sun 2012 (2) | Huangkui Capsules: Abelmoschus manihot (L.) Medik. [Malvaceae, Abelmoschi corolla] |
| Li 2016 (1) | Huangkui Capsules: Abelmoschus manihot (L.) Medik. [Malvaceae, Abelmoschi corolla] |
| WANG 2023 | Qi-Kui Granules: Astragalus mongholicus Bunge [Fabaceae, Astragali radix], Reynoutria multiflora (Thunb.) Moldenke [Polygonaceae, Polygoni multiflori radix], Abelmoschus manihot (L.) Medik. [Malvaceae, Abelmoschi corolla] |
| Shi 2019 | Shen'an Capsules: BoenninghauseniasessilicarpaLevl., Orthosiphon aristatus (Blume) Miq. [Lamiaceae, java tea], Phellodendron chinense C.K.Schneid. [Rutaceae, Phellodendri chinensis cortex], Imperata cylindrica (L.) Raeusch. [Poaceae, Imperatae rhizoma], Poria cocos(Schw.)Wolf Poria [Polyporaceae, Poria], Atractylodes macrocephala Koidz. [Asteraceae, Atractylodis macrocephalae rhizoma], Lonicera japonica Thunb. [Caprifoliaceae, Lonicerae japonicae flos], Astragalus mongholicus Bunge [Fabaceae, Astragali radix], Alisma plantago-aquatica subsp. orientale (Sam.) Sam. [Alismataceae, Alismatis rhizoma], Lophatherum gracile Brongn. [Poaceae, Lophatheri herba], Juncus effusus L. [Juncaceae, Junci medulla], Glycyrrhiza glabra L. [Fabaceae, Glycyrrhizae radix et rhizoma] |
| Shu 2010 | Shenyan Kangfu Tablets: Panax quinquefolius L. [Araliaceae, Panacis quinquefolii radix] 17.4g, Panax ginseng C.A.Mey. [Araliaceae, Ginseng radix et rhizoma] 5.8g, Rehmannia glutinosa (Gaertn.) DC. [Orobanchaceae, Rehmanniae Radix] 58.1g, Eucommia ulmoides Oliv. [Eucommiaceae, Eucommiae cortex] 34.9g, Dioscorea oppositifolia L. [Dioscoreaceae, Dioscoreae rhizoma] 58.1g, Scleromitrion diffusum (Willd.) R. J. Wang [Rubiaceae, Hedyotis diffusa] 29.1g, Glycine max (L.) Merr. [Fabaceae, Sojae semen nigrum] 58.1g, Smilax glabra Roxb. [Smilacaceae, Smilacis glabrae rhizoma] 58.1g, Leonurus japonicus Houtt. [Lamiaceae, Leonuri herba] 58.1g, Salvia miltiorrhiza Bunge [Lamiaceae, Salviae miltiorrhizae radix et rhizoma] 29.1g, Alisma plantago-aquatica subsp. orientale (Sam.) Sam. [Alismataceae, Alismatis rhizoma] 29.1g, Imperata cylindrica (L.) Raeusch. [Poaceae, Imperatae rhizoma] 87.2g, Platycodon grandiflorus (Jacq.) A.DC. [Campanulaceae, Platycodonis radix] 58.1g |
| Guo 2021 | Shenyan Kangfu Tablets: Panax quinquefolius L. [Araliaceae, Panacis quinquefolii radix] 17.4g, Panax ginseng C.A.Mey. [Araliaceae, Ginseng radix et rhizoma] 5.8g, Rehmannia glutinosa (Gaertn.) DC. [Orobanchaceae, Rehmanniae Radix] 58.1g, Eucommia ulmoides Oliv. [Eucommiaceae, Eucommiae cortex] 34.9g, Dioscorea oppositifolia L. [Dioscoreaceae, Dioscoreae rhizoma] 58.1g, Scleromitrion diffusum (Willd.) R. J. Wang [Rubiaceae, Hedyotis diffusa] 29.1g, Glycine max (L.) Merr. [Fabaceae, Sojae semen nigrum] 58.1g, Smilax glabra Roxb. [Smilacaceae, Smilacis glabrae rhizoma] 58.1g, Leonurus japonicus Houtt. [Lamiaceae, Leonuri herba] 58.1g, Salvia miltiorrhiza Bunge [Lamiaceae, Salviae miltiorrhizae radix et rhizoma] 29.1g, Alisma plantago-aquatica subsp. orientale (Sam.) Sam. [Alismataceae, Alismatis rhizoma] 29.1g, Imperata cylindrica (L.) Raeusch. [Poaceae, Imperatae rhizoma] 87.2g, Platycodon grandiflorus (Jacq.) A.DC. [Campanulaceae, Platycodonis radix] 58.1g |
| Li 2020 | Shenyan Kangfu Tablets: Panax quinquefolius L. [Araliaceae, Panacis quinquefolii radix] 17.4g, Panax ginseng C.A.Mey. [Araliaceae, Ginseng radix et rhizoma] 5.8g, Rehmannia glutinosa (Gaertn.) DC. [Orobanchaceae, Rehmanniae Radix] 58.1g, Eucommia ulmoides Oliv. [Eucommiaceae, Eucommiae cortex] 34.9g, Dioscorea oppositifolia L. [Dioscoreaceae, Dioscoreae rhizoma] 58.1g, Scleromitrion diffusum (Willd.) R. J. Wang [Rubiaceae, Hedyotis diffusa] 29.1g, Glycine max (L.) Merr. [Fabaceae, Sojae semen nigrum] 58.1g, Smilax glabra Roxb. [Smilacaceae, Smilacis glabrae rhizoma] 58.1g, Leonurus japonicus Houtt. [Lamiaceae, Leonuri herba] 58.1g, Salvia miltiorrhiza Bunge [Lamiaceae, Salviae miltiorrhizae radix et rhizoma] 29.1g, Alisma plantago-aquatica subsp. orientale (Sam.) Sam. [Alismataceae, Alismatis rhizoma] 29.1g, Imperata cylindrica (L.) Raeusch. [Poaceae, Imperatae rhizoma] 87.2g, Platycodon grandiflorus (Jacq.) A.DC. [Campanulaceae, Platycodonis radix] 58.1g |
| Liu 2023 | Yishen Huashi Granules: Panax ginseng C.A.Mey. [Araliaceae, Ginseng radix et rhizoma] 170.5g, Astragalus mongholicus Bunge [Fabaceae, Astragali radix] 341g, Atractylodes macrocephala Koidz. [Asteraceae, Atractylodis macrocephalae rhizoma] 51.1g, Poria cocos(Schw.)Wolf Poria [Polyporaceae, Poria] 51.1g, Alisma plantago-aquatica subsp. orientale (Sam.) Sam. [Alismataceae, Alismatis rhizoma] 51.1g, Pinellia ternata (Thunb.) Makino [Araceae, Pinelliae rhizoma] 170.5g, Hansenia weberbaueriana (Fedde ex H.Wolff) Pimenov & Kljuykov [Apiaceae, Notopterygii rhizoma et radix] 85.2g, Angelica biserrata (R.H.Shan & C.Q.Yuan) C.Q.Yuan & R.H.Shan [Apiaceae, Angelicae pubescentis radix] 85.2g, Saposhnikovia divaricata (Turcz. ex Ledeb.) Schischk. [Apiaceae, Saposhnikoviae radix] 85.2g, Bupleurum chinense DC. [Apiaceae, BUPLEURI RADIX] 51.1g, Coptis chinensis Franch. [Ranunculaceae, Coptidis rhizoma] 34.1g, Paeonia lactiflora Pall. [Paeoniaceae, Paeoniae radix alba] 85.2g, Citrus reticulata Blanco [Rutaceae, Citri reticulatae pericarpium] 68.2g, Glycyrrhiza uralensis Fisch. ex DC. [Fabaceae, Glycyrrhizae radix et rhizoma praeparata cum melle] 170.5g, Zingiber officinale Roscoe [Zingiberaceae, Zingiberis rhizoma recens] 50g, Ziziphus jujuba Mill. [Rhamnaceae, Jujubae fructus] 100g |
| HU 2018 (2) | Yishen Huashi Granules: Panax ginseng C.A.Mey. [Araliaceae, Ginseng radix et rhizoma] 170.5g, Astragalus mongholicus Bunge [Fabaceae, Astragali radix] 341g, Atractylodes macrocephala Koidz. [Asteraceae, Atractylodis macrocephalae rhizoma] 51.1g, Poria cocos (Schw.)Wolf Poria [Polyporaceae, Poria] 51.1g, Alisma plantago-aquatica subsp. orientale (Sam.) Sam. [Alismataceae, Alismatis rhizoma] 51.1g, Pinellia ternata (Thunb.) Makino [Araceae, Pinelliae rhizoma] 170.5g, Hansenia weberbaueriana (Fedde ex H.Wolff) Pimenov & Kljuykov [Apiaceae, Notopterygii rhizoma et radix] 85.2g, Angelica biserrata (R.H.Shan & C.Q.Yuan) C.Q.Yuan & R.H.Shan [Apiaceae, Angelicae pubescentis radix] 85.2g, Saposhnikovia divaricata (Turcz. ex Ledeb.) Schischk. [Apiaceae, Saposhnikoviae radix] 85.2g, Bupleurum chinense DC. [Apiaceae, BUPLEURI RADIX] 51.1g, Coptis chinensis Franch. [Ranunculaceae, Coptidis rhizoma] 34.1g, Paeonia lactiflora Pall. [Paeoniaceae, Paeoniae radix alba] 85.2g, Citrus reticulata Blanco [Rutaceae, Citri reticulatae pericarpium] 68.2g, Glycyrrhiza uralensis Fisch. ex DC. [Fabaceae, Glycyrrhizae radix et rhizoma praeparata cum melle] 170.5g, Zingiber officinale Roscoe [Zingiberaceae, Zingiberis rhizoma recens] 50g, Ziziphus jujuba Mill. [Rhamnaceae, Jujubae fructus] 100g |
| Chen 2018 | Yishen Huashi Granules: Panax ginseng C.A.Mey. [Araliaceae, Ginseng radix et rhizoma] 170.5g, Astragalus mongholicus Bunge [Fabaceae, Astragali radix] 341g, Atractylodes macrocephala Koidz. [Asteraceae, Atractylodis macrocephalae rhizoma] 51.1g, Poria cocos (Schw.) Wolf Poria [Polyporaceae, Poria] 51.1g, Alisma plantago-aquatica subsp. orientale (Sam.) Sam. [Alismataceae, Alismatis rhizoma] 51.1g, Pinellia ternata (Thunb.) Makino [Araceae, Pinelliae rhizoma] 170.5g, Hansenia weberbaueriana (Fedde ex H.Wolff) Pimenov & Kljuykov [Apiaceae, Notopterygii rhizoma et radix] 85.2g, Angelica biserrata (R.H.Shan & C.Q.Yuan) C.Q.Yuan & R.H.Shan [Apiaceae, Angelicae pubescentis radix] 85.2g, Saposhnikovia divaricata (Turcz. ex Ledeb.) Schischk. [Apiaceae, Saposhnikoviae radix] 85.2g, Bupleurum chinense DC. [Apiaceae, BUPLEURI RADIX] 51.1g, Coptis chinensis Franch. [Ranunculaceae, Coptidis rhizoma] 34.1g, Paeonia lactiflora Pall. [Paeoniaceae, Paeoniae radix alba] 85.2g, Citrus reticulata Blanco [Rutaceae, Citri reticulatae pericarpium] 68.2g, Glycyrrhiza uralensis Fisch. ex DC. [Fabaceae, Glycyrrhizae radix et rhizoma praeparata cum melle] 170.5g, Zingiber officinale Roscoe [Zingiberaceae, Zingiberis rhizoma recens] 50g, Ziziphus jujuba Mill. [Rhamnaceae, Jujubae fructus] 100g |
| Hu 2016 | Yishen Huashi Granules: Panax ginseng C.A.Mey. [Araliaceae, Ginseng radix et rhizoma] 170.5g, Astragalus mongholicus Bunge [Fabaceae, Astragali radix] 341g, Atractylodes macrocephala Koidz. [Asteraceae, Atractylodis macrocephalae rhizoma] 51.1g, Poria cocos (Schw.) Wolf Poria [Polyporaceae, Poria] 51.1g, Alisma plantago-aquatica subsp. orientale (Sam.) Sam. [Alismataceae, Alismatis rhizoma] 51.1g, Pinellia ternata (Thunb.) Makino [Araceae, Pinelliae rhizoma] 170.5g, Hansenia weberbaueriana (Fedde ex H.Wolff) Pimenov & Kljuykov [Apiaceae, Notopterygii rhizoma et radix] 85.2g, Angelica biserrata (R.H.Shan & C.Q.Yuan) C.Q.Yuan & R.H.Shan [Apiaceae, Angelicae pubescentis radix] 85.2g, Saposhnikovia divaricata (Turcz. ex Ledeb.) Schischk. [Apiaceae, Saposhnikoviae radix] 85.2g, Bupleurum chinense DC. [Apiaceae, BUPLEURI RADIX] 51.1g, Coptis chinensis Franch. [Ranunculaceae, Coptidis rhizoma] 34.1g, Paeonia lactiflora Pall. [Paeoniaceae, Paeoniae radix alba] 85.2g, Citrus reticulata Blanco [Rutaceae, Citri reticulatae pericarpium] 68.2g, Glycyrrhiza uralensis Fisch. ex DC. [Fabaceae, Glycyrrhizae radix et rhizoma praeparata cum melle] 170.5g, Zingiber officinale Roscoe [Zingiberaceae, Zingiberis rhizoma recens] 50g, Ziziphus jujuba Mill. [Rhamnaceae, Jujubae fructus] 100g |
| Traditional Chinese Medicine Extracts | |
| Chen 2022 | Haikunshenxi Capsules: Fucoidan |
