# Supplementary material for: Mechanism of traditional Chinese medicine in elderly diabetes mellitus and a systematic review of its clinical application
Source: Front Pharmacol. 2024 Mar 6;15:1339148. doi: 10.3389/fphar.2024.1339148 (PMC10953506; doi:10.3389/fphar.2024.1339148)
Supplement: Supplementary file 2 [file DataSheet1.zip › Supplementary Table S1-17/Supplementary Table S6a.docx]

Supplementary Table S6a | Frequency of Traditional Chinese Medicine for the treatment of elderly DKD in Traditional Chinese Prescription.

| Traditional Chinese Medicine | Frequency |
| --- | --- |
| Astragalus mongholicus Bunge [Fabaceae, Astragali radix] | 17 |
| Dioscorea oppositifolia L. [Dioscoreaceae, Dioscoreae rhizoma] | 10 |
| Poria cocos (Schw.) Wolf Poria [Polyporaceae, Poria] | 10 |
| Atractylodes macrocephala Koidz. [Asteraceae, Atractylodis macrocephalae rhizoma] | 9 |
| Cornus officinalis Siebold & Zucc. [Cornaceae, Corni fructus] | 9 |
| Salvia miltiorrhiza Bunge [Lamiaceae, Salviae miltiorrhizae radix et rhizoma] | 9 |
| Alisma plantago-aquatica subsp. orientale (Sam.) Sam. [Alismataceae, Alismatis rhizoma] | 6 |
| Euryale ferox Salisb. [Nymphaeaceae, Euryales semen] | 6 |
| Paeonia lactiflora Pall. [Paeoniaceae, Paeoniae radix alba] | 6 |
| Rehmannia glutinosa (Gaertn.) DC. [Orobanchaceae, Rehmanniae radix praeparata] | 6 |
| Conioselinum anthriscoides 'Chuanxiong' [Apiaceae, Chuanxiong rhizoma] | 5 |
| Angelica sinensis (Oliv.) Diels [Apiaceae, Angelicae sinensis radix] | 4 |
| Carthamus tinctorius L. [Asteraceae, Carthami flos] | 4 |
| Coix lacryma-jobi var. ma-yuen (Rom.Caill.) Stapf [Poaceae, Coicis semen] | 4 |
| Coptis chinensis Franch. [Ranunculaceae, Coptidis rhizoma] | 4 |
| Glycyrrhiza glabra L. [Fabaceae, Glycyrrhizae radix et rhizoma] | 4 |
| Glycyrrhiza uralensis Fisch. ex DC. [Fabaceae, Glycyrrhizae radix et rhizoma praeparata cum melle] | 4 |
| Litchi chinensis Sonn. [Sapindaceae, Litchi semen] | 4 |
| Ophiopogon japonicus (Thunb.) Ker Gawl. [Asparagaceae, Ophiopogonis radix] | 4 |
| Pueraria montana var. lobata (Willd.) Maesen & S.M.Almeida ex Sanjappa & Predeep [Fabaceae, Puerariae lobatae radix] | 4 |
| Rehmannia glutinosa (Gaertn.) DC. [Orobanchaceae, Rehmanniae Radix] | 4 |
| Rosa laevigata Michx. [Rosaceae, Rosae laevigatae fructus] | 4 |
| Trichosanthes kirilowii Maxim. [Cucurbitaceae, Trichosanthis radix] | 4 |
| Whitmania pigra Whitman [Hirudinidae, Hirudo] | 4 |
| Achyranthes bidentata Blume [Amaranthaceae, Achyranthis bidentatae radix] | 3 |
| Atractylodes lancea (Thunb.) DC. [Asteraceae, Atractylodis rhizoma] | 3 |
| Bombyx mori Linnaeus [Bombycidae, Bombyx batryticatus] | 3 |
| Codonopsis pilosula (Franch.) Nannf. [Campanulaceae, Codonopsis radix] | 3 |
| Epimedium sagittatum (Siebold & Zucc.) Maxim. [Berberidaceae, Epimedii folium] | 3 |
| Gallus gallus domesticus Brisson [Phasianidae, Galli gigerii endothelium corneum] | 3 |
| Ligustrum lucidum W.T.Aiton [Oleaceae, Ligustri lucidi fructus] | 3 |
| Polygonatum sibiricum Redouté [Asparagaceae, Polygonati rhizoma] | 3 |
| Pseudostellaria heterophylla (Miq.) Pax [Caryophyllaceae, Pseudostellariae radix] | 3 |
| Schisandra chinensis (Turcz.) Baill. [Schisandraceae, Schisandrae chinensis fructus] | 3 |
| Scrophularia ningpoensis Hemsl. [Scrophulariaceae, Scrophulariae radix] | 3 |
| Aconitum carmichaelii Debeaux [Ranunculaceae, Aconiti lateralis radix praeparata] | 2 |
| Boswellia frereana Birdw. [Burseraceae, Olibanum] | 2 |
| Citrus × aurantium L. [Rutaceae, Aurantii fructus] | 2 |
| Leonurus japonicus Houtt. [Lamiaceae, Leonuri herba] | 2 |
| Neolitsea cassia (L.) Kosterm. [Lauraceae, Cinnamomi ramulus] | 2 |
| Panax quinquefolius L. [Araliaceae, Panacis quinquefolii radix] | 2 |
| Plantago asiatica L. [Plantaginaceae, Plantaginis semen] | 2 |
| Spatholobus suberectus Dunn [Fabaceae, Spatholobi caulis] | 2 |
| Zingiber officinale Roscoe [Zingiberaceae, Zingiberis rhizoma recens] | 2 |
| Ziziphus jujuba Mill. [Rhamnaceae, Jujubae fructus] | 2 |
| Aquilaria sinensis (Lour.) Spreng. [Thymelaeaceae, Aquilariae lignum resinatum] | 1 |
| Buthus martensii Karsch [Buthidae, Scorpio] | 1 |
| Cryptotympana pustulata Fabricius [Cicadidae, Cicadae periostracum] | 1 |
| Cuscuta chinensis Lam. [Convolvulaceae, Cuscutae semen] | 1 |
| Dendrobium nobile Lindl. [Orchidaceae, Dendrobii caulis] | 1 |
| Dolomiaea costus (Falc.) Kasana & A.K.Pandey [Asteraceae, Aucklandiae radix] | 1 |
| Eclipta prostrata (L.) L. [Asteraceae, Ecliptae herba] | 1 |
| Euonymus alatus (Thunb.) Siebold [Celastraceae, Euonymus alatus] | 1 |
| Fossil fragments | 1 |
| Fossil fragments 15g, Ostrea gigas Thunberg [ostreidae, Ostreae concha] | 1 |
| Gardenia jasminoides J.Ellis [Rubiaceae, Gardeniae fructus] | 1 |
| Lindera aggregata (Sims) Kosterm. [Lauraceae, Linderae radix] | 1 |
| Monascus | 1 |
| Monascus 30g, Salvia miltiorrhiza Bunge [Lamiaceae, Salviae miltiorrhizae radix et rhizoma] | 1 |
| Ostrea gigas Thunberg [ostreidae, Ostreae concha] | 1 |
| Paecilomyces cicadae (Miquel.) Samson 20g, Salvia miltiorrhiza Bunge [Lamiaceae, Salviae miltiorrhizae radix et rhizoma] | 1 |
| Paeonia × suffruticosa Andrews [Paeoniaceae, Moutan cortex] | 1 |
| Paeonia lactiflora Pall. [Paeoniaceae, Paeoniae radix rubra] | 1 |
| Panax ginseng C.A.Mey. [Araliaceae, Ginseng radix et rhizoma] | 1 |
| Pheretima aspergillum (E.Perrier) [Megascolecidae, Pheretima] | 1 |
| Prunus persica (L.) Batsch [Rosaceae, Persicae semen] | 1 |
| Rheum palmatum L. [Polygonaceae, Rhei radix et rhizoma] | 1 |
| Smilax glabra Roxb. [Smilacaceae, Smilacis glabrae rhizoma] | 1 |
| Tetrapanax papyrifer (Hook.) K.Koch [Araliaceae, Tetrapanacis medulla] | 1 |
| Vigna umbellata (Thunb.) Ohwi & H.Ohashi [Fabaceae, Vignae semen] | 1 |
| Zea mays L. [Poaceae, corn silk] | 1 |
