# Supplementary material for: Mechanism of traditional Chinese medicine in elderly diabetes mellitus and a systematic review of its clinical application
Source: Front Pharmacol. 2024 Mar 6;15:1339148. doi: 10.3389/fphar.2024.1339148 (PMC10953506; doi:10.3389/fphar.2024.1339148)
Supplement: Supplementary file 2 [file DataSheet1.zip › Supplementary Table S1-17/Supplementary Table S6b.docx]

Supplementary Table S6b | Frequency of Traditional Chinese Medicine for the treatment of elderly DKD in Traditional Chinese patent medicines.

| Traditional Chinese Medicine | Frequency |
| --- | --- |
| Cordyceps sinensis（BerK.）Sacc. [Clavicipitaceae, Cordyceps] | 16 |
| Salvia miltiorrhiza Bunge [Lamiaceae, Salviae miltiorrhizae radix et rhizoma] | 12 |
| Alisma plantago-aquatica subsp. orientale (Sam.) Sam. [Alismataceae, Alismatis rhizoma] | 9 |
| Panax ginseng C.A.Mey. [Araliaceae, Ginseng radix et rhizoma] | 9 |
| Poria cocos(Schw.)Wolf Poria [Polyporaceae, Poria] | 8 |
| Astragalus mongholicus Bunge [Fabaceae, Astragali radix] | 7 |
| Atractylodes macrocephala Koidz. [Asteraceae, Atractylodis macrocephalae rhizoma] | 6 |
| Borneolum syntheticum | 6 |
| Panax notoginseng (Burkill) F.H.Chen [Araliaceae, Notoginseng radix et rhizoma] | 6 |
| Coptis chinensis Franch. [Ranunculaceae, Coptidis rhizoma] | 5 |
| Imperata cylindrica (L.) Raeusch. [Poaceae, Imperatae rhizoma] | 5 |
| Paeonia lactiflora Pall. [Paeoniaceae, Paeoniae radix alba] | 5 |
| Rehmannia glutinosa (Gaertn.) DC. [Orobanchaceae, Rehmanniae Radix] | 5 |
| Angelica biserrata (R.H.Shan & C.Q.Yuan) C.Q.Yuan & R.H.Shan [Apiaceae, Angelicae pubescentis radix] | 4 |
| Bupleurum chinense DC. [Apiaceae, BUPLEURI RADIX] | 4 |
| Citrus reticulata Blanco [Rutaceae, Citri reticulatae pericarpium] | 4 |
| Dioscorea oppositifolia L. [Dioscoreaceae, Dioscoreae rhizoma] | 4 |
| Eucommia ulmoides Oliv. [Eucommiaceae, Eucommiae cortex] | 4 |
| Glycine max (L.) Merr. [Fabaceae, Sojae semen nigrum] | 4 |
| Glycyrrhiza uralensis Fisch. ex DC. [Fabaceae, Glycyrrhizae radix et rhizoma praeparata cum melle] | 4 |
| Hansenia weberbaueriana (Fedde ex H.Wolff) Pimenov & Kljuykov [Apiaceae, Notopterygii rhizoma et radix] | 4 |
| Leonurus japonicus Houtt. [Lamiaceae, Leonuri herba] | 4 |
| Panax quinquefolius L. [Araliaceae, Panacis quinquefolii radix] | 4 |
| Pinellia ternata (Thunb.) Makino [Araceae, Pinelliae rhizoma] | 4 |
| Platycodon grandiflorus (Jacq.) A.DC. [Campanulaceae, Platycodonis radix] | 4 |
| Saposhnikovia divaricata (Turcz. ex Ledeb.) Schischk. [Apiaceae, Saposhnikoviae radix] | 4 |
| Scleromitrion diffusum (Willd.) R. J. Wang [Rubiaceae, Hedyotis diffusa] | 4 |
| Smilax glabra Roxb. [Smilacaceae, Smilacis glabrae rhizoma] | 4 |
| Zingiber officinale Roscoe [Zingiberaceae, Zingiberis rhizoma recens] | 4 |
| Ziziphus jujuba Mill. [Rhamnaceae, Jujubae fructus] | 4 |
| Abelmoschus manihot (L.) Medik. [Malvaceae, Abelmoschi corolla] | 3 |
| Reynoutria multiflora (Thunb.) Moldenke [Polygonaceae, Polygoni multiflori radix] | 3 |
| Anemarrhena asphodeloides Bunge [Asparagaceae, Anemarrhenae rhizoma] | 1 |
| Atractylodes lancea (Thunb.) DC. [Asteraceae, Atractylodis rhizoma] | 1 |
| Cistanche deserticola Ma [Orobanchaceae, Cistanches herba] | 1 |
| Codonopsis pilosula (Franch.) Nannf. [Campanulaceae, Codonopsis radix] | 1 |
| Cornus officinalis Siebold & Zucc. [Cornaceae, Corni fructus] | 1 |
| Cuscuta chinensis Lam. [Convolvulaceae, Cuscutae semen] | 1 |
| Epimedium sagittatum (Siebold & Zucc.) Maxim. [Berberidaceae, Epimedii folium] | 1 |
| Eupatorium fortunei Turcz. [Asteraceae, Eupatorii herba] | 1 |
| Glycyrrhiza glabra L. [Fabaceae, Glycyrrhizae radix et rhizoma] | 1 |
| Juncus effusus L. [Juncaceae, Junci medulla] | 1 |
| Litchi chinensis Sonn. [Sapindaceae, Litchi semen] | 1 |
| Lonicera japonica Thunb. [Caprifoliaceae, Lonicerae japonicae flos] | 1 |
| Lophatherum gracile Brongn. [Poaceae, Lophatheri herba] | 1 |
| Lycium barbarum L. [Solanaceae, Lycii cortex] | 1 |
| Morindae officinalis radix [Rubiaceae, Morindae officinalis radix] | 1 |
| Morus alba L. [Moraceae, Mori cortex] | 1 |
| Ophiopogon japonicus (Thunb.) Ker Gawl. [Asparagaceae, Ophiopogonis radix] | 1 |
| Phellodendron chinense C.K.Schneid. [Rutaceae, Phellodendri chinensis cortex] | 1 |
| Plantago asiatica L. [Plantaginaceae, Plantaginis herba] | 1 |
| Plantago asiatica L. [Plantaginaceae, Plantaginis semen] | 1 |
| Polygonatum sibiricum Redouté [Asparagaceae, Polygonati rhizoma] | 1 |
| Pueraria montana var. lobata (Willd.) Maesen & S.M.Almeida ex Sanjappa & Predeep [Fabaceae, Puerariae lobatae radix] | 1 |
| Rheum palmatum L. [Polygonaceae, Rhei radix et rhizoma] | 1 |
| Schisandra chinensis (Turcz.) Baill. [Schisandraceae, Schisandrae chinensis fructus] | 1 |
| Sophora flavescens Aiton [Fabaceae, Sophorae flavescentis radix] | 1 |
| BoenninghauseniasessilicarpaLevl. | 1 |
| Orthosiphon aristatus (Blume) Miq. [Lamiaceae, java tea] | 1 |
