# Supplementary material for: Mechanism of traditional Chinese medicine in elderly diabetes mellitus and a systematic review of its clinical application
Source: Front Pharmacol. 2024 Mar 6;15:1339148. doi: 10.3389/fphar.2024.1339148 (PMC10953506; doi:10.3389/fphar.2024.1339148)
Supplement: Supplementary file 2 [file DataSheet1.zip › Supplementary Table S1-17/Supplementary Table S7.docx]

Supplementary Table S7 | Interventional drugs composition of TCM for elderly DR.

| Study | Interventional drugs composition |
| --- | --- |
| Traditional Chinese Prescription | |
| Li 2022 (2) | Danhuang Mingmu Decoction: Rehmannia glutinosa (Gaertn.) DC. [Orobanchaceae, Rehmanniae Radix] 15g, Imperata cylindrica (L.) Raeusch. [Poaceae, Imperatae rhizoma] 15g, Paeonia × suffruticosa Andrews [Paeoniaceae, Moutan cortex] 15g, Ophiopogon japonicus (Thunb.) Ker Gawl. [Asparagaceae, Ophiopogonis radix] 10g, Plantago asiatica L. [Plantaginaceae, Plantaginis semen] 10g, Ziziphus jujuba Mill. [Rhamnaceae, Ziziphi spinosae semen] 10g, Reynoutria multiflora (Thunb.) Moldenke [Polygonaceae, Polygoni multiflori caulis] 10g, Salvia miltiorrhiza Bunge [Lamiaceae, Salviae miltiorrhizae radix et rhizoma] 8g, Fushen 8g, Coptis chinensis Franch. [Ranunculaceae, Coptidis rhizoma] 3g, Rheum palmatum L. [Polygonaceae, Rhei radix et rhizoma] 3g |
| Wei 2012 | Zhenwu Decoction: Aconitum carmichaelii Debeaux [Ranunculaceae, Aconiti lateralis radix praeparata] 15g, Atractylodes macrocephala Koidz. [Asteraceae, Atractylodis macrocephalae rhizoma] 30g, Poria cocos(Schw.)Wolf Poria [Polyporaceae, Poria] 30g, Epimedium sagittatum (Siebold & Zucc.) Maxim. [Berberidaceae, Epimedii folium] 20g, Zingiber officinale Roscoe [Zingiberaceae, Zingiberis rhizoma recens] 20g, Astragalus mongholicus Bunge [Fabaceae, Astragali radix] 20g, Salvia miltiorrhiza Bunge [Lamiaceae, Salviae miltiorrhizae radix et rhizoma] 20g, Conioselinum anthriscoides 'Chuanxiong' [Apiaceae, Chuanxiong rhizoma] 15g |
| Liu 2011 | Zhenwu Decoction: Aconitum carmichaelii Debeaux [Ranunculaceae, Aconiti lateralis radix praeparata] 15g, Atractylodes macrocephala Koidz. [Asteraceae, Atractylodis macrocephalae rhizoma] 30g, Poria cocos(Schw.)Wolf Poria [Polyporaceae, Poria] 30g, Epimedium sagittatum (Siebold & Zucc.) Maxim. [Berberidaceae, Epimedii folium] 20g, Zingiber officinale Roscoe [Zingiberaceae, Zingiberis rhizoma recens] 20g, Astragalus mongholicus Bunge [Fabaceae, Astragali radix] 20g, Salvia miltiorrhiza Bunge [Lamiaceae, Salviae miltiorrhizae radix et rhizoma] 20g, Conioselinum anthriscoides 'Chuanxiong' [Apiaceae, Chuanxiong rhizoma] 15g |
| Traditional Chinese patent medicines | |
| Wang 2020 | Compound Xueshuantong Capsules: Panax notoginseng (Burkill) F.H.Chen [Araliaceae, Notoginseng radix et rhizoma] 250g, Astragalus mongholicus Bunge [Fabaceae, Astragali radix] 80g, Salvia miltiorrhiza Bunge [Lamiaceae, Salviae miltiorrhizae radix et rhizoma] 50g, Scrophularia ningpoensis Hemsl. [Scrophulariaceae, Scrophulariae radix] 80g |
| Yan 2014 | Compound Danshen Dripping Pills: Salvia miltiorrhiza Bunge [Lamiaceae, Salviae miltiorrhizae radix et rhizoma] 90g, Panax notoginseng (Burkill) F.H.Chen [Araliaceae, Notoginseng radix et rhizoma] 17.6g, Borneolum syntheticum 1g |
| Zhang 2012 | Qiju Dihuang Pills: Lycium barbarum L. [Solanaceae, Lycii fructus] 40g, Chrysanthemum × morifolium (Ramat.) Hemsl. [Asteraceae, Chrysanthemi flos] 40g, Rehmannia glutinosa (Gaertn.) DC. [Orobanchaceae, Rehmanniae radix praeparata] 160g, Cornus officinalis Siebold & Zucc. [Cornaceae, Corni fructus] 80g, Paeonia × suffruticosa Andrews [Paeoniaceae, Moutan cortex] 60g, Dioscorea oppositifolia L. [Dioscoreaceae, Dioscoreae rhizoma] 80g, Poria cocos(Schw.)Wolf Poria [Polyporaceae, Poria] 60g, Alisma plantago-aquatica subsp. orientale (Sam.) Sam. [Alismataceae, Alismatis rhizoma] 60g |
