# Supplementary material for: Mechanism of traditional Chinese medicine in elderly diabetes mellitus and a systematic review of its clinical application
Source: Front Pharmacol. 2024 Mar 6;15:1339148. doi: 10.3389/fphar.2024.1339148 (PMC10953506; doi:10.3389/fphar.2024.1339148)
Supplement: Supplementary file 2 [file DataSheet1.zip › Supplementary Table S1-17/Supplementary Table S8.docx]

Supplementary Table S8 | Interventional drugs composition of TCM for elderly DPN.

| Study | Interventional drugs composition |
| --- | --- |
| Traditional Chinese Prescription | |
| Yang 2019 | Modified Buyang Huanwu Decoction: Astragalus mongholicus Bunge [Fabaceae, Astragali radix] 30g, Spatholobus suberectus Dunn [Fabaceae, Spatholobi caulis] 30g, Angelica sinensis (Oliv.) Diels [Apiaceae, Angelicae sinensis radix] 15g, Pheretima aspergillum (E.Perrier) [Megascolecidae, Pheretima] 15g, Conioselinum anthriscoides 'Chuanxiong' [Apiaceae, Chuanxiong rhizoma] 15g, Rehmannia glutinosa (Gaertn.) DC. [Orobanchaceae, Rehmanniae Radix] 15g, Liquidambar formosana Hance [Altingiaceae, Liquidambaris fructus] 15g, Paeonia lactiflora Pall. [Paeoniaceae, Paeoniae radix alba] 15g, Carthamus tinctorius L. [Asteraceae, Carthami flos] 10g, Prunus persica (L.) Batsch [Rosaceae, Persicae semen] 10g |
| Xu 2017 | Yangyinhuoxue decoction: Dendrobium nobile Lindl. [Orchidaceae, Dendrobii caulis] 20g, Scrophularia ningpoensis Hemsl. [Scrophulariaceae, Scrophulariae radix] 15g, Achyranthes bidentata Blume [Amaranthaceae, Achyranthis bidentatae radix] 20g, Biancaea sappan (L.) Tod. [Fabaceae, Sappan lignum] 12g, Siphonostegia chinensis Benth. [Orobanchaceae, Siphonostegiae herba] 10g, Spatholobus suberectus Dunn [Fabaceae, Spatholobi caulis] 30g, Paeonia lactiflora Pall. [Paeoniaceae, Paeoniae radix rubra] 10g, Clematis chinensis Osbeck [Ranunculaceae, Clematidis radix et rhizoma] 15g, Boswellia frereana Birdw. [Burseraceae, Olibanum] 10g, Commiphora myrrha (T.Nees) Engl. [Burseraceae, Myrrha] 10g, Angelica sinensis (Oliv.) Diels [Apiaceae, Angelicae sinensis radix] 10g |
| Li 2016 (2) | Modified Huangqi Guizhi Wuwu Decoction: Astragalus mongholicus Bunge [Fabaceae, Astragali radix], Neolitsea cassia (L.) Kosterm. [Lauraceae, Cinnamomi ramulus], Spatholobus suberectus Dunn [Fabaceae, Spatholobi caulis], Prunus persica (L.) Batsch [Rosaceae, Persicae semen], Achyranthes bidentata Blume [Amaranthaceae, Achyranthis bidentatae radix], Carthamus tinctorius L. [Asteraceae, Carthami flos], Paeonia lactiflora Pall. [Paeoniaceae, Paeoniae radix rubra], Luffa cylindrica（L.）Roem. Luffae fructus retinervus] |
| Guo 2016 | Compound Qiteng Tongluo Decpction: Astragalus mongholicus Bunge [Fabaceae, Astragali radix] 30g, Spatholobus suberectus Dunn [Fabaceae, Spatholobi caulis] 25g, Chaenomeles speciosa (Sweet) Nakai [Rosaceae, Chaenomelis fructus] 15g, Coix lacryma-jobi var. ma-yuen (Rom.Caill.) Stapf [Poaceae, Coicis semen] 15g, Atractylodes lancea (Thunb.) DC. [Asteraceae, Atractylodis rhizoma] 15g, Tetrapanax papyrifer (Hook.) K.Koch [Araliaceae, Tetrapanacis medulla] 15g, Benincasa hispida（Thunb.）Cogn. [Cucurbitaceae, wax gourd seed] 15g, Morus alba L. [Moraceae, Mori ramulus] 15g, Strobilanthes cusia (Nees) Kuntze [Acanthaceae, Indigo naturalis] 15g, Dioscorea spongiosa J.Q.Xi, M.Mizuno & W.L.Zhao [Dioscoreaceae, Dioscoreae spongiosae rhizoma] 15g, Achyranthes bidentata Blume [Amaranthaceae, Achyranthis bidentatae radix] 15g, Phellodendron chinense C.K.Schneid. [Rutaceae, Phellodendri chinensis cortex] 15g |
| Li 2012 | Yiqi Huoxue Tongluo Recipe: Astragalus mongholicus Bunge [Fabaceae, Astragali radix] 40g, Neolitsea cassia (L.) Kosterm. [Lauraceae, Cinnamomi ramulus] 15g, Paeonia lactiflora Pall. [Paeoniaceae, Paeoniae radix alba] 10g, Paeonia lactiflora Pall. [Paeoniaceae, Paeoniae radix rubra] 10g, Angelica sinensis (Oliv.) Diels [Apiaceae, Angelicae sinensis radix] 10g, Pheretima aspergillum (E.Perrier) [Megascolecidae, Pheretima] 10g, Prunus persica (L.) Batsch [Rosaceae, Persicae semen] 10g, Conioselinum anthriscoides 'Chuanxiong' [Apiaceae, Chuanxiong rhizoma] 10g, Carthamus tinctorius L. [Asteraceae, Carthami flos] 10g, Buthus martensii Karsch [Buthidae, Scorpio] 5g, Whitmania pigra Whitman [Hirudinidae, Hirudo] 6g, Zingiber officinale Roscoe [Zingiberaceae, Zingiberis rhizoma recens] 6g, Ziziphus jujuba Mill. [Rhamnaceae, Jujubae fructus] 6g, Glycyrrhiza glabra L. [Fabaceae, Glycyrrhizae radix et rhizoma] 5g |
| Traditional Chinese Medicine Extracts | |
| Wang 2009 | Berberine |
