# Supplementary material for: Mechanism of traditional Chinese medicine in elderly diabetes mellitus and a systematic review of its clinical application
Source: Front Pharmacol. 2024 Mar 6;15:1339148. doi: 10.3389/fphar.2024.1339148 (PMC10953506; doi:10.3389/fphar.2024.1339148)
Supplement: Supplementary file 2 [file DataSheet1.zip › Supplementary Table S1-17/Supplementary Table S9.docx]

Supplementary Table S9 | Frequency of Traditional Chinese Medicine for the treatment of elderly DPN in Traditional Chinese Prescription.

| Traditional Chinese Medicine | Frequency |
| --- | --- |
| Astragalus mongholicus Bunge [Fabaceae, Astragali radix] | 4 |
| Spatholobus suberectus Dunn [Fabaceae, Spatholobi caulis] | 4 |
| Achyranthes bidentata Blume [Amaranthaceae, Achyranthis bidentatae radix] | 3 |
| Angelica sinensis (Oliv.) Diels [Apiaceae, Angelicae sinensis radix] | 3 |
| Carthamus tinctorius L. [Asteraceae, Carthami flos] | 3 |
| Paeonia lactiflora Pall. [Paeoniaceae, Paeoniae radix rubra] | 3 |
| Prunus persica (L.) Batsch [Rosaceae, Persicae semen] | 3 |
| Conioselinum anthriscoides 'Chuanxiong' [Apiaceae, Chuanxiong rhizoma] | 2 |
| Neolitsea cassia (L.) Kosterm. [Lauraceae, Cinnamomi ramulus] | 2 |
| Paeonia lactiflora Pall. [Paeoniaceae, Paeoniae radix alba] | 2 |
| Pheretima aspergillum (E.Perrier) [Megascolecidae, Pheretima] | 2 |
| Atractylodes lancea (Thunb.) DC. [Asteraceae, Atractylodis rhizoma] | 1 |
| Benincasa hispida（Thunb.）Cogn. [Cucurbitaceae, wax gourd seed] | 1 |
| Biancaea sappan (L.) Tod. [Fabaceae, Sappan lignum] | 1 |
| Boswellia frereana Birdw. [Burseraceae, Olibanum] | 1 |
| Buthus martensii Karsch [Buthidae, Scorpio] | 1 |
| Chaenomeles speciosa (Sweet) Nakai [Rosaceae, Chaenomelis fructus] | 1 |
| Clematis chinensis Osbeck [Ranunculaceae, Clematidis radix et rhizoma] | 1 |
| Coix lacryma-jobi var. ma-yuen (Rom.Caill.) Stapf [Poaceae, Coicis semen] | 1 |
| Commiphora myrrha (T.Nees) Engl. [Burseraceae, Myrrha] | 1 |
| Dendrobium nobile Lindl. [Orchidaceae, Dendrobii caulis] | 1 |
| Dioscorea spongiosa J.Q.Xi, M.Mizuno & W.L.Zhao [Dioscoreaceae, Dioscoreae spongiosae rhizoma] | 1 |
| Glycyrrhiza glabra L. [Fabaceae, Glycyrrhizae radix et rhizoma] | 1 |
| Liquidambar formosana Hance [Altingiaceae, Liquidambaris fructus] | 1 |
| Luffa cylindrica（L.）Roem. Luffae fructus retinervus] | 1 |
| Morus alba L. [Moraceae, Mori ramulus] | 1 |
| Phellodendron chinense C.K.Schneid. [Rutaceae, Phellodendri chinensis cortex] | 1 |
| Rehmannia glutinosa (Gaertn.) DC. [Orobanchaceae, Rehmanniae Radix] | 1 |
| Scrophularia ningpoensis Hemsl. [Scrophulariaceae, Scrophulariae radix] | 1 |
| Siphonostegia chinensis Benth. [Orobanchaceae, Siphonostegiae herba] | 1 |
| Strobilanthes cusia (Nees) Kuntze [Acanthaceae, Indigo naturalis] | 1 |
| Tetrapanax papyrifer (Hook.) K.Koch [Araliaceae, Tetrapanacis medulla] | 1 |
| Whitmania pigra Whitman [Hirudinidae, Hirudo] | 1 |
| Zingiber officinale Roscoe [Zingiberaceae, Zingiberis rhizoma recens] | 1 |
| Ziziphus jujuba Mill. [Rhamnaceae, Jujubae fructus] | 1 |
